# mbImpute: an accurate and robust imputation method for microbiome data

**DOI:** 10.1186/s13059-021-02400-4

**Published:** 2021-06-28

**Authors:** Ruochen Jiang, Wei Vivian Li, Jingyi Jessica Li

**Affiliations:** 1 Department of Statistics, University of California, Los Angeles, 90095-1554 CA USA; 2 Department of Biostatistics and Epidemiology, Rutgers School of Public Health, Piscataway, 08854 NJ USA; 3 Department of Human Genetics, University of California, Los Angeles, 90095-7088 CA USA; 4 Department of Computational Medicine, University of California, Los Angeles, 90095-1766 CA USA; 5 Department of Biostatistics, University of California, Los Angeles, 90095-1772 CA USA

## Abstract

A critical challenge in microbiome data analysis is the existence of many non-biological zeros, which distort taxon abundance distributions, complicate data analysis, and jeopardize the reliability of scientific discoveries. To address this issue, we propose the first imputation method for microbiome data—mbImpute—to identify and recover likely non-biological zeros by borrowing information jointly from similar samples, similar taxa, and optional metadata including sample covariates and taxon phylogeny. We demonstrate that mbImpute improves the power of identifying disease-related taxa from microbiome data of type 2 diabetes and colorectal cancer, and mbImpute preserves non-zero distributions of taxa abundances.

**Supplementary Information:**

The online version contains supplementary material available at (10.1186/s13059-021-02400-4).

## Introduction

Microbiome studies explore the collective genomes of microorganisms living in a certain environment such as soil, sea water, animal skin, and human gut. Numerous studies have confirmed the importance of microbiomes in natural environments and human bodies [1]. For example, new discoveries have revealed the important roles microbiomes play in complex diseases such as obesity [2], diabetes [3], pulmonary disease [4, 5], and cancers [6]. These studies have shown the potential of human microbes as biomarkers for disease diagnosis or as therapeutic targets for disease treatment [7].

The development of high-throughput sequencing technologies has advanced microbiome studies in the last decade [8]. Two sequencing technologies are primarily used: the 16S ribosomal RNA (rRNA) amplicon sequencing and the shotgun metagenomic sequencing. The 16S rRNA amplicon sequencing measures 16S rRNAs, which can be used to identify and distinguish microbes [9]. The 16S rRNA sequencing reads are either clustered into operational taxonomic units (OTUs) [10] or mapped to amplicon sequence variants (ASVs) [11, 12]. The shotgun metagenomic sequencing, also known as the whole-genome sequencing (WGS), sequences all DNAs in a microbiome sample, including whole genomes of microbial species and host DNAs [10, 13–19]. The WGS sequencing reads are mapped to known microbial genome databases to quantify the abundances of microbial species. Despite the vast differences between the two technologies, 16S and WGS data can both be processed into the same data structure containing abundances of microbes in microbiome samples: a taxon count matrix with rows as microbiome samples (which often correspond to subjects or individuals) and columns as taxa (i.e., OTUs or ASVs for 16S rRNA data and species for WGS data), and each entry corresponds to the number of reads mapped to a taxon in a microbiome sample. It is worth noting that the total read count per microbiome sample, i.e., the sum of entries in a row of the count matrix, differs by five orders of magnitude between the two technologies: ∼10^3^ per sample for 16S rRNA data and ∼10^8^ for WGS data [20].

A critical challenge in microbiome data analysis is the existence of many zeros in taxon counts, an ubiquitous phenomenon for both 16S rRNA and WGS data [20]. The large proportion of zeros belongs to three categories by origin: biological, technical, and sampling zeros [21]. Biological zeros represent true zero abundances of non-existent taxa in microbiome samples. In contrast, technical and sampling zeros are non-biological zeros with different origins: technical zeros arise from pre-sequencing experimental artifacts (e.g., DNA degradation during library preparation and inefficient sequence amplification due to factors such as GC content bias) [22], while sampling zeros are due to limited sequencing depths. Although WGS data have much larger per-sample total read counts than 16S data have, they still suffer from sampling zeros because they sequence more nucleic acid sequences (microbial genomes instead of 16S rRNAs) and their effective sequencing depths are reduced by widespread host DNA contaminations [23–25].

This data sparsity issue challenges microbiome data analysis, as most state-of-the-art methods have poor performance on data containing too many zeros. Adding a pseudo-count of one to zeros is a common, simple approach [26, 27], but it is ad hoc and suboptimal because it cannot distinguish biological zeros from technical and sampling zeros [28, 29]. Kaul et al. [30] developed an approach to distinguish these three types of zeros and to correct only the sampling zeros; however, their correction is still a simple addition of a pseudo-count of one, ignoring the fact that the (unobserved) actual counts of sampling zeros may not be exactly one.

In particular, this data sparsity issue hinders the differentially abundant (DA) taxon analysis, which aims to identify the taxa that exhibit significantly different abundances between two groups of samples [13]. Microbiome researchers employ two major types of statistical methods to identify DA taxa. Methods of the first type use parametric models [7, 26, 31–38]. For example, the zero-inflated negative binomial generalized linear model (ZINB-GLM) is used in [7, 31, 32], the negative binomial regression is used in the DESeq2-phyloseq method [33, 34], and the zero-inflated Gaussian model is used in the metagenomeSeq method [35]. However, these parametric model assumptions may not hold for a particular dataset [39]. Methods of the second type perform non-parametric statistical tests that do not assume specific data distributions. Widely used methods include the Wilcoxon rank-sum test [14–19] and ANCOM [27]. A major drawback of these non-parametric methods is that a taxon would be called DA if its zero proportions differ significantly between two groups of samples, but this difference is unlikely biologically meaningful due to the prevalence of technical and sampling zeros. Note that both types of DA methods require the input taxon abundances to be in one of three units: counts [7, 31, 32, 34], log-transformed counts [35], and proportions (i.e., each taxon’s count is divided by the sum of all taxa’s counts in a sample) [26, 27, 36–38]; regardless of the unit, DA taxon analysis is always biased by the prevalence of technical and sampling zeros.

In addition to DA taxon analysis, other microbiome data analyses, such as the construction of taxon interaction networks [40–43], are also impeded by the data sparsity challenge. Although zero-inflated modeling is commonly used for sparse data, it requires a specific model formulation for each analysis task, which is often complicated or unrealistic for most microbiome researchers. Hence, a flexible and robust approach is needed to address the sparsity issue of microbiome data.

Imputation is a widely used technique to recover missing data and facilitate data analysis. It has successful applications in many fields, e.g., recommender systems (e.g., the Netflix challenge [44]), image and speech reconstruction [45–47], imputation of unmeasured epigenomics datasets [48], missing genotype prediction in genome-wide association studies [49], and the more recent gene expression recovery in single-cell RNA-sequencing (scRNA-seq) data [50–54]. Microbiome and scRNA-seq data have the same count matrix structure if one considers microbiome samples and taxa as analogs to cells and genes, respectively; both data have large proportions of non-biological zeros. Given the successes of scRNA-seq imputation methods, we hypothesize that imputation can also relieve the data sparsity issue in microbiome data. Although there are methods utilizing matrix completion in the microbiome field, their main purpose is to perform community detection or dimension reduction instead of imputation [55, 56]. Two distinct features of microbiome data make it suboptimal to directly apply existing imputation methods. First, microbiome data are often accompanied by metadata including sample covariates and taxon phylogeny, which, however, cannot be used by existing imputation methods. In particular, phylogenetic information is known to be valuable for microbiome data analysis [57–64], as closely-related taxa in a phylogeny are likely to have similar functions and abundances in samples [65–68]. Second, microbiome data have a much smaller number of samples (often in hundreds) than the number of cells (often in tens of thousands) in scRNA-seq data, making those deep-learning based imputation methods inapplicable [54, 69]. On the other hand, the smaller sample size allows microbiome data to afford an imputation method that focuses more on imputation accuracy than computational time.

**Fig. 1:**
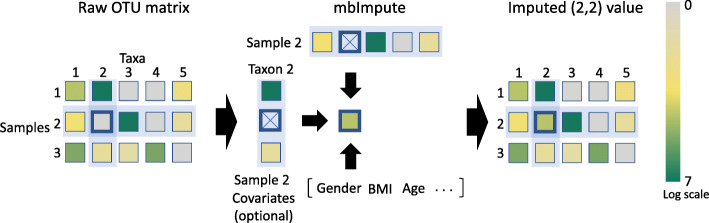
An illustration of mbImpute. After mbImpute identifies likely non-biological zeros, it imputes them (e.g., the abundance of taxon 2 in sample 2) by jointly borrowing information from similar samples, similar taxa, and sample covariates if available (details in Methods)

Here, we propose mbImpute, the first imputation method designed for microbiome data including both 16S and WGS data. The mbImpute method identifies and corrects the zeros and low counts that are unlikely biological (for ease of terminology, we will refer to them as non-biological zeros in the following text) in microbiome taxon count data. The goal of mbImpute is to provide a principled data-driven approach to relieve the microbiome data sparsity issue due to prevalent non-biological zeros. To achieve this, mbImpute leverages three sources of information: a taxon count matrix, sample covariates (e.g., sample library size and subjects’ age, gender, and body mass index), and taxon phylogeny, with the latter two sources being optional. There are two main steps in mbImpute (Fig. 1): first, mbImpute identifies likely non-biological zeros; second, it imputes these zeros by borrowing information from similar taxa (determined by both phylogeny and counts), similar microbiome samples (in terms of taxon counts), and sample covariates if available (see an illustration of the imputation step in Additional file 1: Figure S1). The imputed data are expected to contain recovered taxon counts and would thus facilitate various downstream analyses, such as the identification of DA taxa and the construction of taxon interaction networks. Microbiome researchers can use mbImpute to avoid the hassle of dealing with sparse data in individual analysis tasks and to enjoy the flexibility of building up data analysis pipelines.

## Results

### mbImpute outperforms non-microbiome imputation methods in recovering missing taxon abundances and empowering DA taxon identification

**Fig. 2:**
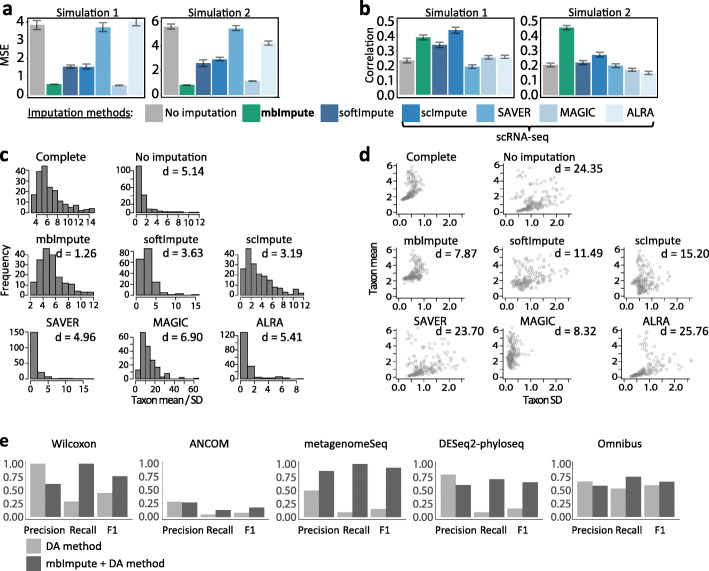
mbImpute outperforms state-of-the-art imputation methods designed for non-microbiome data and enhances the identification of DA taxa. **a** Mean squared error (MSE) and **b** mean Pearson correlation of taxon abundances between the complete data and the zero-inflated data (“No imputation,” the baseline) or the imputed data by each imputation method (mbImpute, softImpute, scImpute, SAVER, MAGIC, and ALRA) in Simulations 1 and 2 (see Additional file 1). **c**–**d** For each taxon, the mean and standard deviation (SD) of its abundances are calculated for the complete data, the zero-inflated data, and the imputed data by each imputation method in Simulation 1; **c** shows the distributions of the taxon mean/SD and the Wasserstein distance between every distribution and the complete distribution; **d** the taxa in two coordinates, mean vs. SD, and the average Euclidean distance between the taxa in every (zero-inflated or imputed) dataset and the complete data in these two coordinates. **e** Accuracy (precision, recall, and F_1_ scores) of five DA methods (Wilcoxon rank-sum test, ANCOM, metagenomeSeq, DESeq2-phyloseq, and Omnibus test) with the FDR threshold 0.05 on raw data (light color) and imputed data by mbImpute (dark color) in the 16S data simulation

As there are no imputation methods for microbiome data, we benchmark mbImpute against five state-of-the-art imputation methods designed for non-microbiome data: four popular scRNA-seq imputation methods (scImpute [50], SAVER [52], MAGIC [51], and ALRA [53]) and a widely used general imputation method softImpute [70]. We design two simulation studies, and the common goal is to obtain a “complete” microbiome dataset without non-biological zeros, so that we can evaluate imputation accuracy by comparing the imputed data with the complete data. In the first study, we simulate complete data from a generative model fitted to a WGS dataset of type 2 diabetes (T2D) samples [18]; In the second, more realistic simulation study, we extract a sub-dataset with fewer than 15% zeros as the complete data from another WGS dataset of T2D samples [19]. In both simulation studies (see Additional file 1: Simulation 1 and Simulation 2 [1–3, 6–8, 10, 13–19, 26, 27, 30–32, 50, 52, 54, 70–101]), we introduce non-biological zeros into the complete data by mimicking the observed zero patterns in real datasets, obtaining what we call the zero-inflated data. After applying the six imputation methods to the zero-inflated data in both studies, we compare these methods’ imputation accuracy in three aspects: (1) the mean squared error (MSE) between the imputed data and the complete data, (2) each taxon’s Pearson correlation between its imputed abundances and complete abundances, and (3) the Wasserstein distance between the distributions of taxa’s abundance mean/(standard deviation) ratios in the imputed data and the complete data. Figure 2a–d illustrate the comparison results, indicating that mbImpute achieves the best overall performance in all three aspects. In particular, Fig. 2c–d and Additional file 1: Figure S2 show that the imputed data by mbImpute best resemble the complete data, verifying the advantage of mbImpute in recovering missing taxon abundances in microbiome data.

We next demonstrate that mbImpute is a robust method. The core of mbImpute is to borrow three-way information from similar samples, similar taxa, and sample covariates to impute non-biological zeros in microbiome data (see Methods). In the aforementioned second simulation study (Additional file 1: Simulation 2), we scramble samples in the real T2D WGS data when we select the complete data, a situation not optimal for mbImpute; however, mbImpute still outperforms existing imputation methods (Fig. 2a, b). To further test for the robustness of mbImpute, we design a third simulation study including four simulation schemes, where the information useful for imputation is encoded in sample covariates only, samples only, taxa only, or three sources together (see Additional file 1: Simulation 3). Additional file 1: Figure S3 shows that mbImpute effectively recovers non-biological zeros and reduces the MSE under every scheme. These results verify the robustness of mbImpute in selectively leveraging the information useful for imputation.

To further evaluate the performance of mbImpute on 16S rRNA sequencing data, we use a 16S simulator sparseDOSSA [89] to generate the abundances of 150 taxa in 100 samples under two conditions (see Additional file 1: Simulation 4). Among these 150 taxa, 45 are predefined as truly DA taxa. We apply five state-of-the-art DA methods: the Wilcoxon rank-sum test, ANCOM [27], metagenomeSeq [35], DESeq2-phyloseq [33, 34], and Omnibus test [102]. To evaluate the accuracy of DA taxon identification, we calculate the precision, recall, and F_1_ score (i.e., the harmonic mean of precision and recall) of each method, with or without using mbImpute as a preceding step, by comparing each method’s detected DA taxa to the truly DA taxa. Note that metagenomeSeq uses the zero-inflated Gaussian linear model for log-transformed microbiome data, but this model does not fit well to imputed data, which have many zeros removed; hence, we use the Gaussian linear model without zero-inflation to evaluate metagenomeSeq on imputed data. Under the false discovery rate (FDR) thresholds of 0.05 (Fig. 2e) and 0.1 (Additional file 1: Figure S4), the mbImpute-empowered DA methods consistently have better recall rates and F_1_ scores than those of the same DA methods without imputation. Notably, mbImpute improves both precision and recall rates of metagenomeSeq.

To evaluate the robustness of mbImpute to sequencing depth, we simulate 16S rRNA sequencing data based on real data for 300 taxa in 54 samples with four sequencing depths: 1000, 2000, 5000, and 10,000 reads per sample (see Additional file 1: Simulation 5). Additional file 1: Figure S5a shows that mbImpute has better imputation accuracy as sequencing depth increases. This is an expected result because a larger sequencing depth leads to fewer missing data so that mbImpute can be better trained with more non-missing data. We further evaluate the performance of the five non-microbiome imputation methods along with mbImpute. Additional file 1: Figure S6 shows that softImpute and ALRA, the two low-rank matrix factorization methods, also have better imputation accuracy as sequencing depth increases, yet their accuracies are worse than those of mbImpute at all sequencing depths. Unexpectedly, the four other imputation methods developed for scRNA-seq data—SAVER, scImpute, MAGIC, and ALRA—show no improvement over the baseline, “no imputation.” One possible reason is that the sequencing depths used in this simulation (∼10^3^) are much lower than those of typical scRNA-seq data (∼10^6^). These results again suggest that scRNA-seq imputation methods are unsuitable for microbiome 16S rRNA sequencing data. We also check the robustness of mbImpute to outlier samples. Taking the sample with the 2000-read per-sample sequencing depth, we generate one or two outlier samples by assigning large abundance values to 62 lowly abundant taxa in the existing 54 samples and setting other taxa’s abundance to zero (see Additional file 1: Simulation 5). Additional file 1: Figure S5b shows that the imputation accuracy of mbImpute is robust to the introduction of outlier samples. Additional file 1: Figure S7 shows the abundance distributions of four example taxa with outlier values before and after imputation. We observe that the existence of outliers does not distort the post-imputation distribution of non-outlier samples.

### mbImpute empowers DESeq2-phyloseq in DA taxon analysis

We find that mbImpute works well with DESeq2-phyloseq [33, 34], a widely used DA method for microbiome data, on real WGS datasets. We perform DA analysis on two T2D WGS datasets [18, 19] and four CRC WGS datasets [14–17], with or without using mbImpute as a preceding step. The goal of DA analysis is to identify the DA taxa between the diseased and control samples. These DA taxa may serve as potential targets for early detection or treatment of disease [14]. Note that mbImpute does not utilize the samples’ group information (whether each sample belongs to the diseased or control group) for its imputation, so that mbImpute will not falsely increase sample similarity within groups.

We start with the five DA methods—Wilcoxon rank-sum test, ANCOM, metagenomeSeq, DESeq2-phyloseq, and Omnibus test—for identifying disease-related DA taxa in the two T2D and four CRC datasets. Under the FDR threshold 0.05, only DESeq2-phyloseq and Omnibus test identify DA taxa in all datasets (Additional file 1: Table S1). Hence, we focus on evaluating the accuracy of DESeq2-phyloseq and Omnibus test on the original and imputed data (for DESeq2-phyloseq applied to the imputed data, we refer to it as mbImpute-empowered DESeq2-phyloseq). For a sanity check on the DA taxon identification results in each dataset, we plot the distribution of taxa’s *p* values calculated by DESeq2-phyloseq or Omnibus test before and after mbImpute is applied (Additional file 1: Figures S8–9). We find that all the *p* value distributions for DESeq2-phyloseq match our expectation (i.e., the expected *p* value distribution should have a mode near zero and be uniform elsewhere). However, the *p* value distributions for Omnibus test exhibit abnormality for the Karlsson et al. T2D dataset [18] and Vogtmann et al. CRC dataset [17]. Specifically, the distributions have an unexpected mode near one for the Karlsson et al. T2D dataset [18] after imputation and for the Vogtmann et al. CRC dataset [17] before and after imputation. This phenomenon suggests that the distributional assumption of Omnibus test does not hold for these data. Hence, we focus on the comparison between DESeq2-phyloseq and mbImpute-empowered DESeq2-phyloseq in the following analysis.

**Fig. 3:**
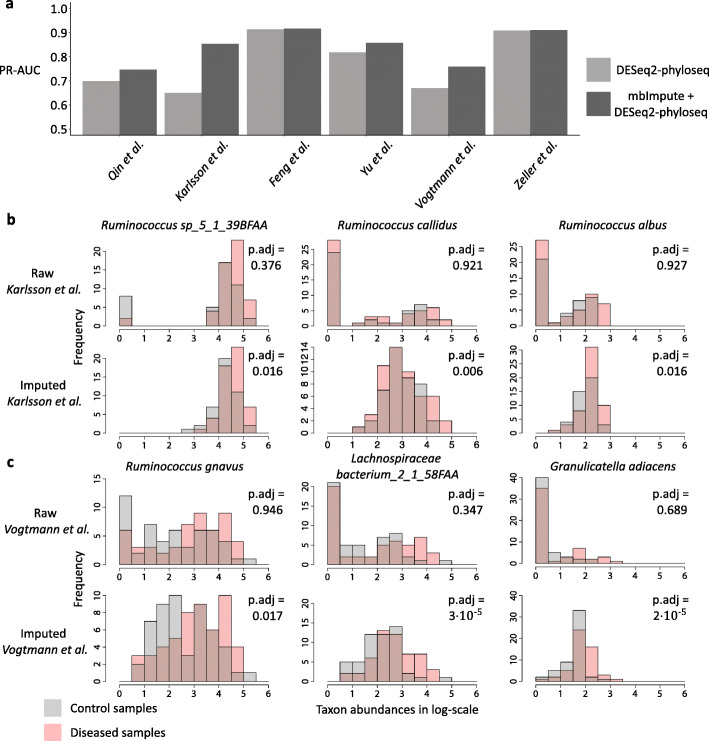
mbImpute empowers DESeq2-phyloseq in identifying DA taxa. **a** The barplots show classification accuracy, measured by 5-fold cross-validated precision-recall area under the curve (PR-AUC), by the random forest algorithm for predicting samples’ disease conditions in two T2D datasets [18, 19] and four CRC datasets [14–17]. The features are the DA taxa detected by DESeq2-phyloseq (light color) or mbImpute-empowered DESeq2-phyloseq (dark color; labeled as mbImpute + DESeq2-phyloseq). **b** The histograms show the distributions of three taxa in control and T2D samples in [18] before and after mbImpute is applied. The three taxa, *Ruminococcus sp_5_1_39BFAA*, *Ruminococcus callidus*, and *Ruminococcus albus*, are identified as enriched in T2D samples only after imputation. **c** The histograms show the distributions of three taxa in control and CRC samples in [17] before and after mbImpute is applied. The three taxa, *Ruminococcus gnavus*, *Lachnospiraceae bacterium_2_1_58FAA*, and *Granulicatella adiacens*, are identified as enriched in CRC samples only after imputation. In **b** and **c**, adjusted *p* values calculated by DESeq2-phyloseq are listed

To investigate whether the DA taxa identified by DESeq2-phyloseq or mbImpute-empowered DESeq2-phyloseq are meaningful disease markers, we evaluate the predictive power of the identified DA taxa for sample disease conditions (control or diseased). For each microbiome dataset, we use the DA taxa, identified by DESeq2-phyloseq or mbImpute-empowered DESeq2-phyloseq, as features and apply the random forest algorithm to predict sample disease conditions. We use the 5-fold cross-validated precision-recall area under the curve (PR-AUC) to evaluate the prediction accuracy (Fig. 3a). We observe that mbImpute-empowered DESeq2-phyloseq leads to overall better prediction accuracy than DESeq2-phyloseq does across the six datasets.

Then, we focus on the Karlsson et al. T2D dataset [18] and the Vogtmann et al. CRC dataset [17], which exhibit the largest improvement in prediction accuracy when the DA taxa identified by mbImpute-empowered DESeq2-phyloseq are used. For the Karlsson et al. T2D dataset [18], we observe that mbImpute-empowered DESeq2-phyloseq outputs a greater number of small *p* values than DESeq2-phyloseq does (Additional file 1: Figure S7), suggesting that more taxa are identified as DA after imputation (in fact, all the DA taxa identified before imputation are still found as DA after imputation). Hence, the improvement in prediction accuracy implies that the DA taxa identified only after imputation contribute to the distinction between control and T2D samples. In particular, we examine three example taxa (*Ruminococcus* species) identified as DA only after imputation. Figure 3b shows the distributions of these three taxa’s abundances (on the log-scale) before and after imputation. For each taxon, we observe that the imputed abundances and the original non-zero abundances have similar ranges and both suggest that the taxon is more abundant in T2D samples than in control samples. However, this abundance difference is obscured by the prevalent zeros before imputation and thus cannot be captured by DESeq2-phyloseq. Literature evidence is consistent with the post-imputation result of the first two taxa. Specifically, the first taxon, *Ruminococcus sp_5_1_39BFAA*, has decreased abundances in T2D patients after the Acarbose treatment [103]. The second taxon, *Ruminococcus callidus*, is shown to be enriched in T2D mouse models [104].

For the Vogtmann et al. CRC dataset [17], the 5-fold cross-validated PR-AUC increases by almost 10% when the DA taxa identified after imputation, instead of those identified before imputation, are used as features. In fact, fewer taxa are identified as DA after imputation (Additional file 1: Figure S8). At the *q* value threshold 0.05, DESeq2-phyloseq identifies 53 DA taxa, while mbImpute-empowered DESeq2-phyloseq identifies 40 DA taxa, with only 17 taxa in overlap. This result suggests that the 23 DA taxa identified only after imputation contribute much to the distinction between control and CRC samples. We examine three of these 23 taxa: *Ruminococcus gnavus*, *Lachnospiraceae bacterium_2_1_58FAA*, and *Granulicatella adiacens*. Figure 3c shows that each taxon has its imputed abundances and its original non-zero abundances in similar ranges; its imputed and original non-zero abundances both suggest it to be more abundant in CRC samples than in control samples. However, this abundance difference is obscured by the prevalent zero abundances before imputation and thus cannot be captured by DESeq2-phyloseq. To confirm the post-imputation result, we find literature evidence for the three taxa. First, several studies have reported that *Ruminococcus gnavus* is associated with a higher risk of CRC [99, 105–107]. Second, two studies have shown that *Lachnospiraceae bacterium_2_1_58FAA* is positively associated with colorectal neoplasms, from which CRC arises [99]. Third, *Granulicatella adiacens* is reported to be associated with CRC progression in both human [83] and mouse studies [108]. We also examine the taxa identified as DA before imputation but not as DA after imputation, and we find that these taxa only differ in zero proportions and have similar non-zero abundance distributions between control and CRC samples (Additional file 1: Figure S10). We argue that such taxa are unlikely to be truly DA because it is questionable whether zero proportion differences are biologically meaningful given the prevalence of technical and sampling zeros. Together, our analysis results on the Karlsson et al. T2D dataset [18] and the Vogtmann et al. CRC dataset [17] suggest that compared to DESeq2-phyloseq, mbImpute-empowered DESeq2-phyloseq can detect DA taxa that are more predictive of sample conditions, and we verify that some DA taxa only detected by mbImpute-empowered DESeq2-phyloseq are functionally relevant by literature evidence.

For all the DA taxa identified by DESeq2-phyloseq and mbImpute-empowered DESeq2-phyloseq in the two T2D and four CRC data datasets, we query the GMrepo database [99] and find two T2D- and one CRC-related functional terms. For each term, we perform the Fisher’s exact test to check its enrichment in the DA taxa identified from the corresponding disease-related datasets. Our results show that all three terms are more enriched in the DA taxa identified after mbImpute is applied (Table 1; Additional files 1, 2, 3, 4, 5, 6 and 7), providing functional support to the efficacy of mbImpute in empowering DESeq2-phyloseq.

**Table 1:** Fisher’s exact test *p* values about the enrichment of T2D- and CRC-related functional terms in the DA taxa found by DESeq2-phyloseq or mbImpute-empowered DESeq2-phyloseq

DA method	T2D term 1*	T2D term 2**	CRC term***
DESeq2-phyloseq	0.54	0.76	0.0027
mbImpute-empowered DESeq2-phyloseq	0.03	0.17	0.0010

For each term, the DA taxa identified by each method from the corresponding datasets are pooled to do the test.

^*^T2D term 1: “The time period before the development of symptomatic diabetes. For example, certain risk factors can be observed in subjects who subsequently develop INSULIN RESISTANCE as in type 2 diabetes (DIABETES MELLITUS, TYPE 2).”

^**^T2D term 2: “A cluster of symptoms that are risk factors for CARDIOVASCULAR DISEASES and TYPE 2 DIABETES MELLITUS. The major components of metabolic syndrome include ABDOMINAL OBESITY; atherogenic DYSLIPIDEMIA; HYPERTENSION; HYPERGLYCEMIA; INSULIN RESISTANCE; a proinflammatory state; and a prothrombotic (THROMBOSIS) state.”

^***^CRC term: “Tumors or cancer of the COLON or the RECTUM or both. Risk factors for colorectal cancer include chronic ULCERATIVE COLITIS; FAMILIAL POLYPOSIS COLI; exposure to ASBESTOS; and irradiation of the CERVIX UTERI”

Furthermore, we analyze the overlap of the DA taxa identified in the two T2D datasets [18, 19]. There is no overlap in the two sets of DA taxa identified by DESeq2-phyloseq, but *Clostridium bolteae* is identified by mbImpute-empowered DESeq2-phyloseq in both datasets. In fact, *Clostridium bolteae* has been reported as enriched in T2D samples in the Qin et al. dataset [19] but not in the Karlsson et al. dataset [18] In our analysis on the Karlsson et al. T2D dataset [18], *Clostridium bolteae* has FDR-adjusted *p* values 0.347 and 0.036 before and after imputation, respectively (abundance distributions in Additional file 1: Figure S11). Literature evidence suggests that *Clostridium bolteae* is positively associated with T2D in both human [109] and mouse studies [110].

For the four CRC datasets [14–17], we analyze the DA taxa identified in at least two datasets before and after imputation. Specifically, DESeq2-phyloseq and mbImpute-empowered DESeq2-phyloseq respectively identify four and 18 taxa (with three taxa in overlap) that have significantly lower abundances in CRC samples than in normal samples. Among these taxa, DESeq2-phyloseq only identifies *Bifidobacterium animalis*, while mbImpute-empowered DESeq2-phyloseq additionally identifies three other *Bifidobacterium* species: *Bifidobacterium bifidum*, *Bifidobacterium catenulatum*, and *Bifidobacterium longum*. Additional file 1: Figures S12–14 show the distributions of these three taxa’s abundances (on the log-scale) before and after imputation. Literature evidence indicates that *Bifidobacterium* is beneficial to the immune system against CRC [111–113] and has been used as probiotics [114]; all the four *Bifidobacterium* species detected by mbImpute-empowered DESeq2-phyloseq have been reported to have significantly lower abundances in CRC samples [115, 116]. Together, our overlap analysis on T2D and CRC datasets suggests that mbImpute helps recover the DA taxa that are detected in one dataset but missed in another due to prevalent zeros.

### mbImpute preserves distributional characteristics of taxa’s non-zero abundances and recovers downsampling zeros

**Fig. 4:**
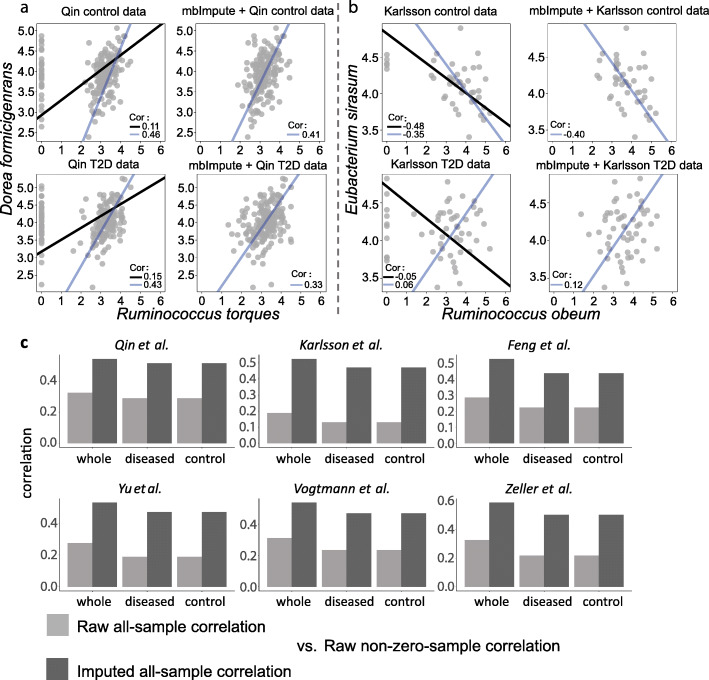
mbImpute preserves distributional characteristics of taxa’s non-zero abundances. **a** Top: two scatter plots show the relationship between the abundances of *Dorea formicigenerans* and *Ruminococcus torques* in Qin et al.’s control samples [19], with or without using mbImpute as a preceding step. The left plot shows two standard major axis (SMA) regression lines and two corresponding Pearson correlations based on the raw data (black: based on all the samples; blue: based on only the samples where both taxa have non-zero abundances). The right plot shows the SMA regression line (blue) and the Pearson correlation using all the samples in the imputed data. Bottom: two scatter plots for the same two taxa in Qin et al.’s T2D samples [19], with lines and legends defined the same as in the top panel. **b** Four scatter plots show the SMA regression lines and correlations between *Eubacterium sirasum* and *Ruminococcus obeum* in Karlsson et al.’s control and T2D samples [18], with lines and legends defined the same as in **a**. **c** Each bar shows the Pearson correlation between taxon-taxon correlations in raw data (light gray) or imputed data (dark gray) using all samples and taxon-taxon correlations in raw data using non-zero samples only. The two correlations are calculated for two T2D datasets and four CRC datasets using diseased samples, control samples, and whole data

In the DA analysis described in the last section, we observe that mbImpute can well maintain the distributions of taxa’s non-zero abundances, see Fig. 3b, c. To further verify the property of mbImpute in preserving characteristics of non-zero abundances, we examine pairwise taxon-taxon relationships in the two T2D WGS datasets: Karlsson et al. and Qin et al. datasets [18, 19]. For a pair of taxa, we calculate two Pearson correlations based on the raw data on the log-scale: one using all the samples (“raw all-sample correlation”) and the other using only the samples where both taxa have non-zero abundances (“raw non-zero-sample correlation”). In this section, we perform our analysis on the log-scale of the taxa count matrix since one of the assumptions for Pearson correlation is the normality of both variables, and microbiome count data on the log-scale better resemble a continuous normal distribution. For the same pair of taxa, we also calculate a Pearson correlation based on the imputed data by mbImpute on the log-scale, using all the samples (“imputed all-sample correlation”). As shown in Fig. 4a, b, there are vast differences between the raw all-sample correlations and the corresponding raw non-zero-sample correlations. However, the imputed all-sample correlations better resemble the corresponding raw non-zero-sample correlations, suggesting that mbImpute well preserves pairwise taxon-taxon correlations encoded in taxa’s non-zero abundances.

We also explore the linear relationship of each taxon pair using the standard major axis (SMA) regression, which, unlike the least-squares regression, treats two taxa symmetrically. For a pair of taxa, we perform two SMA regressions on the raw data: one using all the samples (“raw all-sample regression”) and the other using only the samples where both taxa have non-zero abundances (“raw non-zero-sample regression”). We also perform the SMA regression on the imputed data by mbImpute, using all the samples (“imputed all-sample regression”). Figure 4a, b show that the raw all-sample regressions and the raw non-zero-sample regressions return vastly different lines. Especially, the two lines between *Eubacterium sirasum* and *Ruminococcus obeum* in the Karlsson et al. T2D dataset [18] (Fig. 4b bottom left) have slopes with opposite signs. In contrast, the imputed all-sample regressions output lines with slopes similar to those of the raw non-zero-sample regressions. This result again confirms mbImpute’s capacity for preserving characteristics of taxa’s non-zero abundances in microbiome data.

Furthermore, we systematically evaluate the performance of mbImpute in preserving raw non-zero-sample correlations on the two T2D WGS datasets and the four CRC WGS datasets, with each dataset containing samples in two groups: diseased and control. Figure 4c show that the imputed all-sample correlations resemble the raw non-zero-sample correlations much better than the raw all-sample correlations do, on every dataset including all samples (“whole” in Fig. 4c). Moreover, within each sample group in each dataset (“diseased” and “control” in Fig. 4c), the imputed all-sample correlations still better resemble the raw non-zero-sample correlations than the raw all-sample correlations do. Note that the resemblance is defined based on the Pearson correlation of two sets of correlations. Additional file 1: Figure S15 shows that the same conclusion holds when the resemblance is defined based on the Spearman correlation. Note that mbImpute does not use the group information of each sample in its imputation process.

Our results echo existing concerns about spurious taxon-taxon correlations in microbiome data due to excess non-biological zeros [117, 118]. In other words, taxon-taxon correlations cannot be accurately estimated from raw data using all samples. Without imputation, an intuitive approach is to use taxa’s non-zero abundances to estimate taxon-taxon correlations; however, this approach reduces the sample size for estimating each taxon pair’s correlation because it does not use the samples containing zero abundances for either taxon, and it also makes different taxon pairs’ correlation estimates rely on different samples. To address these issues, mbImpute provides another approach: its imputed data allow taxon-taxon correlations to be estimated from all samples. Moreover, we observe that mbImpute makes log-transformed taxon abundances closer to be normally distributed (Additional file 1: Figure S16); thus, the Pearson correlation is a more meaningful measure for taxon-taxon associations on the imputed data than on the raw data.

In addition, based on the T2D WGS dataset generated by Qin et al. [19], we verify mbImpute’s capacity to identify non-biological zeros generated by downsampling. In each sample (i.e., each row in the sample-by-taxon count matrix), we assign every taxon a sampling probability proportional to its count, i.e., the larger the count, the more likely the taxon is to be sampled; based on these probabilities, we sample 60% or 30% of the non-zero taxon counts, and we set the unsampled counts to zeros (corresponding to a removal rate of 40% or 70%); we repeat the downsampling independently for ten times. After applying mbImpute to the downsampled count matrices, we find that mbImpute correctly identifies 95.83*%* and 92.83*%* (on average) of the newly introduced non-biological zeros under the two removal rates. Before imputation, the average Pearson correlations between the downsampled matrices and the original matrix (on the log-scale) are 0.76 and 0.53 under the two removal rates. After applying mbImpute to all the three matrices, the correlations are increased to 0.87 and 0.76 (Table 2). This result confirms the effectiveness of mbImpute in recovering zeros due to downsampling.

**Table 2:** Effectiveness of mbImpute in identifying zeros due to downsampling of Qin et al.’s T2D WGS dataset [19]. For each of two removal rates 40% and 70%, we repeat independent downsampling for ten times

Removal rate	40%	70%
% of downsampling zeros identified	95.83*%*±0.46*%*	92.83*%*±0.92*%*
Pearson correlation before imputation	0.7565±0.0023	0.5261±0.0016
Pearson correlation after imputation	0.8747±0.0100	0.7582±0.0235

For each removal rate (column), the first row lists the average percentage of downsampling zeros identified by mbImpute; the second row lists the average Pearson correlation between a downsampled matrix and the original matrix (on the log-scale) before imputation; the third row lists the average Pearson correlation (on the log-scale) after mbImpute is used. Each average calculated across the ten downsampling and is accompanied with an error margin, i.e., 1.96 times the standard error over the ten downsampling

### mbImpute increases the similarity of microbial community structure between 16S rRNA and WGS data

**Fig. 5:**
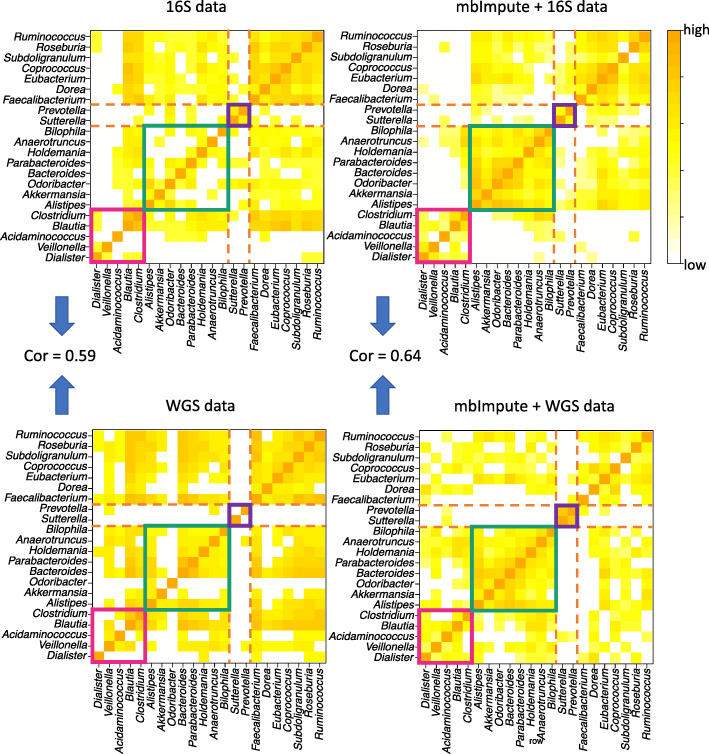
mbImpute improves the similarity of taxon-taxon correlations between 16S and WGS data of microbiomes in healthy human stool samples. Four Pearson correlation matrices are calculated based on a common set of genus-level taxa’s abundances in 16S and WGS data, with or without using mbImpute as a preceding step. Before imputation, the Pearson correlation between the two correlation matrices is 0.59, and this correlation increases to 0.64 after imputation. For illustration purposes, each heatmap shows square roots of Pearson correlations, with the bottom 40% of values truncated to 0. The magenta, green, and purple squares highlight three taxon groups, each of which contains strongly correlated taxa and is consistent between the 16S and WGS data after imputation

We further show that mbImpute can enhance the similarity of taxon-taxon correlations inferred from microbiome data measured by two technologies—16S rRNA sequencing and WGS. We use two microbiome datasets of healthy human stool samples: a 16S rRNA dataset from the Human Microbiome Project [119] and a WGS dataset from the control samples in Qin et al. [19] We compare the genus-level taxon-taxon correlations between these two datasets, and we perform the comparison again after applying mbImpute. Figure 5 shows that mbImpute increases the similarity between the taxon correlation structures in the two datasets. Before imputation, the Pearson correlation between the two correlation matrices (one computed from 16S rRNA taxon abundances and the other from WGS taxon abundances) is 0.59; mbImpute increases the correlation to 0.64. In particular, we observe three taxon groups (highlighted by magenta, green, and purple squares in Fig. 5) supported by both 16S rRNA and WGS data after imputation. Notably, in the magenta squares, *Acidaminococcus* has correlations with *Dialister* and *Blautia* only after imputation, and this result is consistent with the literature: *Acidaminococcus* and *Dialister* are both reported to have low abundances in healthy human stool samples [120]; *Acidaminococcus* and *Blautia* are both associated with risks of T2D and obesity, lipid profiles, and homeostatic model assessment of insulin resistance [121]. The green squares contain three bile-tolerant genera: *Alistipes*, *Bilophila*, and *Bacteroides* [122]. The raw 16S and WGS data only reveal the correlation between *Bacteroides* and *Alistipes*, but mbImpute recovers the correlations *Bilophila* has with *Alistipes* and *Bacteroides*. The purple squares indicate a strong correlation between *Sutterella* and *Prevotella* after imputation, yet this correlation is not observed in raw WGS data. We verify this correlation in the MACADAM database [123], which contains metabolic pathways associated with microbes. Out of 1260 pathways, *Sutterella* and *Prevotella* are associated with 154 and 278 pathways, respectively, and 122 pathways are in common; Fisher’s exact test finds that the overlap is statistically significant (*p* value <2.2×10^−16^), suggesting that *Sutterella* and *Prevotella* may be functionally related. Overall, our results indicate that mbImpute can facilitate meta-analysis of 16S and WGS data by alleviating the hurdle of prevalent non-biological zeros.

We perform a negative control study to confirm that the increased similarity between 16S rRNA and WGS data is not an artifact introduced by mbImpute. We use a 16S rRNA dataset of human oral samples and a WGS dataset of human stool samples, which are expected to have different genus-level taxon-taxon correlations. Same as in the previous study, we compare the genus-level taxon-taxon correlations between the two datasets before and after applying mbImpute. Additional file 1: Figure S17 shows that mbImpute decreases the similarity between the taxon correlation matrices of the two datasets. Before imputation, the Pearson correlation between the two correlation matrices is 0.21; mbImpute decreases the correlation to 0.09.

## Discussion

A critical challenge in microbiome data analysis is statistical inference of taxon abundance from highly sparse and noisy data. Our proposed method, mbImpute, will address this challenge and facilitate analysis of both 16S and WGS data; mbImpute works by correcting non-biological zeros and retaining taxa’s non-zero abundance distributions after imputation. As the first imputation method designed for microbiome data, mbImpute is shown to outperform multiple state-of-the-art imputation methods developed for other data types. In the DA analysis, we show that mbImpute-empowered DESeq2-phyloseq has better performance in selecting predictive taxa for disease conditions comparing to DESeq2-phyloseq. The reason is that mbImpute-empowered DESeq2-phyloseq is able to identify the taxa missed by the DESeq2-phyloseq (due to excess zeros) but should be called DA (i.e., having non-zero abundances that exhibit significantly different means between two sample groups). We then demonstrate that mbImpute preserves taxa’s non-zero abundance distributions. As a result, taxon-taxon correlations calculated from all samples after imputation better resemble the taxon-taxon correlations calculated from non-zero counts only. Hence, mbImpute can facilitate taxon network analysis by allowing all taxon pairs to have meaningful correlations computed from all samples. Moreover, mbImpute improves the reproducibility of DA taxon identification across studies and the consistency of microbial community detection between 16S and WGS data.

In the application of mbImpute, two practical concerns are what normalization method and phylogenetic distance metric work the best with mbImpute. First, the goal of normalization is to make taxon counts comparable across samples, a necessary assumption of mbImpute. In our results, we think our way of normalization is sufficient to meet this assumption. However, the appropriate normalization method for mbImpute is case by case in future applications, depending on whether confounders such as batch effects are observable; hence, users’ judgment is indispensable. We recognize that benchmarking normalization methods for microbiome data is a separate project. Hence, we refer users to benchmark papers [20, 124] to guide their choice of benchmark methods. Second, users may specify the phylogenetic distances between taxa based on their domain knowledge. In our results, we define the phylogenetic distance between two taxa as the number of branches connecting them in a phylogenetic tree, but alternative choices exist, such as the total lengths of the branches. If users want to choose a distance metric, we recommend that they supply the phylogenetic distances defined by candidate metrics into mbImpute and choose the metric that leads to the smallest cross-validated MSE, i.e., the cross-validated imputation error of mbImpute on non-missing data.

Regarding the mbImpute-empowered DA analysis, we note that it offers a new perspective of identifying DA taxa from microbiome 16S and WGS data after imputation. We have summarized three statistical definitions of DA taxa in microbiome data in Additional file 1: Statistical definitions of DA taxa. Note that mbImpute-empowered DA analysis is advantageous in that it alleviates the existence of non-biological zeros, and it uses all available samples for DA testing. A controversial question is, if a taxon has few zeros in condition 1 but few non-zeros in condition 2, and the non-zero values have similar magnitudes in the two conditions, whether or not should this taxon be called DA. We note that mbImpute is unlikely to impute the predominant zeros in condition 2 because it would treat these zeros as biological zeros. Hence, mbImpute-empowered DA analysis is likely to call such a taxon as DA.

There has been a long-standing concern about sample contamination in microbiome sequencing data, e.g., contamination from DNA extraction kits and laboratory reagents [125, 126]. Existing studies have attempted to address this issue via calibrated sequencing operations [126–128] and computational methods [129, 130]. We recommend researchers to perform contamination removal before applying mbImpute. Moreover, by its design, mbImpute is robust to certain types of sample contamination that result in outlier taxa and samples. For each outlier taxon, mbImpute would borrow little information from other taxa to impute this outlier taxon’s abundances. Similarly, mbImpute is robust to the existence of outlier samples that do not resemble any other sample.

In statistical inference, a popular and powerful technique is the use of indirect evidence by borrowing information from other observations, as seen in regression, shrinkage estimation, empirical Bayes, among many others [131]. Imputation follows the indirect evidence principle, where the most critical issue is to decide what observations to borrow information from so as to improve data quality instead of introducing excess biases. To achieve this, mbImpute employs penalized regression to selectively leverage similar samples, similar taxa, and sample covariates to impute likely non-biological zeros, whose identification also follows the indirect evidence principle by incorporating sample covariates into consideration. Also, mbImpute provides a flexible framework to make use of microbiome metadata: it selectively borrows metadata information when available, but it does not rely on the existence of metadata (see Methods).

In the comparison of mbImpute with softImpute, a general matrix imputation method widely used in other fields, we observe that softImpute’s imputed taxon abundances exhibit artificial spikes and smaller variances than those of the original non-zero abundances, possibly due to its low-rank assumption. In contrast, mbImpute is a regression-based method that focuses more on local matrix structures, and we find that it retains well the original non-zero abundance distributions. We will investigate the methodological differences between mbImpute and softImpute in a future study.

Moreover, we observe that, similar to each taxon’s non-zero abundances, the imputed abundances exhibit a bell-shaped distribution across samples on the log-scale. This suggests that statistical methods utilizing normal distributional assumptions become suitable and applicable to imputed taxon abundances. A possible use of imputed microbiome data is to construct a taxon-taxon interaction network, to which network analysis methods may be applied to find taxon modules and hub taxa [132]. As a preliminary exploration, we construct Bayesian networks of taxa based on the two T2D datasets [18, 19] after applying mbImpute. Interesting changes are observed in taxon interactions from control samples to T2D samples (Additional file 1: Figures S18–19). For example, two genera, *Ruminococcus* and *Eubacterium*, have interactive species in control samples but not in T2D samples. In future research, differential network analysis methods can be applied to find taxon communities that differ between two sample groups.

## Methods

### mbImpute methodology

Here, we describe mbImpute, a statistical method that corrects prevalent non-biological zeros in microbiome data. As an overview, mbImpute takes a taxon count matrix as input; pre-processes the data; identifies the likely non-biological zeros and imputes them based on the input count matrix, sample covariates, and taxon phylogeny; and outputs an imputed count matrix.

#### Notations

We denote the sample-by-taxon taxa count matrix as $\mathbf {M} = (M_{{ij}}) \in \mathbb {Z}_{\ge 0}^{n \times m}$, where *n* is the number of microbiome samples and *m* is the number of taxa. We denote the sample covariate matrix (i.e., metadata) as **X**∈*ℝ*^*n*×*q*^, where *q* equals the number of covariates plus one (for the intercept). (By default, mbImpute includes sample library size as a covariate.) In addition, we define a phylogenetic distance matrix of taxa as $\phantom {\dot {i}\!}\mathbf {D} = (D_{jj^{\prime }}) \in \mathbb {Z}_{\ge 0}^{m \times m}$, where $\phantom {\dot {i}\!}D_{jj^{\prime }}$ represents the number of branches connecting taxa *j* and *j*^′^ in the phylogenetic tree or user-specified distance between taxa *j* and *j*^′^.

#### Data pre-processing

mbImpute requires every taxon’s counts across samples to be on the same scale before imputation. If this condition is unmet, normalization is needed. However, how to properly normalize microbiome data is challenging, and multiple normalization methods have been developed in recent years [29, 133, 134]. Regarding the choice of an appropriate normalization method, users may refer to benchmark papers [20, 124]. To give users the flexibility of choosing an appropriate normalization method, mbImpute allows users to input a normalized count matrix by specifying that the input matrix does not need normalization. Otherwise, mbImpute normalizes samples by library size.

*Default normalization (optional)* To account for the varying library sizes (i.e., total counts) of samples, mbImpute first normalizes the count matrix **M** by row. The normalized count matrix is denoted as $\mathbf {M}^{(\mathcal {N})} = \left (M^{(\mathcal {N})}_{{ij}}\right) \in \mathbb {R}_{\ge 0}^{n \times m}$, where

$$ M^{(\mathcal{N})}_{{ij}} = 10^{6} \cdot \frac{ M_{{ij}}}{\sum_{j^{\prime} = 1}^{m} M_{ij^{\prime}}}. $$

After this normalization, every sample has a total count of 10^6^.

mbImpute applies the logarithmic transformation to the normalized counts so as to reduce the effects of extremely large counts [82]. The resulted log-transformed normalized matrix is denoted as $\mathbf {Y} = (Y_{{ij}}) \in \mathbb R_{> 0}^{n \times m}$, with

$$Y_{{ij}} = \log_{10}{\left(M_{{ij}}^{(\mathcal{N})} + 1.01 \right)},  $$

where the value 1.01 is added to make *Y*_*ij*_>0 to avoid the occurrence of infinite values in a later parameter estimation step, following [50, 81]. This logarithmic transformation allows us to fit a continuous probability distribution to the transformed data, thus simplifying the statistical modeling. In the following text, we refer to **Y** as the sample-by-taxon abundance matrix.

#### mbImpute step 1: identification of taxon abundances that need imputation

mbImpute assumes that each taxon’s abundances, i.e., a column in **Y**, follow a mixture model. The model consists of two components: a Gamma distribution for the taxon’s likely non-biological zeros and low abundances and a normal distribution for the taxon’s actual abundances, with the normal mean incorporating sample covariate information (including sample library size as a covariate). Specifically, mbImpute assumes that the abundance of taxon *j* in sample *i*, *Y*_*ij*_, follows the following mixture distribution:

$$ Y_{{ij}} \sim p_{j} \cdot \Gamma \left(\alpha_{j}, \beta_{j} \right) + (1-p_{j}) \cdot \mathcal{N} \left(X_{i \cdot}^{\mathsf{T}} \gamma_{j}, \sigma_{j}^{2} \right), $$

where *p*_*j*_∈(0,1) is the missing rate of taxon *j*, i.e., the probability that taxon *j* is falsely undetected, *Γ*(*α*_*j*_,*β*_*j*_) denotes the Gamma distribution with shape parameter *α*_*j*_>0 and rate parameter *β*_*j*_>0, and $\mathcal {N} \left (X_{i \cdot }^{\mathsf {T}} \gamma _{j}, \sigma _{j}^{2} \right)$ denotes the normal distribution with mean $X_{i \cdot }^{\mathsf {T}} \gamma _{j}$ and standard deviation *σ*_*j*_>0. In other words, with probability *p*_*j*_,*Y*_*ij*_ is a missing value that needs imputation; with probability 1−*p*_*j*_,*Y*_*ij*_ is sampled from the non-missing abundance distribution of taxon *j* and does not need imputation. mbImpute models the normal mean parameter as a linear function of sample covariates: $X_{i \cdot }^{\mathsf {T}} \gamma _{j}$, where $X_{i \cdot } \in \mathbb {R}^{q}$ denotes the *i*th row in the covariate matrix **X**, i.e., the covariates of sample *i*, and $\gamma _{j} \in \mathbb {R}^{q}$ represents the *q* covariates’ effects (including the intercept) on taxon *j*’s abundance. This formulation allows a taxon to have similar expected abundances (when not missing) in samples with similar covariates.

The intuition behind this model is that taxon *j*’s non-missing abundance in a sample is drawn from a normal distribution, whose mean depicts the expected abundance given the sample covariates. However, due to library preparation and under-sampling issues in sequencing, false zero or low counts could have been introduced into the data, creating another mode near zero in taxon *j*’s abundance distribution. mbImpute models that mode using a Gamma distribution with mean *α*_*j*_/*β*_*j*_, which is close to zero.

mbImpute fits this mixture model to taxon *j*’s abundances using the expectation-maximization (EM) algorithm to obtain the maximum likelihood estimates $\hat {p}_{j}, \hat {\alpha }_{j}, \hat {\beta }_{j}, \hat {\gamma }_{j}$, and $\hat {\sigma }_{j}^{2}$. Additional file 1: Figure S20 shows four examples where the fitted mixture model well captures the bimodality of an individual taxon’s abundance distribution. However, some taxa are observed to have an abundance distribution containing a single mode that can be well modeled by a normal distribution. When that occurs, the EM algorithm would encounter a convergence issue. To fix this, mbImpute uses a likelihood ratio test (LRT) to first decide if the Gamma-normal mixture model fits to taxon *j*’s abundances significantly better than a normal distribution $Y_{{ij}} \sim \mathcal {N} \left (X_{i \cdot }^{\mathsf {T}} \eta _{j}, \omega _{j}^{2} \right)$ does. Given the maximum likelihood estimates $\hat {\eta }_{j}$ and $\hat {\omega }_{j}^{2}$ and under the assumption that *Y*_*ij*_’s are all independent, the LRT statistic of taxon *j* is:

$$ \Lambda_{j} = - 2 \ln \frac{\prod_{i = 1}^{n} f_{\mathcal{N}} \left(Y_{{ij}}; X_{i \cdot}^{\mathsf{T}} \hat{\eta}_{j}, \hat{\omega}_{j}^{2} \right)} {\prod_{i = 1}^{n} \left[\hat{p}_{j} \cdot f_{\Gamma} \left(Y_{{ij}}; \hat{\alpha}_{j}, \hat{\beta}_{j} \right) + (1-\hat{p}_{j}) \cdot f_{\mathcal{N}} \left(Y_{{ij}}; X_{i \cdot}^{\mathsf{T}} \hat{\gamma}_{j}, \hat{\sigma}_{j}^{2} \right)\right]}, $$

which asymptotically follows a chi-square distribution with 3 degrees of freedom (because the mixture model has three more parameters than in the normal model) under the null hypothesis that the normal model is the correct model. We summarize the LRT *p* values calculated on six real WGS datasets and observe that few taxa have *p* values greater than 0.05 (see Additional file 1: Figure S21a). Additional file 1: Figure S21b shows the distribution of one randomly picked taxon with LRT *p* value greater than 0.05 in each dataset; these taxa’s log-transformed counts do not have a mode close to zero. If the LRT *p* value ≤0.05, mbImpute uses the mixture model to decide which abundances of taxon *j* need imputation. Specifically, mbImpute decides if *Y*_*ij*_ needs imputation based on the estimated posterior probability that *Y*_*ij*_ comes from the Gamma component:

$$ d_{{ij}} = \frac{\hat{p}_{j} \cdot f_{\Gamma} \left(Y_{{ij}}; \hat{\alpha}_{j}, \hat{\beta}_{j} \right)}{\hat{p}_{j} \cdot f_{\Gamma} \left(Y_{{ij}}; \hat{\alpha}_{j}, \hat{\beta}_{j} \right) + (1-\hat{p}_{j}) \cdot f_{\mathcal{N}} \left(Y_{{ij}}; X_{i \cdot}^{\mathsf{T}} \hat{\gamma}_{j}, \hat{\sigma}_{j}^{2} \right) }, $$

where $f_{\Gamma }(\cdot ; \hat \alpha _{j}, \hat \beta _j)$ and $f_{\mathcal {N}}(\cdot ; X_{i \cdot }^{\mathsf {T}} \hat {\gamma }_{j}, \hat {\sigma }_{j}^{2})$ represent the probability density functions of the estimated Gamma and normal components in the mixture model. Otherwise, if the LRT *p*-value >0.05, mbImpute concludes that none of taxon *j*’s abundances need imputation and sets *d*_1*j*_=⋯=*d*_*nj*_=0.

Based on the *d*_*ij*_’s, mbImpute defines a set *Ω* of (sample, taxon) pairs whose abundances are unlikely missing and thus do not need imputation:

$$ \Omega = \left\{(i,j): d_{{ij}} < d_{\text{thre}}, i=1,\ldots,n; j=1,\ldots,m\right\},  $$

and a complement set *Ω*^*c*^ containing other (sample, taxon) pairs whose abundances need imputation:

$$\Omega^{c} = \left\{(i,j): d_{{ij}} \geq d_{\text{thre}}, i=1,\ldots,n; j=1,\ldots,m\right\}.  $$

Although *d*_thre_=0.5 is used as the default threshold on *d*_*ij*_’s to decide the abundances that need imputation, mbImpute is fairly robust to this threshold choice because most *d*_*ij*_’s are concentrated around 0 or 1. We show this phenomenon in Additional file 1: Figure S22, which displays the distribution of all the *d*_*ij*_’s in the data from [14–19].

To summarize, mbImpute does not impute all zeros in the taxon count matrix; instead, it first identifies the abundances that are likely missing using a mixture-modelling approach, and it then only imputes these values in the next step.

#### mbImpute step 2: imputation of the missing taxon abundances

In step 1, mbImpute identifies a set *Ω* of the (sample, taxon) pairs whose abundances do not need imputation. To impute the abundances in *Ω*^*c*^, mbImpute first learns inter-sample and inter-taxon relationships from *Ω* by training a predictive model for *Y*_*ij*_, the abundance of taxon *j* in sample *i*. The rationale is that taxon *j* should have similar abundances in similar samples, and that in every sample, the taxa similar to taxon *j* should have abundances similar to taxon *j*’s abundance. In addition, sample covariates are assumed to carry predictive information of taxon abundances. Hence, for interpretability and stability reasons, mbImpute uses a linear model to combine the predictive power of similar taxa, similar samples, and sample covariates:

$$Y_{{ij}} = Y_{i\cdot}^{\mathsf{T}} \kappa_{j} + Y_{\cdot j}^{\mathsf{T}} \tau_{i} + X_{i \cdot}^{\mathsf{T}} \zeta_{j} + \epsilon_{{ij}},  $$

where $Y_{i \cdot } \in \mathbb {R}_{>0}^{m}$ denotes the *m* taxa’s abundances in sample *i*, $Y_{\cdot j} \in \mathbb {R}_{>0}^{n}$ denotes taxon *j*’s abundances in the *n* samples, $X_{i \cdot } \in \mathbb {R}^{q}$ denotes sample *i*’s covariates (including the intercept), and *ε*_*ij*_ is the error term. The parameters to be estimated include $\kappa _{j} \in \mathbb {R}^{m}, \tau _{i} \in \mathbb {R}^{n}$ and $\zeta _{j} \in \mathbb {R}^{q}, i=1,\ldots,n$; *j*=1,…,*m*. Note that *κ*_*j*_ represents the *m* taxa’s coefficients (i.e., weights) for predicting taxon *j*, with the *j*th entry set to zero, so that taxon *j* would not predict itself; *τ*_*i*_ represents the *n* samples’ coefficients (i.e., weights) for predicting sample *i*, with the *i*th entry set to zero, so that sample *i* would not predict itself; *ζ*_*j*_ represents the coefficients of sample covariates for predicting taxon *j*. In the model, the first term $Y_{i\cdot }^{\mathsf {T}} \kappa _{j}$ borrows information across taxa, the second term $Y_{\cdot j}^{\mathsf {T}} \tau _{i}$ borrows information across samples, and the third term $X_{i \cdot }^{\mathsf {T}} \zeta _{j}$ borrows information from sample covariates. The total number of unknown parameters is *m*(*m*−1)+*n*(*n*−1)+*mq*, while our data **Y** and **X** together have *nm*+*nq* values only. Given that often *m*≫*n*, the parameter estimation problem is high dimensional, as the number of parameters far exceeds the number of data points. mbImpute performs regularized parameter estimation by using the Lasso-type *ℓ*_1_ penalty, which leads to good prediction and simultaneously selects predictors (i.e., similar samples and similar taxa) to ease interpretation. That is, mbImpute estimates the above parameters by minimizing the following loss function:

$$\begin{array}{*{20}l} L\left(\left\{\kappa_{j}, \zeta_{j}\right\}_{j=1}^{m}, \{\tau_{i}\}_{i=1}^{n}\right) &:= \sum_{(i,j) \in \Omega} \left[ Y_{{ij}} - \left(Y_{i\cdot}^{\mathsf{T}} \kappa_{j} + Y_{\cdot j}^{\mathsf{T}} \tau_{i} + X_{i \cdot}^{\mathsf{T}} \zeta_{j} \right) \right]^{2}\\ &\quad+ \lambda \left(\sum_{j = 1}^{m} \sum_{j^{\prime} \neq j}^{m} D_{jj^{\prime}}^{\psi} |\kappa_{jj^{\prime}}| + \sum_{i = 1}^{n} \sum_{i^{\prime} \neq i}^{n} |\tau_{ii^{\prime}}| \right), \end{array} $$

where *λ*,*ψ*≥0 are tuning parameters chosen by cross-validation, $\phantom {\dot {i}\!}D_{jj^{\prime }}$ represents the phylogenetic distance between taxa *j* and $\phantom {\dot {i}\!}j^{\prime }, \kappa _{jj^{\prime }}$ represents the *j*^′^th element of *κ*_*j*_, and $\tau _{ii^{\prime }}$ represents the *i*^′^th element of *τ*_*i*_. Here $\phantom {\dot {i}\!}D_{jj^{\prime }}^{\psi }$, i.e., $\phantom {\dot {i}\!}D_{jj^{\prime }}$ to the power of *ψ*, represents the penalty weight of $\phantom {\dot {i}\!}|\kappa _{jj^{\prime }}|$ (in our R package implementation, the mbImpute function can take any distance matrix *D* as input that reflects the relationship among taxa specified by the user.) The intuition is that if two taxa are closer in the phylogenetic tree, they are more closely related in evolution and tend to have more similar DNA sequences and biological functions [95, 100], and thus, we want to borrow more information between them. For example, if $\phantom {\dot {i}\!}D_{j_{1} j_{2}} > D_{j_{1} j_{3}}$, i.e., taxa *j*_1_ and *j*_2_ are farther away than taxa *j*_1_ and *j*_3_ in the phylogenetic tree, then the estimate of $\phantom {\dot {i}\!}\kappa _{j_{1} j_{2}}$ is more likely to be shrunk to zero than the estimate of $\phantom {\dot {i}\!}\kappa _{j_{1} j_{3}}$, and mbImpute would use taxon *j*_3_’s abundance more than taxon *j*_2_’s to predict taxon *j*_1_’s abundance. The tuning parameter *ψ* is introduced because the distance $\phantom {\dot {i}\!}D_{jj^{\prime }}$, the number of branches connecting taxa *j* and *j*^′^, may not be the best penalty weight for the prediction purpose. Choosing *ψ* by cross-validation is expected to enhance the predication accuracy.

mbImpute performs the estimation using the R package glmnet [74] and obtains the parameter estimates: $\hat {\kappa }_{j} \in \mathbb {R}^{m}, \hat {\tau }_{i} \in \mathbb {R}^{n}$, and $\hat {\zeta }_{j} \in \mathbb {R}^{q}, i=1,\ldots,n$; *j*=1,…,*m*. Finally, for (*i,j*)∈*Ω*^*c*^, the abundance of taxon *j* in sample *i* is imputed as:

$$ {\hat{Y}}_{{ij}} = Y_{i\cdot}^{\mathsf{T}} \hat{\kappa}_{j} + Y_{\cdot j}^{\mathsf{T}} \hat{\tau}_{i} + X_{i \cdot}^{\mathsf{T}} \hat{\zeta}_{j}, $$

and mbImpute does not alter *Y*_*ij*_ if (*i,j*)∈*Ω*.

Note that mbImpute does not require the availability of the sample covariate matrix **X** or the phylogenetic tree. In the absence of sample covariates, the loss function becomes

$$\begin{array}{*{20}l} L\left(\{\kappa_{j}\}_{j=1}^{m}, \{\tau_{i}\}_{i=1}^{n}\right)\! :=\! \sum_{(i,j) \in \Omega}\! \left(Y_{{ij}} - \left(Y_{i\cdot}^{\mathsf{T}} \kappa_{j} + Y_{\cdot j}^{\mathsf{T}} \tau_{i} \right) \right)^{2} + \lambda \left(\sum_{j = 1}^{m} \sum_{j^{\prime} \neq j}^{m} D_{jj^{\prime}}^{\psi} |\kappa_{jj^{\prime}}| + \sum_{i = 1}^{n} \sum_{i^{\prime} \neq i}^{n} |\tau_{ii^{\prime}}| \right), \end{array} $$

minimizing which returns the parameter estimates: $\hat {\kappa }_{j} \in \mathbb {R}^{m}$ and $\hat {\tau }_{i} \in \mathbb {R}^{n}, i=1,\ldots,n$; *j*=1,…,*m*. Finally, for (*i,j*)∈*Ω*^*c*^, the abundance of taxon *j* in sample *i* is imputed as:

$$ {\hat{Y}}_{{ij}} = Y_{i\cdot}^{\mathsf{T}} \hat{\kappa}_{j} + Y_{\cdot j}^{\mathsf{T}} \hat{\tau}_{i}, $$

and mbImpute does not alter *Y*_*ij*_ if (*i,j*)∈*Ω*. In the absence of the phylogenetic tree, mbImpute sets $\phantom {\dot {i}\!}D_{jj^{\prime }}=1$ for all *j*≠*j*^′^∈{1,…,*m*}.

When *m* is large, mbImpute does not estimate *m*(*m*−1)+*n*(*n*−1)+*mq* parameters but uses the following strategy to increase its computational efficiency. For each taxon *j*, mbImpute selects the *k* taxa closest to it (excluding itself) in phylogenetic distance and sets the other (*m*−*k*) taxa’s coefficients in *κ*_*j*_ to zero. This strategy reduces the number of parameters to *mk*+*n*(*n*−1)+*mq* and decreases the computational complexity from *O*(*m*^2^) to *O*(*m*).

In summary, mbImpute step 2 includes two phases: training on *Ω* and prediction (imputation) on *Ω*^*c*^, as illustrated in Additional file 1: Figure S1.

## Supplementary Information

**Additional file 1** Supplementary materials. It includes simulation settings, analysis details, and supplementary tables and figures.

**Additional file 2** DA taxa identified by DESeq2-phyloseq from the Qin et al. T2D dataset [19] with or without imputation.

**Additional file 3** DA taxa identified by DESeq2-phyloseq from the Karlsson et al. T2D dataset [18] with or without imputation.

**Additional file 4** DA taxa identified by DESeq2-phyloseq from the Feng et al. CRC dataset [15] with or without imputation.

**Additional file 5** DA taxa identified by DESeq2-phyloseq from the Vogtmann et al. CRC dataset [17] with or without imputation.

**Additional file 6** DA taxa identified by DESeq2-phyloseq from the Yu et al. CRC dataset [16] with or without imputation.

**Additional file 7** DA taxa identified by DESeq2-phyloseq from the Zeller et al. CRC dataset [14] with or without imputation.

**Additional file 8** Review history.

## Data Availability

**Imputation methods** We compare mbImpute with five existing imputation methods designed for non-microbiome data: softImpute and four scRNA-seq imputation methods (scImpute, SAVER, MAGIC, and ALRA). All these imputation methods take a count matrix as input and ouput an imputed count matrix with the same dimensions. ***1. softImpute*** We use R package softImpute (version 1.4) and the following command to impute an taxon count matrix (a sample-by-taxon matrix): complete(taxa_count_matrix, softImpute(taxa_count_matrix, rank.max = cv.rankmax)) where rank.max is chosen by 10-fold cross-validation. ***2. scImpute*** We use R package scImpute (version 0.0.9) with the input as a taxon-by-sample count matrix (transpose of the matrix in Fig. 1): scimpute(count_path = ~taxa_count_matrix_trans.csv~, Kcluster = 1, out_dir = ~sim_imp~) where taxa_count_matrix_trans.csv is the input file containing the transposed taxon count matrix. ***3. SAVER*** We use R package SAVER (version 1.1.2) with the input as a taxon-by-sample count matrix (transpose of the matrix in Fig. 1): saver(t(taxa_count_matrix), ncores = 1, estimates.only = TRUE) ***4. MAGIC*** We use Python package MAGIC (version 2.0.3) and the following commands to impute an taxon count matrix: magic_op = magic.MAGIC() magic_op.set_params(n_pca = 40) magic_op.fit_transform(taxa_count_matrix) ***5. ALRA*** We apply R functions normalize_data, choose_k, and alra, which were released on Aug 10, 2019 at https://github.com/KlugerLab/ALRA, and the following commands to impute a taxon count matrix: normalized_mat = normalize_data(taxa_count_matrix) k_chosen = choose_k(normalized_mat, K = 49, noise_start = 44)$k alra(normalized_mat, k = k_chosen)$A_norm_rank_k_cor_sc **DA analysis methods** In simulation studies, we compare five existing DA methods: the Wilcoxon rank-sum test, ANCOM, metagenomeSeq, DESeq2-phyloseq, and Omnibus test. We apply each method to taxon counts, with or without using mbImpute as a preceding step. When mbImpute is used as a preceding step, we call the resulting method a mbImpute-empowered DA method. In real data studies, we compare mbImpute-empowered DESeq2-phyloseq and mbImpute-empowered Omnibus test with DESeq2-phyloseq and Omnibus test, respectively. Each method calculates a *p* value for each taxon and identifies the DA taxa by setting a *p* value threshold to control the FDR. See Additional file 1 for the statistical definitions of DA taxa. ***1. Wilcoxon rank-sum test*** We implement the Wilcoxon rank-sum test using the R function pairwise.wilcox.test in the package stats (version 3.5.1). For each taxon, we perform the test on its counts in two sample groups to obtain a *p* value, which suggests if this taxon is DA between the two groups. In simulations, we use the following command to implement a two-sided test for each taxon: pairwise.wilcox.test(x = taxon_counts, g = condition, p.adjust.method = ~none~) ***2. ANCOM*** We use the ANCOM.main function released on Sep 27, 2019 at https://github.com/FrederickHuangLin/ANCOM[27]. Since this function does not provide an option for a one-sided test, we use its default settings and report its identified DA taxa based on a two-sided test with a significance level 0.05 (sig = 0.05), in both simulations and real data analysis. We note that no external FDR control is implemented. Specifically, we use the following command to obtain the result of ANCOM: ANCOM.main(taxa_count_matrix, covariate_matrix, adjusted = F, repeated = F, main.var = ~condition~, adj.formula = NULL, repeat.var = NULL, multcorr = 2, sig = 0.05, prev.cut = 0.90, longitudinal = F) where taxa_count_matrix is a sample-by-taxon count matrix and covariate_matrix is a sample-by-covariate matrix, same as the input of mbImpute. ***3. metagenomeSeq*** We use two R packages, metagenomeSeq (version 1.28.2) and phyloseq (version 1.30.0). Specifically, we use the following command to obtain the result: mseq_obj <- phyloseq_to_metagenomeSeq(physeq2) pd <- pData(mseq_obj) mod <- model.matrix(∼1 + condition, data = pd) ran_seq <- fitFeatureModel(mseq_obj, mod) where physeq2 is an object created from a count matrix and sample covariates using the phyloseq package. ***4. DESeq2-phyloseq*** We use the DESeq2 (version 1.26.0) package combined with phyloseq (version 1.30.0). Specifically, we use the following command to obtain the result of DESeq2: Deseq2_obj <- phyloseq_to_deseq2(physeq2, ∼ condition) results <- DESeq(Deseq2_obj, test=~Wald~, fitType=~parametric~) where physeq2 is an object created from a count matrix and sample covariates using the phyloseq package. ***5. Omnibus test*** We use the R package mbzinb (version 0.2). Specifically, we use the following command to obtain the result of Omnibus test: mbzinb_data <- mbzinb.dataset(taxa_count_matrix, covariate_matrix) mbzinb_test_result <- mbzinb.test(mbzinb_data, group = ~condition~) For the Wilcoxon rank-sum test, MetagenomeSeq, DESeq2-phyloseq, and Omnibus test, after obtaining the *p* values of all taxa and collecting them into a vector *p*_values, we adjust them for FDR control using the R function p.adjust in the package stats (version 3.5.1): p.adjust(*p*_values, method = ~fdr~) Then, we set the FDR threshold to 0.05 in both simulation and real data analysis. The taxa whose adjusted *p* values do not exceed this threshold are called DA. ANCOM directly outputs the DA taxa. **Classification** We use a 5-fold cross-validated precision-recall area under the curve (PR-AUC) to evaluate the classification results using identified DA taxa as features and diseased/control group as classification labels. We use the R package randomForest (version 4.6-14) to perform the random forest classification and the R package PRROC (version 1.3.1) to calculate the PR-AUC. **T2D and CRC datasets** We apply mbImpute to six real microbiome datasets, each corresponding to an independent study on the relationship between microbiomes and the occurrence of a human disease. All the six datasets were generated by the whole genome shotgun sequencing and are available in the R package curatedMetagenomicData [87]. We compare the disease-enriched DA taxa identified by DESeq2-phyloseq and mbImpute-empowered DESeq2-phyloseq. Below is the description of the six datasets and our analysis. **Two T2D datasets** [18, 19]. The Karlsson et al. dataset [18] contains 145 fecal samples from 70-year-old European women to study the relationship between human gut microbiome compositions and T2D status. The samples/subjects are in three groups: 53 women with T2D, 49 women with impaired glucose tolerance (IGT), and 43 women as the normal control (CON). The eleven sample covariates include the subject’s age, the number of reads in each sample, the triglycerides level, the hba1c level, the ldl (low-density lipoprotein cholesterol) level, the c peptide level, the cholesterol level, the glucose level, the adiponectin level, the hscrp level, and the leptin level. In our analysis, we consider the 147 species-level taxa (having at least 10% non-zero counts in both T2D and CON groups) with phylogenetic information available in the R package curatedMetagenomicData. Qin et al. [19] performed deep shotgun metagenome sequencing on 369 Chinese T2D patients and non-diabetic controls (CON). The two sample covariates include the body mass index, and the number of reads in each sample. We analyze 156 species-level taxa (having at least 10% non-zero counts in both T2D and CON groups) with phylogenetic information. From both datasets, we identify DA taxa by comparing the T2D and CON groups. **Four CRC datasets** [14–17]. Zeller et al. [14] and Feng et al. [15] studied CRC-related microbiomes in three conditions: CRC, small adenoma (ADE; diameter <10 mm), and control (CON). Zeller et al. [14] sequenced the fecal samples of patients across two countries (France and Germany) in these three groups: 191 patients with CRC, 66 patients with ADE, and 42 patients in CON. The sample covariates include the subject’s age category, gender, body mass index and country, and the number of reads in each sample. We include 188 species-level taxa (having at least 10% non-zero counts in both CRC and CON groups) with phylogenetic information. Feng et al. [15] sequenced samples from 154 human subjects aged between 45–86 years old in Australia, including 46 patients with CRC, 47 patients with ADE, and 61 in CON. The sample covariates include the subject’s age category, gender, body mass index, and number of reads in each sample. We include 182 species-level taxa that have at least 10% non-zero counts in both CRC and CON groups. Yu et al. [16] and Vogtmann et al. [17] studied CRC-related microbiomes in two conditions: CRC vs. CON. In detail, [16] sequenced 128 Chinese samples, including 75 patients with CRC and 53 patients in CON. The only sample covariate is the number of reads in each sample. We study 173 species-level taxa that have at least 10% non-zero counts in both CRC and CON groups. Vogtmann et al. [17] included 104 samples from Washington DC and sequenced their fecal samples, including 52 with CRC and 52 in CON. The sample covariates include the subject’s age category, gender, body mass index, and the number of reads in each sample. We include 167 species-level taxa that have at least 10% non-zero counts in both CRC and CON groups. From all the four datasets, we identify DA taxa by comparing the CRC and CON groups. **16S rRNA sequencing datasets** We include two 16S rRNA sequencing datasets from the R package HMP16SData [135] (version 1.6.0). The two datasets correspond to the healthy human stool samples and healthy human oral samples. The healthy stool 16S dataset includes 187 samples and 43140 OTUs, and the healthy oral 16S data includes 190 samples and 43140 OTUs. **Software and code** The mbImputeR package is available at https://github.com/ruochenj/mbImpute [136]. The source code and data for reproducing the results are available at 10.5281/zenodo.4840266 [137]. Both the R package and the source code are under the MIT license.
