# Supplementary material for: mbImpute: an accurate and robust imputation method for microbiome data
Source: Genome Biol. 2021 Jun 28;22:192. doi: 10.1186/s13059-021-02400-4 (PMC8240317; doi:10.1186/s13059-021-02400-4)
Supplement: Supplementary file 1 — Additional file 1 Supplementary materials. It includes simulation settings, analysis details, and supplementary tables and figures. [file 13059_2021_2400_MOESM1_ESM.pdf]

Supplementary for
“mbImpute: an accurate and robust imputation method for microbiome data”

Ruochen Jiang [1], Wei Vivian Li [1,2] and Jingyi Jessica Li [1,3,4,*]

Simulation 1 for benchmarking imputation methods

To compare mbImpute with existing imputation methods developed for non-microbiome data, we generated microbiome abundances from a generative model fitted to the T2D data with 53 subjects and 344 taxa (Karlsson *et al.*, 2013). Below we describe the data generation process step by step.

Complete data generation

1. We followed the data pre-processing steps of mbImpute (see Methods in the main text) to convert the OTU count matrix to log-transformed normalized abundances. Then we removed the taxa with greater than 95% of zero counts (equivalently, $\log_{10}(1.01)$ abundances) across subjects and kept 193 taxa. We denote the abundance matrix after this filtering step by $\mathbf{Y} = (Y_{ij}) \in \mathbb{R}^{n\times m}$, where $n = 53$ and $m = 193$. We also collected the sample covariate matrix $\mathbf{X} \in \mathbb{R}^{n\times q}$, where $q = 12$.
2. Following step 1 of mbImpute (see Methods in the main text), we identified a set Ω of (sample, taxon) pairs whose abundances are unlikely missing and thus do not need imputation.
3. Following step 2 of mbImpute (see Methods in the main text), we fit the following model to the (sample, taxon) pairs in Ω:

$$Y_{ij} = Y_{i\cdot}^{\mathsf{T}}\kappa_j + Y_{\cdot j}^{\mathsf{T}}\tau_i + X_{i\cdot}^{\mathsf{T}}\zeta_j + \epsilon_{ij}\,,$$

[1] Department of Statistics, University of California, Los Angeles, CA 90095-1554
[2] Department of Biostatistics and Epidemiology, Rutgers School of Public Health, Rutgers, New Jersey 08854
[3] Department of Human Genetics, University of California, Los Angeles, CA 90095-7088
[4] Department of Computational Medicine, University of California, Los Angeles, CA 90095-1766
[*] To whom correspondence should be addressed. Email: jli@stat.ucla.edu

by minimizing the loss function

$$\sum_{(i,j)\in\Omega} \left(Y_{ij} - \left(Y_{i\cdot}^{\mathsf{T}}\kappa_j + Y_{\cdot j}^{\mathsf{T}}\tau_i + X_{i\cdot}^{\mathsf{T}}\zeta_j\right)\right)^2 + \lambda\left(\sum_{j=1}^{m}\sum_{j'\neq j}^{m} D_{jj'}^{\psi}|\kappa_{jj'}| + \sum_{i=1}^{n}\sum_{i'\neq i}^{n}|\tau_{ii'}|\right)$$

and obtaining the parameter estimates: $\hat{\kappa}_j \in \mathbb{R}^m$, $\hat{\tau}_i \in \mathbb{R}^n$, and $\hat{\zeta}_j \in \mathbb{R}^q$, $i = 1,\ldots,n$; $j = 1,\ldots,m$. The tuning parameters $\lambda, \psi \geq 0$ were chosen by cross-validation. Note that we constrained κ_j to have only 5 non-zero entries corresponding to the 5 taxa closest to taxon j in phylogenetic distance, i.e., $\{j' : D_{jj'}$ is among the five smallest of all $D_{jr}, r \neq j\}$.

4. We generated the complete, log-transformed abundance of taxon j in sample i, as

$$Y_{ij}^{\text{comp}} = Y_{i\cdot}^{\mathsf{T}}\hat{\kappa}_j + Y_{\cdot j}^{\mathsf{T}}\hat{\tau}_i + X_{i\cdot}^{\mathsf{T}}\hat{\zeta}_j\,.$$

We denote the resulting matrix $\mathbf{Y}^{\text{comp}} = (Y_{ij}^{\text{comp}})$ as the complete data that contain log-transformed abundances without missing values.

Zero-inflated data generation

Next, we introduced zero inflation to $\mathbf{Y}^{\text{comp}}$ and generated $\mathbf{Y}^{\text{zi}}$ by mimicking real data as follows. With the identified Ω, we calculated z_k^{real} (taxon k's proportion of likely false zeros across samples) and μ_k^{real} (taxon k's average abundance after we excluded likely false zeros) for each taxon k in Karlsson *et al.*'s data $\mathbf{Y}$, $k = 1,\ldots,m$. Next we introduced zeros into $\mathbf{Y}^{\text{comp}}$ in the following non-parametric way for each taxon j, $j = 1,\ldots,m$:

1. We calculated taxon j's average abundance in $\mathbf{Y}^{\text{comp}}$ as the mean of column j, denoted by μ_j^{comp};
2. We randomly sampled a value from $\{z_k^{\text{real}} : \mu_k^{\text{real}} \in (\mu_j^{\text{comp}} - 0.5, \mu_j^{\text{comp}} + 0.5)\}$ and denoted it as z_j^{zi}, i.e., the proportion of false zeros to be introduced into taxon j's abundances in $\mathbf{Y}^{\text{comp}}$;
3. We randomly drew false zero indicators $I_{ij} \sim$ Bernoulli(z_j^{zi}) independently for sample $i = 1,\ldots,n$;
4. We set $Y_{ij}^{\text{zi}} = \max\left(Y_{ij}^{\text{comp}} \cdot I_{ij},\, \log_{10}(1.01)\right)$.

The reason why we set the minimum value of $\mathbf{Y}^{\text{zi}}$ to $\log_{10}(1.01)$ is that mbImpute step 1 sets the minimum log-transformed abundance to $\log_{10}(1.01)$ to facilitate the fitting of the Gamma-normal mixture model.

Evaluation criteria for imputation accuracy

After applying an imputation method to $\mathbf{Y}^{\text{zi}}$ (mbImpute also used $\mathbf{X}$), we obtained $\mathbf{Y}^{\text{imp}}$ and evaluated the imputation performance by the following three criteria. Note that mbImpute identified 8 taxa with too many zeros in $\mathbf{Y}^{\text{zi}}$ and excluded them from imputation. For a fair comparison, we

excluded these 8 taxa from the calculation of the evaluation criteria for every imputation method, so m was reduced to $193 - 8 = 185$ in the following.

1. Mean squared error (MSE) between $\mathbf{Y}^{\text{imp}}$ and $\mathbf{Y}^{\text{comp}}$:

$$\text{MSE} = \frac{1}{nm} \sum_{i=1}^{n} \sum_{j=1}^{m} (Y_{ij}^{\text{comp}} - Y_{ij}^{\text{imp}})^2 \,,$$

   which is shown in Fig. 2a.
2. Pearson correlation between $Y_{\cdot j}^{\text{imp}}$ and $Y_{\cdot j}^{\text{comp}}$ for $j = 1, \ldots, m$. The mean of these m correlations is shown in Fig. 2b.
3. Mean and standard deviation (SD) of taxon j in the imputed data vs. those in the complete data:

$$\bar{Y}_{\cdot j}^{\text{imp}} = \frac{1}{n} \sum_{i=1}^{n} Y_{ij}^{\text{imp}} \quad \text{vs.} \quad \bar{Y}_{\cdot j}^{\text{comp}} = \frac{1}{n} \sum_{i=1}^{n} Y_{ij}^{\text{comp}} \,,$$

$$\text{sd}_{\cdot j}^{\text{imp}} = \sqrt{\frac{1}{n-1} \sum_{i=1}^{n} \left(Y_{ij}^{\text{imp}} - \bar{Y}_{\cdot j}^{\text{imp}} \right)^2} \quad \text{vs.} \quad \text{sd}_{\cdot j}^{\text{comp}} = \sqrt{\frac{1}{n-1} \sum_{i=1}^{n} \left(Y_{ij}^{\text{comp}} - \bar{Y}_{\cdot j}^{\text{comp}} \right)^2} \,,$$

   $j = 1, \ldots, m$. In Fig. 2c, we computed the Wasserstein distance between the distribution of $\left\{ \bar{Y}_{\cdot 1}^{\text{imp}} / \text{sd}_{\cdot 1}^{\text{imp}}, \ldots, \bar{Y}_{\cdot m}^{\text{imp}} / \text{sd}_{\cdot m}^{\text{imp}} \right\}$ vs. that of $\{ \bar{Y}_{\cdot 1}^{\text{comp}} / \text{sd}_{\cdot 1}^{\text{comp}}, \ldots, \bar{Y}_{\cdot m}^{\text{comp}} / \text{sd}_{\cdot m}^{\text{comp}} \}$. In Fig. 2d, we computed the Euclidean distance between the imputed data and the complete data as

$$\sqrt{\sum_{j=1}^{m} \left(\bar{Y}_{\cdot j}^{\text{imp}} - \bar{Y}_{\cdot j}^{\text{comp}} \right)^2 + \left(\text{sd}_{\cdot j}^{\text{imp}} - \text{sd}_{\cdot j}^{\text{comp}} \right)^2} \,.$$

Simulation 2 for benchmarking imputation methods based on real WGS data

To further benchmark mbImpute against existing imputation methods developed for non-microbiome data, we used a semi-simulation approach by obtaining a subset of microbiome WGS data with at least 86% non-zeros from a T2D dataset composed of 344 subjects and 469 taxa (Qin *et al.*, 2012). Below we describe the data generation process step by step.

Complete data generation

1. We followed the data pre-processing steps of mbImpute (see Methods in the main text) to convert the OTU count matrix to log-transformed normalized abundances, denoted by $\mathbf{Y} = (Y_{ij}) \in \mathbb{R}^{344 \times 469}$. We also collected the sample covariate matrix $\mathbf{X} \in \mathbb{R}^{344 \times 3}$.
2. Following step 1 of mbImpute (see Methods in the main text), we identified a set Ω of (sample, taxon) pairs whose abundances are unlikely missing and thus do not need imputation.

3. For each taxon, we checked if it has at least 43 non-zero counts, or equivalently, at least 43 abundances greater than $\log_{10}(1.01)$, across the 344 subjects. If yes, we kept the taxon; otherwise, we filtered it out. This step left us with 145 taxa.
4. Denote the index set of samples where taxon j has non-zero counts as

$$\mathcal{I}_j = \{i : Y_{ij} > \log_{10}(1.01),\ i = 1, \ldots, 344\}$$

Note that all $|\mathcal{I}_j| \geq 43$ due the the filtering step. Then we constructed a new index set of samples $\mathcal{I}'_j$, with $|\mathcal{I}'_j| = 50$, in the following way:

   (a) if $|\mathcal{I}_j| < 50$, $\mathcal{I}'_j = \mathcal{I}_j \cup$ a random subset of $\mathcal{I}_j^c$ with size $50 - |\mathcal{I}_j|$;

   (b) otherwise, $\mathcal{I}'_j =$ a random subset of $\mathcal{I}_j$ with size 50.

5. We constructed the complete data by column-stacking taxon j's abundances in the samples in $\mathcal{I}'_j$, with the 50 samples randomly ordered, $j = 1, \ldots, 145$, resulting in $\mathbf{Y}^{\text{comp}} \in \mathbb{R}^{50\times 145}$, which we assumed to have no missing values. By our construction, $\mathbf{Y}^{\text{comp}}$ has at least 86% values greater than $\log_{10}(1.01)$, corresponding to non-zero values on the count scale.
6. We constructed the sample covariate matrix $\mathbf{X}^{\text{comp}} \in \mathbb{R}^{50\times 3}$ as follows: for sample i and covariate r ($i = 1, \ldots, 50$; $r = 1, 2, 3$),

   (a) if covariate r is categorical (e.g., gender), X_{ij}^{comp} was decided by the majority vote of the j-th covariate values of the original samples rearranged into the i-th row of $\mathbf{Y}^{\text{comp}}$: majority($\{X_{i'j}:$ sample i' is in the i-th row of $\mathbf{Y}^{\text{comp}}\}$);

   (b) if covariate r is numerical (e.g., BMI), X_{ij}^{comp} was set to the average of the j-th covariate values of the original samples rearranged into the i-th row of $\mathbf{Y}^{\text{comp}}$: average($\{X_{i'j}:$ sample i' is in the i-th row of $\mathbf{Y}^{\text{comp}}\}$).

Zero-inflated data generation for mimicking WGS data

Next, we introduced zero inflation to $\mathbf{Y}^{\text{comp}}$ and generated $\mathbf{Y}^{\text{zi}}$ by mimicking real WGS data as follows. With the identified Ω, we calculated z_k^{real} (taxon k's proportion of likely false zeros across samples) and μ_k^{real} (taxon k's average abundance after we excluded likely false zeros) for each taxon k in Qin *et al.*'s data, $k = 1, \ldots, 469$. Next we introduced zeros into $\mathbf{Y}^{\text{comp}}$ in the following non-parametric way for each taxon j, $j = 1, \ldots, 145$:

1. We calculated taxon j's average abundance in $\mathbf{Y}^{\text{comp}}$ as the mean of column j, denoted by μ_j^{comp};
2. We randomly sampled a value from $\{z_k^{\text{real}} : \mu_k^{\text{real}} \in (\mu_j^{\text{comp}} - 0.5, \mu_j^{\text{comp}} + 0.5)\}$ and denoted it as z_j^{zi}, i.e., the proportion of false zeros to be introduced into taxon j's abundances in $\mathbf{Y}^{\text{comp}}$;
3. We randomly drew false zero indicators $I_{ij} \sim$ Bernoulli(z_j^{zi}) independently for sample $i = 1, \ldots, 50$;

4. We set $Y_{ij}^{\text{zi}} = \max\left(Y_{ij}^{\text{comp}} \cdot I_{ij}, \log_{10}(1.01)\right)$.

The reason why we set the minimum value of $\mathbf{Y}^{\text{zi}}$ to $\log_{10}(1.01)$ is that mbImpute step 1 sets the minimum log-transformed abundance to $\log_{10}(1.01)$ to facilitate the fitting of the Gamma-normal mixture model.

Evaluation criteria for imputation accuracy

After applying an imputation method to $\mathbf{Y}^{\text{zi}}$ (mbImpute also used $\mathbf{X}^{\text{comp}}$), we obtained $\mathbf{Y}^{\text{imp}}$ and evaluated the imputation performance by the following three criteria, where $n = 50$ and $m = 145$.

1. Mean squared error (MSE) between $\mathbf{Y}^{\text{imp}}$ and $\mathbf{Y}^{\text{comp}}$:

$$\text{MSE} = \frac{1}{nm} \sum_{i=1}^{n} \sum_{j=1}^{m} (Y_{ij}^{\text{comp}} - Y_{ij}^{\text{imp}})^2 \,.$$

2. Pearson correlation between $Y_{\cdot j}^{\text{imp}}$ and $Y_{\cdot j}^{\text{comp}}$ for $j = 1, \ldots, m$. The mean of these m correlations is shown in Fig. 2b.

Simulation 3 for evaluating the accuracy and robustness of mbImpute

To evaluate the accuracy and robustness of mbImpute, we simulated microbiome abundances based on real data (Zeller *et al.*, 2014) using four schemes. We set the number of samples to $n = 50$ and the number of taxa to $m = 200$. Under each scheme, we first generated the complete data, as described below.

Scheme 1: Covariate

1. We randomly sampled (without replacement) 50 samples' six covariates (gender, age, disease subtype, BMI, country, and number of reads) from Zeller *et al.*'s data. Then we added a 50-length vector of ones to the sampled covariates to form a sample covariate matrix $\mathbf{X} \in \mathbb{R}^{n \times q}$, with $q = 7$.
2. We randomly sampled (without replacement) 200 taxa' normalized and log-transformed abundances (see Methods in the main text) from Zeller *et al.*'s data. We fit the Gamma-normal mixture model (step 1 of mbImpute) to each sampled taxon j, and we denote the estimated coefficient vector in the normal mean as $\hat{\gamma}_j \in \mathbb{R}^q$, $j = 1, \ldots, m$.
3. We simulated the log-transformed abundance of taxon j in sample i, $i = 1, \ldots, n$, from the following model:

$$Y_{ij}^{\text{comp}} = X_{i\cdot}^{\mathsf{T}} \hat{\gamma}_j + \epsilon_{ij} \,,$$

where $\epsilon_{ij} \sim \mathcal{N}(0, 1)$ independently. We denote the resulting matrix $\mathbf{Y}^{\text{comp}} = (Y_{ij}^{\text{comp}})$ as the complete data that contain log-transformed abundances without missing values.

Scheme 2: Sample

1. We randomly divided $n = 50$ samples into 5 groups. For each sample i, $i = 1, \ldots, n$, we denote its group index by $k(i) \in \{1, \ldots, 5\}$.
2. We generated a sample-to-sample distance matrix $\mathbf{D}^{\text{samp}} = (D^{\text{samp}}_{ii'})_{n\times n}$:

$$D^{\text{samp}}_{ii'} = \begin{cases} 2(|k(i) - k(i')| + 1) & \text{if } i \neq i' \\ 0 & \text{otherwise} \end{cases}$$

   so that two samples from closer groups (in terms of group index) had a smaller distance.
3. We converted the distance matrix $\mathbf{D}^{\text{samp}}$ into a sample-to-sample covariance matrix $\mathbf{\Sigma}^{\text{samp}}$ such that two samples having a smaller distance would have a larger covariance

$$\Sigma^{\text{samp}}_{ii'} = \begin{cases} \exp\left\{(-D^{\text{samp}}_{ii'}/6)^{1.3}\right\} & \text{if } i \neq i' \\ \exp\left\{(-D^{\text{samp}}_{ii'}/6)^{1.3}\right\} + 1 & \text{otherwise} \end{cases},$$

   where the constants 6 and 1.3 were chosen to make $\mathbf{\Sigma}^{\text{samp}}$ positive definite.
4. We simulated the log-transformed abundances of taxon j in all the n samples, $j = 1, \ldots, m$, from the following model:

$$Y^{\text{comp}}_{\cdot j} \overset{\text{ind}}{\sim} \mathcal{N}(3 \cdot \mathbf{1}_n, \Sigma^{\text{samp}}),$$

   which we collected as columns to form $\mathbf{Y}^{\text{comp}} = (Y^{\text{comp}}_{ij})$, i.e., the complete data containing log-transformed abundances without missing values.

Scheme 3: Taxon

1. We randomly divided $m = 200$ taxa into 10 groups. For each taxon j, $j = 1, \ldots, m$, we denote its group index by $r(j) \in \{1, \ldots, 10\}$.
2. We generated a taxon-to-taxon distance matrix $\mathbf{D}^{\text{taxon}} = (D^{\text{taxon}}_{jj'})_{m\times m}$:

$$D^{\text{taxon}}_{jj'} = \begin{cases} 2(|r(j) - r(j')| + 1) & \text{if } j \neq j' \\ 0 & \text{otherwise} \end{cases}$$

   so that two taxa from closer groups (in terms of group index) had a smaller distance.
3. We converted the distance matrix $\mathbf{D}^{\text{taxon}}$ into a taxa-to-taxa covariance matrix $\mathbf{\Sigma}^{\text{taxon}}$ such that two samples having a smaller distance would have a larger covariance

$$\Sigma^{\text{taxon}}_{jj'} = \begin{cases} \exp\left\{(-D^{\text{taxon}}_{jj'}/12)^{1.3}\right\} & \text{if } j \neq j' \\ \exp\left\{(-D^{\text{taxon}}_{jj'}/12)^{1.3}\right\} + 1 & \text{otherwise} \end{cases},$$

   where the constants 12 and 1.3 were chosen to make $\mathbf{\Sigma}^{\text{taxon}}$ positive definite.

4. We simulated the log-transformed abundances of sample i in all the m taxa, $i = 1, \dots, n$, from the following model:

$$Y_{i\cdot}^{\text{comp}} \overset{\text{ind}}{\sim} \mathcal{N}(3 \cdot \mathbf{1}_m, \mathbf{\Sigma}^{\text{taxon}}) \,,$$

which we collected as rows to form $\mathbf{Y}^{\text{comp}} = (Y_{ij}^{\text{comp}})$, i.e., the complete data containing log-transformed abundances without missing values.

Scheme 4: Taxon + Sample + Covariate

We combined the data generation procedures from the above three schemes. Following the same notations as above, we simulated the log-transformed abundance of taxon j in sample i, $i = 1, \dots, n$, $j = 1, \dots, m$, as follows:

1. We generated baseline values, following scheme 1:

$$Y_{ij}^{\text{comp1}} = X_{i\cdot}^{\mathsf{T}} \hat{\gamma}_j + \epsilon_{ij}$$

to form $\mathbf{Y}^{\text{comp1}} = (Y_{ij}^{\text{comp1}})$.

2. We introduced a sample correlation structure as in scheme 2:

$$Y_{\cdot j}^{\text{comp2}} \overset{\text{ind}}{\sim} \mathcal{N}(Y_{\cdot j}^{\text{comp1}}, \mathbf{\Sigma}^{\text{samp}}) \,,$$

which we collected as columns to form $\mathbf{Y}^{\text{comp2}} = (Y_{ij}^{\text{comp2}})$

3. We introduced a taxon correlation structure as in scheme 3:

$$Y_{i\cdot}^{\text{comp}} \overset{\text{ind}}{\sim} \mathcal{N}(Y_{i\cdot}^{\text{comp2}}, \mathbf{\Sigma}^{\text{taxon}}) \,,$$

which we collected as rows to form $\mathbf{Y}^{\text{comp}} = (Y_{ij}^{\text{comp}})$, i.e., the complete data containing log-transformed abundances without missing values.

Next, we introduced zero inflation to $\mathbf{Y}^{\text{comp}}$ and generated $\mathbf{Y}^{\text{zi}}$ by mimicking real data as follows. We applied the step 1 of mbImpute to Zeller *et al.*'s data, which contain 486 taxa (Zeller *et al.*, 2014); that is, we identified likely false zeros. Then we calculated z_k^{real} (taxon k's proportion of likely false zeros across samples) and μ_k^{real} (taxon k's average abundance after we excluded likely false zeros) for each taxon k in Zeller *et al.*'s data, $k = 1, \dots, 486$. Next we introduced zeros into $\mathbf{Y}^{\text{comp}}$ in the following non-parametric way for each taxon j, $j = 1, \dots, m$:

1. We calculated taxon j's average abundance in $\mathbf{Y}^{\text{comp}}$ as the mean of column j, denoted by μ_j^{comp};
2. We randomly sampled a value from $\{z_k^{\text{real}} : \mu_k^{\text{real}} \in (\mu_j^{\text{comp}} - 0.5, \mu_j^{\text{comp}} + 0.5)\}$ and denoted it as z_j^{zi}, i.e., the proportion of false zeros to be introduced into taxon j's abundances in $\mathbf{Y}^{\text{comp}}$;
3. We randomly drew false zero indicators $I_{ij} \sim \text{Bernoulli}(z_j^{\text{zi}})$ independently for sample $i = 1, \dots, n$;

4. We set $Y_{ij}^{\text{zi}} = \max\left(Y_{ij}^{\text{comp}} \cdot I_{ij},\, \log_{10}(1.01)\right)$.

The reason why we set the minimum value of $\mathbf{Y}^{\text{zi}}$ to $\log_{10}(1.01)$ is that mbImpute step 1 sets the minimum log-transformed abundance to $\log_{10}(1.01)$ to facilitate the fitting of the Gamma-normal mixture model.

After applying mbImpute to $\mathbf{Y}^{\text{zi}}$ (mbImpute also used $\mathbf{X}$), we obtained $\mathbf{Y}^{\text{imp}}$ and evaluated the imputation performance by calculating the mean squared error (MSE) between $\mathbf{Y}^{\text{imp}}$ and $\mathbf{Y}^{\text{comp}}$:

$$\text{MSE}^{\text{mbImpute}} = \frac{1}{nm}\sum_{i=1}^{n}\sum_{j=1}^{m}(Y_{ij}^{\text{comp}} - Y_{ij}^{\text{imp}})^2$$

and the MSE between the zero-inflated matrix $\mathbf{Y}^{\text{zi}}$ and $\mathbf{Y}^{\text{comp}}$:

$$\text{MSE}^{\text{no imputation}} = \frac{1}{nm}\sum_{i=1}^{n}\sum_{j=1}^{m}(Y_{ij}^{\text{comp}} - Y_{ij}^{\text{zi}})^2 .$$

The results are summarized in Supplementary Fig. S3.

Simulation 4 for DA analysis on 16S rRNA data

We used the `R` package `sparseDOSSA` by Ren *et al.* (2016) to simulate the 16S rRNA sequencing data with known DA taxa. Specifically, we used the following command to obtain simulated data:

```
metadata <- matrix(rbinom(n = 100, size = 1, prob = 0.5), nrow = 1, ncol = 100)
simulated_data <- sparseDOSSA(number_features = 150, number_samples = 100,
percent_spiked = 0.3, UserMetadata = metadata)
```

In order to evaluate the performance of four DA methods (the Wilcoxon rank-sum test, ANCOM, metagenomeSeq, and DESeq2-phyloseq) before and after mbImpute is applied, we use three evaluation metrics: precision, recall, and F1 score. The results are summarized in Fig. 2e in the main text.

Simulation 5 for robustness to sequencing depth and outlier samples

To evaluate the robustness of mbImpute to sequencing depth and the existence of outlier samples, we performed simulation based on the 16S rRNA sequencing data of 54 healthy human stool samples in the R package `HMP16SData` (version 1.6.0). Below we describe the data generation process step by step.

Complete data generation

1. We randomly sampled 300 taxa in the 16S rRNA sequencing data that each has no more than 70% of zeros across samples, obtaining a 54×300 OTU count matrix.
2. We followed the data pre-processing steps of mbImpute (see Methods in the main text) to convert the OTU count matrix to a log-transformed normalized abundance matrix, denoted by $\mathbf{Y} = (Y_{ij}) \in \mathbb{R}^{54\times 300}$. We also collected the sample covariate matrix $\mathbf{X} \in \mathbb{R}^{54\times 2}$ and the phylogenetic distance matrix $\mathbf{D} \in \mathbb{Z}_{\geq 0}^{300\times 300}$.
3. We create a complete abundance matrix denoted by $\mathbf{Y}^{\text{comp}}$ with the same dimensions as $\mathbf{Y}$. Specifically, for each taxon j, we identified a set Ω_j of samples in which taxon j's original counts are not 0, and we denote the rest of the samples as Ω_j^c. From the j-th column of $\mathbf{Y}$, we copied the abundances in samples in Ω_j to the corresponding entries in the j-th column of $\mathbf{Y}^{\text{comp}}$, and we calculated the mean $\hat{\mu}_j$ and the standard deviation $\hat{\sigma}_j$ of the abundances in the samples in Ω_j. Then we sampled abundances for the samples in Ω_j^c independently from $\mathcal{N}(\hat{\mu}_j, \hat{\sigma}_j)$ to fill in the corresponding entries in the j-th column $\mathbf{Y}^{\text{comp}}$.
4. We transform $\mathbf{Y}^{\text{comp}}$ to a complete count matrix $\mathbf{M}^{\text{comp}}$ by setting the (i, j)-th entry as $M_{ij}^{\text{comp}} = \lfloor (10^{Y_{ij}^{\text{comp}}} - 1.01) \rfloor$.

Complete data adjusted by sequencing depth

1. Samples (rows) in $\mathbf{M}^{\text{comp}}$ have total counts around $2{,}000$. We varied the sequencing depth as $s = 1{,}000$, $2{,}000$, $5{,}000$, or $10{,}000$.

2. For each sequencing depth, we sampled a 300-dimensional count vector for sample i, $i = 1, \ldots, 54$, from a multinomial distribution, whose total is the sequencing depth and whose probability vector is $M_{i\cdot}^{\text{comp}} / (\sum_{j=1}^{300} M_{ij}^{\text{comp}})$, where $M_{i\cdot}^{\text{comp}}$ denotes the i-th row of $\mathbf{M}^{\text{comp}}$. We stacked the sampled vectors by row into a count matrix $\mathbf{M}_s^{\text{comp}}$, whose log-transformed abundance matrix is denoted by $\mathbf{Y}_s^{\text{comp}}$.

3. We transform $\mathbf{Y}_s^{\text{comp}}$ to a complete count matrix $\mathbf{M}_s^{\text{comp}}$ by setting the (i, j)-th entry as $(M_s)_{ij}^{\text{comp}} = \lfloor (10^{(Y_s)_{ij}^{\text{comp}}} - 1.01) \rfloor$.

Zero-inflated data generation

We introduced zero inflation to $\mathbf{Y}_s^{\text{comp}}$, following the same non-parametric procedure as in simulation 2. We denote the resulting zero-inflated matrix by $\mathbf{Y}_s^{\text{zi}}$ and the corresponding count matrix by $\mathbf{M}_s^{\text{zi}}$, $s = 1{,}000$, $2{,}000$, $5{,}000$, and $10{,}000$.

Zero-inflated data with outlier samples

1. Given $s = 2{,}000$, we use $\mathbf{Y}_s^{\text{zi}}$ to define the lowly abundant taxa as those that have mean abundances below the median and at least 10 non-zero abundances.

2. To generate one outlier sample, for the 62 lowly abundant taxa based on our definition, we set their abundances in the outlier sample to be 62 values randomly sampled from the top 100 maximum abundances (for each taxon, we calculated the maximum abundance). For the other taxa, we set their abundances in the outlier sample to zero.

3. To generate the second outlier sample, we repeated step 2.

4. We denote the resulting abundance matrices and count matrices with one or two outlier samples by $\mathbf{Y}^{\text{zi}}_{\text{1o}}$ and $\mathbf{M}^{\text{zi}}_{\text{1o}}$ or $\mathbf{Y}^{\text{zi}}_{\text{2o}}$ and $\mathbf{M}^{\text{zi}}_{\text{2o}}$, respectively.

Evaluation criteria for imputation accuracy

After applying mbImpute to the zero-inflated abundance matrices, each denoted by $\mathbf{Y}^{\text{zi}}$, we obtained the corresponding imputed abundances matrices, each denoted by $\mathbf{Y}^{\text{imp}}$, and we evaluated the imputation performance by calculating the mean squared error (MSE) between $\mathbf{Y}^{\text{imp}}$ and the corresponding complete abundance matrix $\mathbf{Y}^{\text{comp}}$:

$$\text{MSE}^{\text{mbImpute}} = \frac{1}{nm}\sum_{i=1}^{n}\sum_{j=1}^{m}(Y_{ij}^{\text{comp}} - Y_{ij}^{\text{imp}})^2$$

and the MSE between the zero-inflated matrix $\mathbf{Y}^{\text{zi}}$ and $\mathbf{Y}^{\text{comp}}$:

$$\text{MSE}^{\text{no imputation}} = \frac{1}{nm}\sum_{i=1}^{n}\sum_{j=1}^{m}(Y_{ij}^{\text{comp}} - Y_{ij}^{\text{zi}})^2 \,.$$

Note that the MSE is only calculated for non-outlier samples when outlier samples are introduced. The results are summarized in Supplementary Fig. S5.

Statistical definitions of DA taxa

Here we list three possible statistical definitions of DA taxa. For a given taxon j, we denote M_j^1 and M_j^2 as its (random) counts in two samples from subject groups 1 and 2. The first and most straightforward definition of DA is whether the null hypothesis

$$H_0 : \mathbb{E}[M_j^1] = \mathbb{E}[M_j^2]$$

is rejected. The Wilcoxon rank-sum test is based on this definition. However, this definition has an obvious drawback: it ignores the existence of excess false zeros, i.e., many observed zero values of M_j^1 and M_j^2 are not reliable. This drawback motivates the second definition, which introduces latent variables Z_j^1 and Z_j^2 indicating whether taxon j is detected in the two samples. The second definition relies on zero-inflated models: $M_j^r|(Z_j^r = 0) = 0$, $r = 1, 2$, and it defines taxon j as DA if

the null hypothesis

$$H_0 : \mathbb{E}[M_j^1|(Z_j^1 = 1)] = \mathbb{E}[M_j^2|(Z_j^2 = 1)]$$

is rejected. The metagenomSeq is based on this definition. A drawback of this definition is that many observations of M_j^1 and M_j^2 would not be used for testing this hypothesis, if their corresponding Z_j^1 and Z_j^2 are inferred as zeros, resulting in a power loss. To relieve this issue, imputation can be used to rescue the likely false zeros and infer their actual values, and mbImpute achieves this by borrowing information from similar samples, similar taxon, and sample covariates, leading to the third definition of DA taxa. Assuming that the imputation is successful, we denote M_j^{imp1} and M_j^{imp2} as taxon j's imputed counts, and Y_j^{imp1} and Y_j^{imp2} as the imputed abundances on the logarithmic scale, in the two samples. Then the third definition calls taxon j DA if the null hypothesis

$$H_0 : \mathbb{E}[M_j^{\text{imp1}}] = \mathbb{E}[M_j^{\text{imp2}}] \text{ or } H_0 : \mathbb{E}[Y_j^{\text{imp1}}] = \mathbb{E}[Y_j^{\text{imp2}}]$$

is rejected. This third definition is advantageous in that (1) compared with the first definition, it is less affected by the existence of false zeros, whose different proportions in the two subject groups may lead to false positive DA taxa (i.e., the taxa whose non-zero counts do not exhibit a clear difference between the two groups), and (2) compared with the second definition, it uses all the observations of taxon j for testing, leading to an increase in statistical power.

Fisher's exact test for detecting the enrichment of T2D- and CRC-related terms in DA taxa

We use functional terms in the GMrepo (Wu *et al.*, 2020) database to understand the DA taxa identified by DESeq2-phyloseq or mbImpute-empowered DESeq2-phyloseq. Among all functional terms, we identified two T2D-related terms:

- "The time period before the development of symptomatic diabetes. For example, certain risk factors can be observed in subjects who subsequently develop INSULIN RESISTANCE as in type 2 diabetes (DIABETES MELLITUS, TYPE 2)."
- "A cluster of symptoms that are risk factors for CARDIOVASCULAR DISEASES and TYPE 2 DIABETES MELLITUS. The major components of metabolic syndrome include ABDOMINAL OBESITY; atherogenic DYSLIPIDEMIA; HYPERTENSION; HYPERGLYCEMIA; INSULIN RESISTANCE; a proinflammatory state; and a prothrombotic (THROMBOSIS) state."

and one CRC-related term:

- "Tumors or cancer of the COLON or the RECTUM or both. Risk factors for colorectal cancer include chronic ULCERATIVE COLITIS; FAMILIAL POLYPOSIS COLI; exposure to ASBESTOS; and irradiation of the CERVIX UTERI."

Given a T2D- or CRC-related functional term, we performed the Fisher's exact test to check its enrichment in the DA taxa identified by DESeq2-phyloseq or mbImpute-empowered DESeq2-phyloseq from the corresponding T2D or CRC datasets. Specifically, we constructed a two-by-two contingency table (see below), whose rows indicate whether taxa are annotated by the functional term or not, and whose columns indicate whether taxa are identified as DA or not; each entry in the contingency table is the number of taxa satisfying the row and column conditions.

	identified as DA	not identified as DA
annotated by the term	a	b
not annotated by the term	c	d

Based on the above contingency table, the p-value of the Fisher's exact test is calculated as

$$p = \frac{\binom{a+b}{a}\binom{c+d}{c}}{\binom{a+b+c+d}{a+c}}.$$

A smaller p-value shows stronger evidence against the null hypothesis that there is no dependence between whether taxa are related to the term and whether taxa are identified as DA. In other words, a smaller p-value indicates a stronger enrichment of the term in the DA taxa.

To test the enrichment of each T2D-related term, we combined the DA taxa identified from the two T2D datasets by DESeq2-phyloseq or mbImpute-empowered DESeq2-phyloseq. To test the enrichment of each CRC-related term, we combined the DA taxa identified from the four CRC datasets by DESeq2-phyloseq or mbImpute-empowered DESeq2-phyloseq. The results are summarized in Table 1 in the main text.

Supplementary Table

	Wilcoxon	ANCOM	MetagenomeSeq	DESeq2-phyloseq	Omnibust test
Qin et al.	25	20	9	11	20
Karlsson et al.	5	6	0	1	4
Feng et al.	42	18	0	30	28
Yu et al.	19	22	4	54	23
Vogtmann et al.	0	0	0	53	2
Zeller et al.	25	34	10	36	20

Table S1: The number of DA taxa identified by each DA method in each dataset at the FDR threshold 5%. There are two T2D datasets (*Qin et al.* and *Karlsson et al.*) and four CRC datasets (*Feng et al.*, *Vogtmann et al.*, *Yu et al.*, and *Zeller et al.*)

Supplementary figures

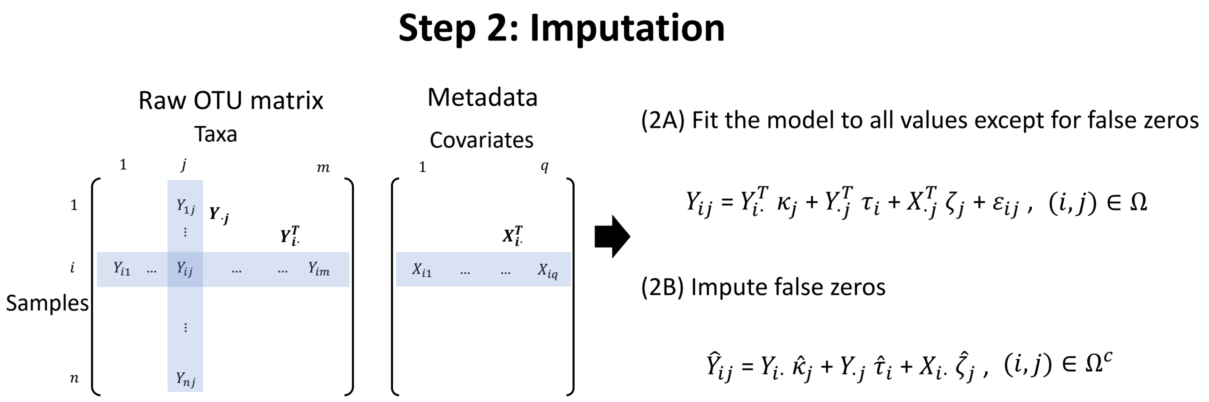

Figure S1: A diagram illustrating the step 2 of mbImpute. After step 1 that identifies likely false zeros, mbImpute borrows information across taxa, samples, and sample covariates to impute these likely false zeros. For details, see Methods in the main text.

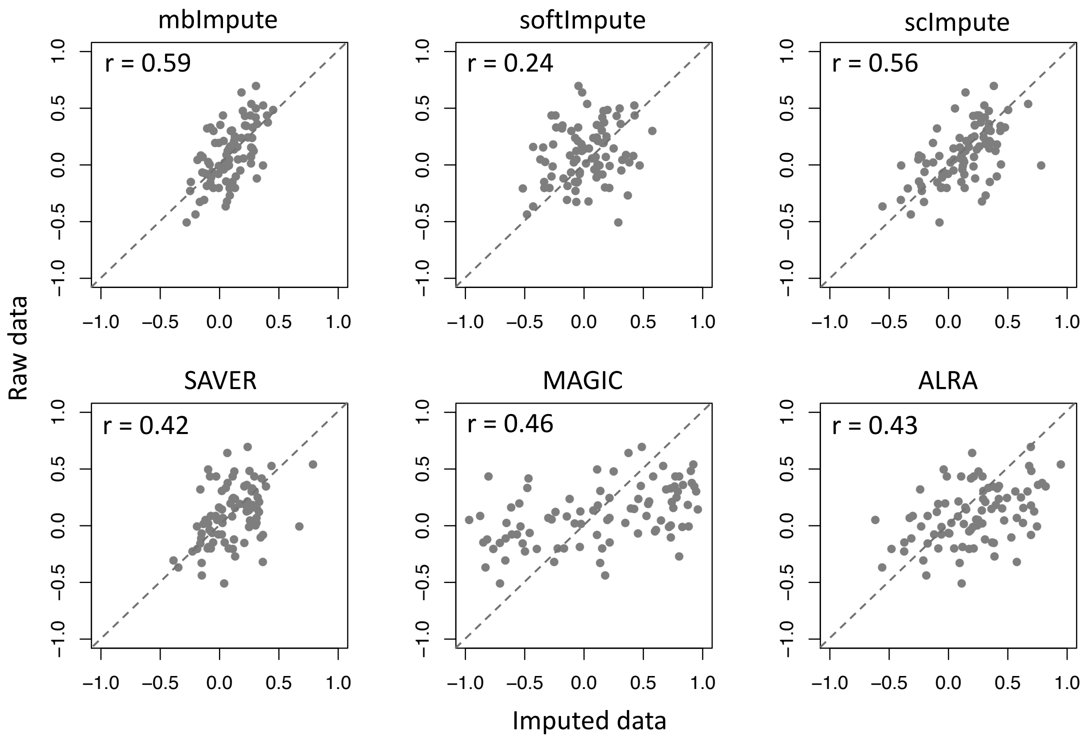

(a) CRC samples (Feng *et al.*, 2015)

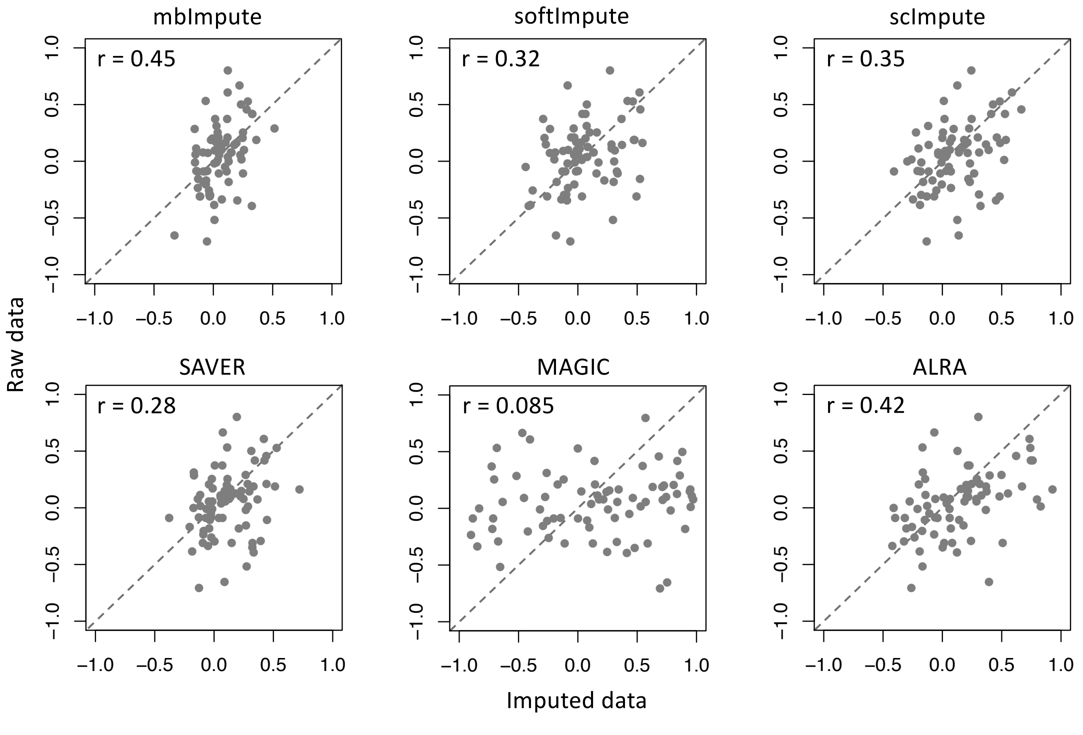

(b) Control samples (Feng *et al.*, 2015)

Figure S2: Correlations between phylogenetically closely related taxa in the raw data vs. those in the imputed data. For each pair of taxa connected by a path shorter than 4 branches in the phylogenetic tree, we computed their correlation in the raw data using their mutual non-zero abundances (vertical axis) and their correlation in the imputed data using all the abundances (horizontal axis). The Pearson correlation between these two sets of correlations is reported for each imputed dataset, showing that mbImpute achieves the highest correlation in both (a) the CRC samples and (b) the control samples.

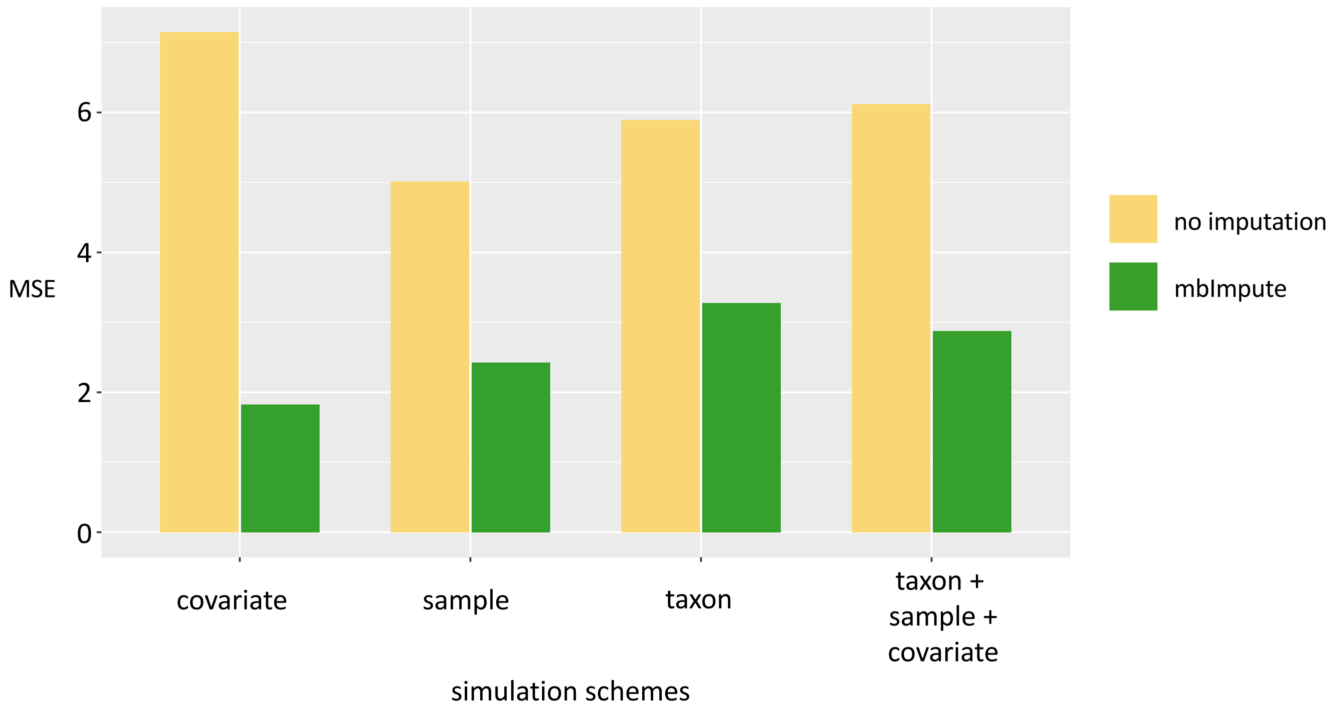

Figure S3: mbImpute reduces MSE under the four different simulation schemes in Simulation 3.

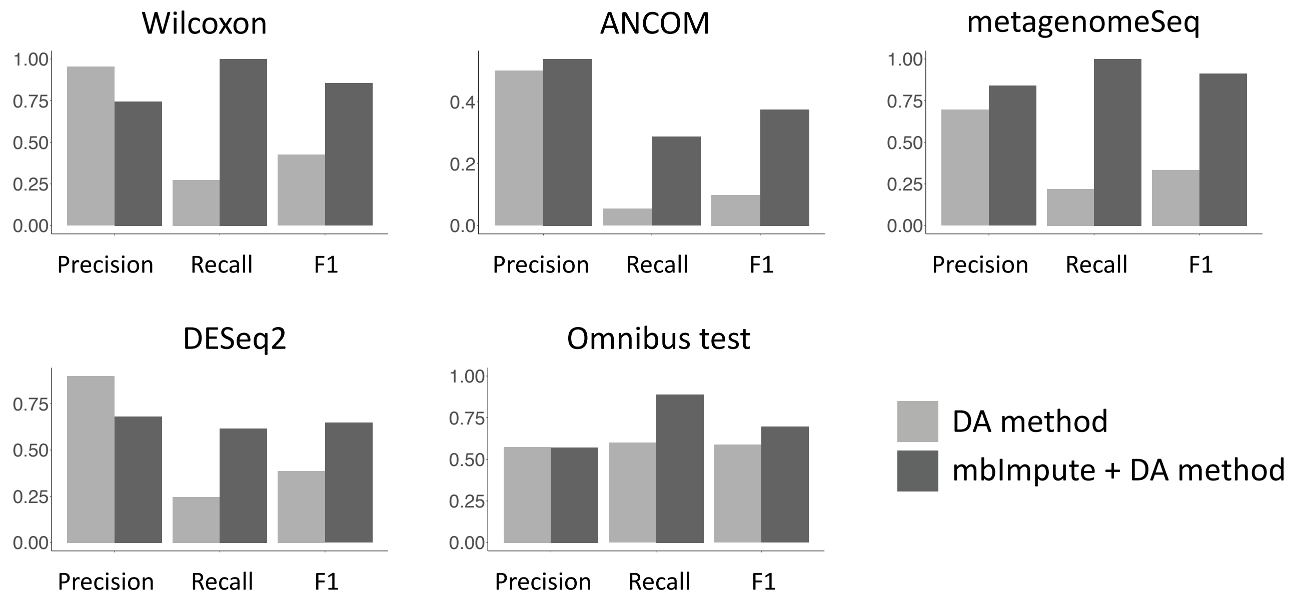

Figure S4: mbImpute enhances DA taxa identification in 16S simulation. Accuracy (Precision, recall, and F_1 scores) of four DA methods (Wilcoxon rank-sum test, ANCOM, metagenomeSeq, DESeq2-phyloseq, and Omnibus test) with an FDR threshold 0.1 on raw data (light color) and imputed data by mbImpute (dark color) in 16S data simulation.

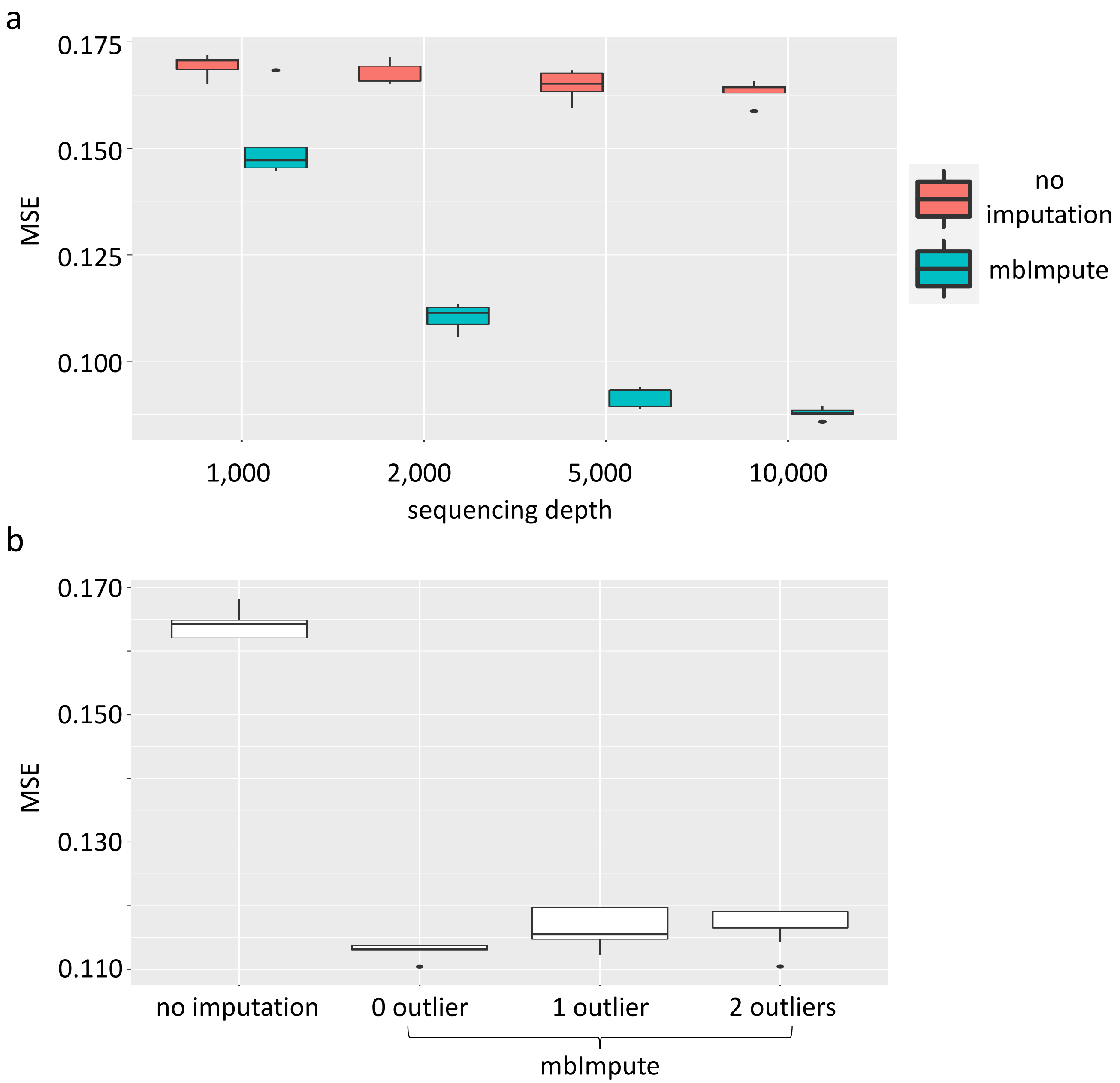

Figure S5: Robustness of mbImpute to sequencing depth and outlier samples. **(a)** Distributions of MSE before and after mbImpute is applied to simulated 16S rRNA sequencing data with four sequencing depths (number of reads per sample). **(b)** Distributions of MSE before and after mbImpute is applied to the data in (a) with the $2{,}000$-read per-sample sequencing depth and 0, 1 or 2 outlier samples added.

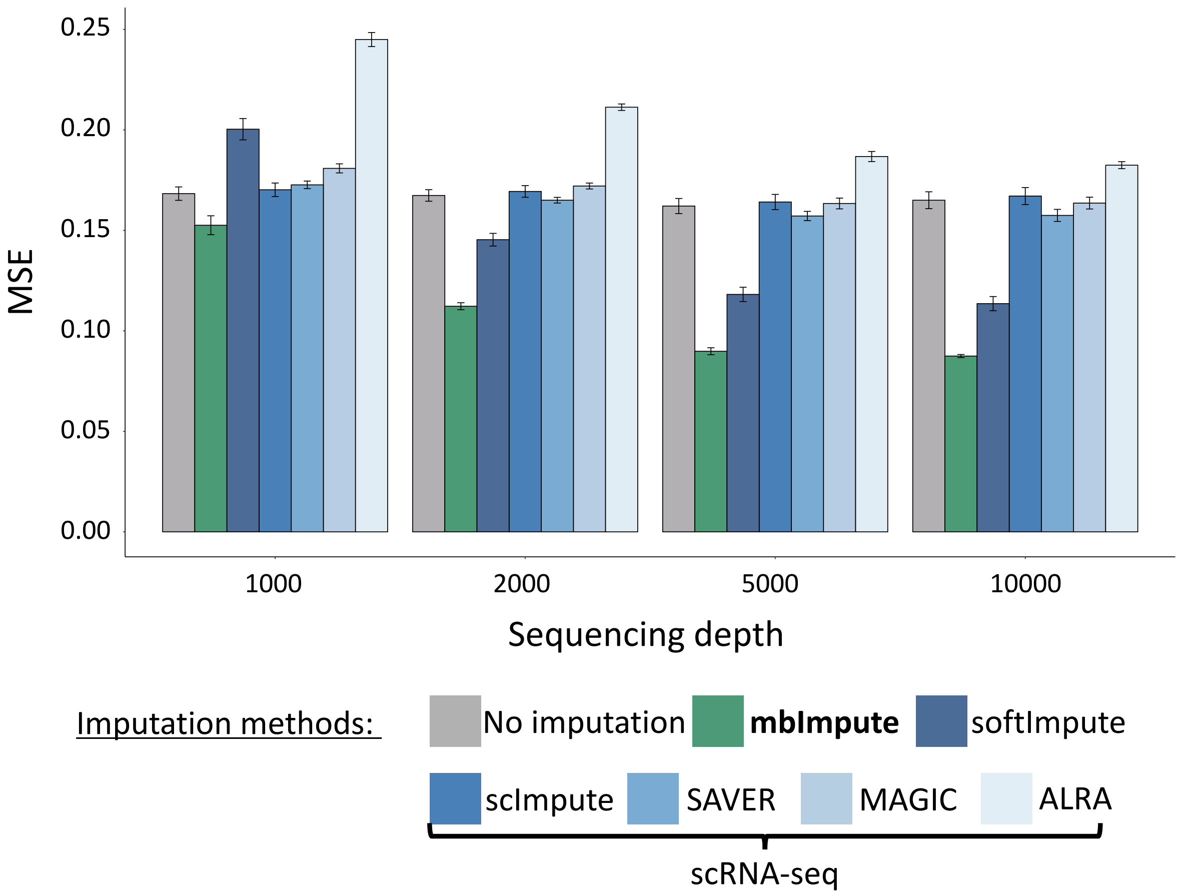

Figure S6: Effect of sequencing depth on different imputation methods. Mean squared error (MSE) between the complete data and the zero-inflated data ("No imputation," the baseline) or the imputed data by each imputation method (mbImpute, softImpute, scImpute, SAVER, MAGIC, and ALRA) under different simulated sequencing depth.

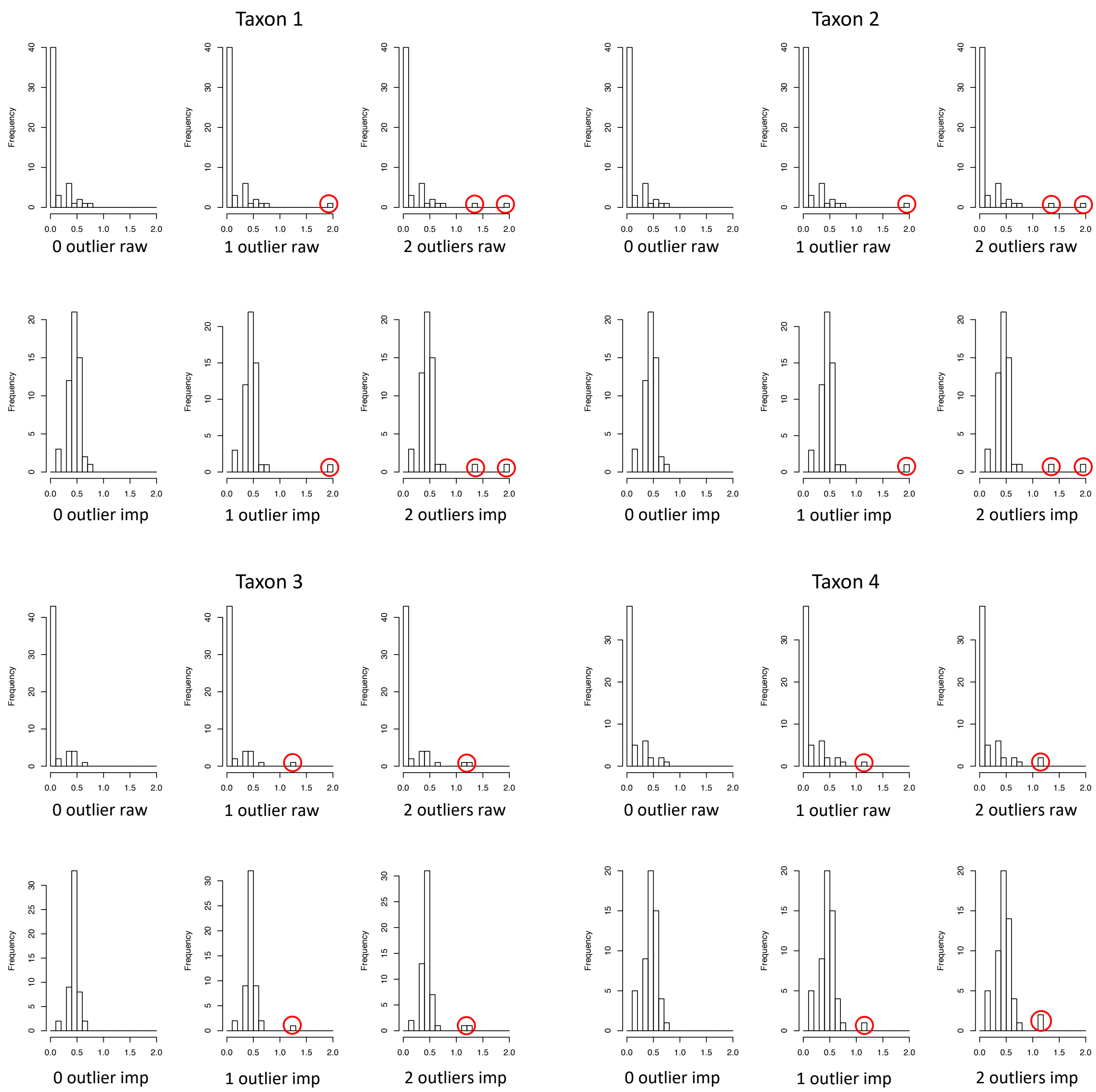

Figure S7: Abundance distributions of four taxa with one or two outlier samples before and after mbImpute is applied. For each taxon, the top row (”raw”) displays the abundance distributions with 0, 1, or 2 outlier samples before imputation, and the bottom row (”imp”) shows the distributions after mbImpute is applied. The abundance values in the outlier samples are marked by red circles.

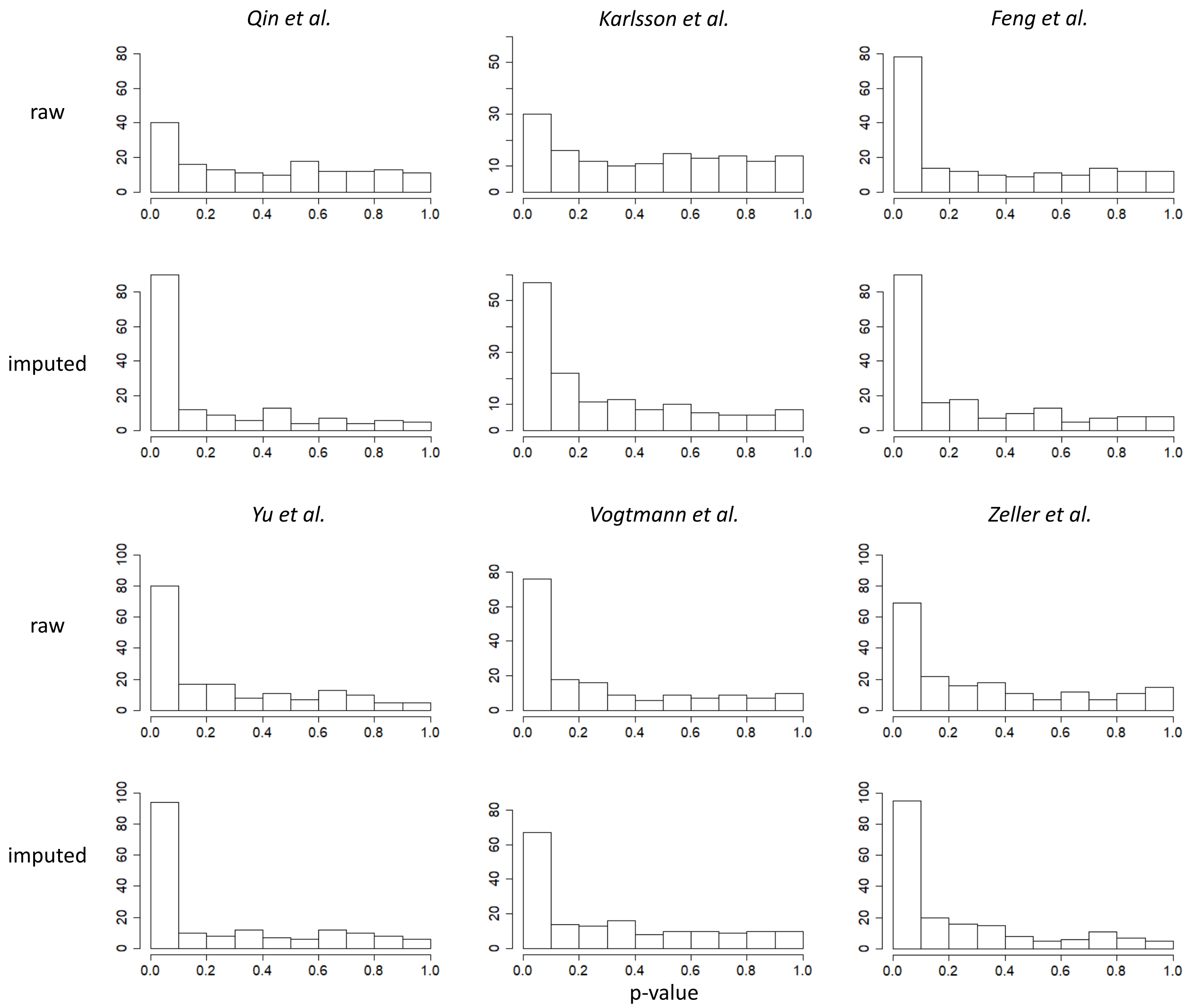

Figure S8: Distributions of DESeq2-phyloseq p-values before and after mbImpute is applied.

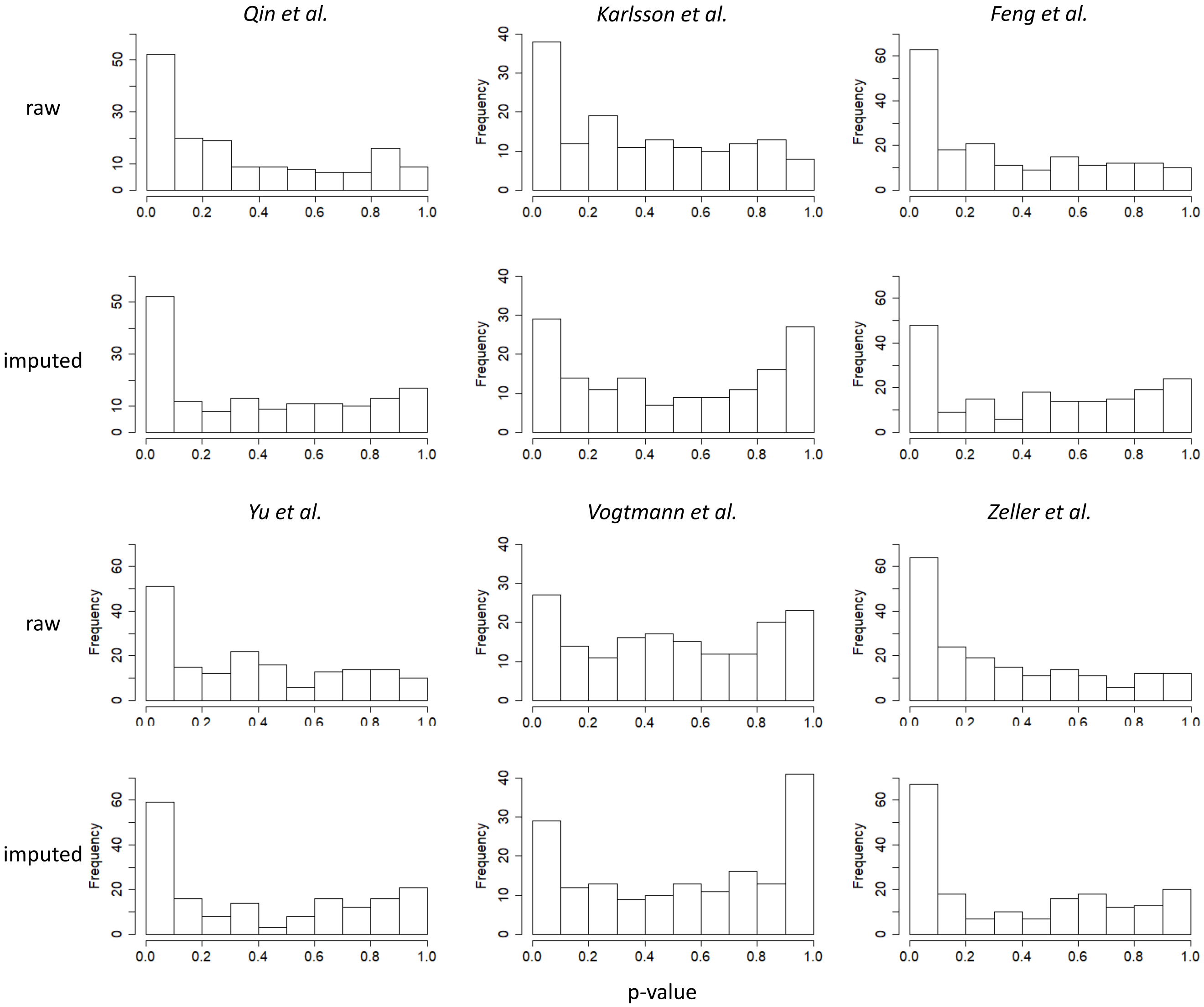

Figure S9: Distributions of Omnibus test p-values before and after mbImpute is applied.

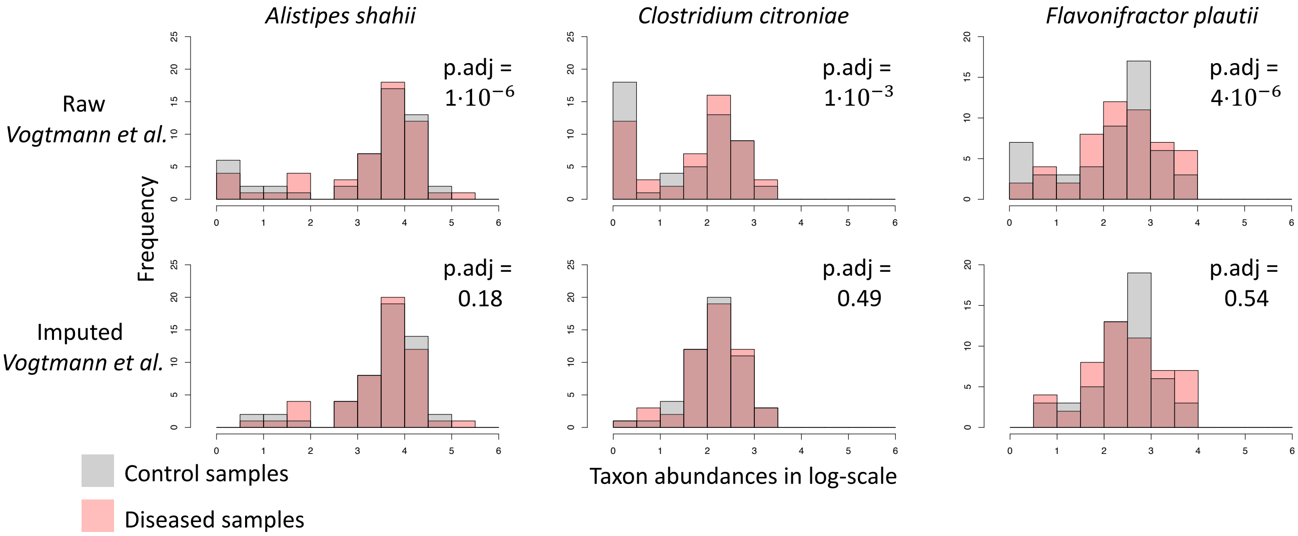

Figure S10: Abundance distributions of three taxa in control and CRC samples in *Vogtmann et al.* before and after mbImpute is applied. The three taxa, *Alistipes shahii*, *Clostridium citroniae*, and *Flavonifractor plautii*, are identified as DA by DESeq2-phyloseq before imputation but not as DA after imputation. Adjusted p-values are listed.

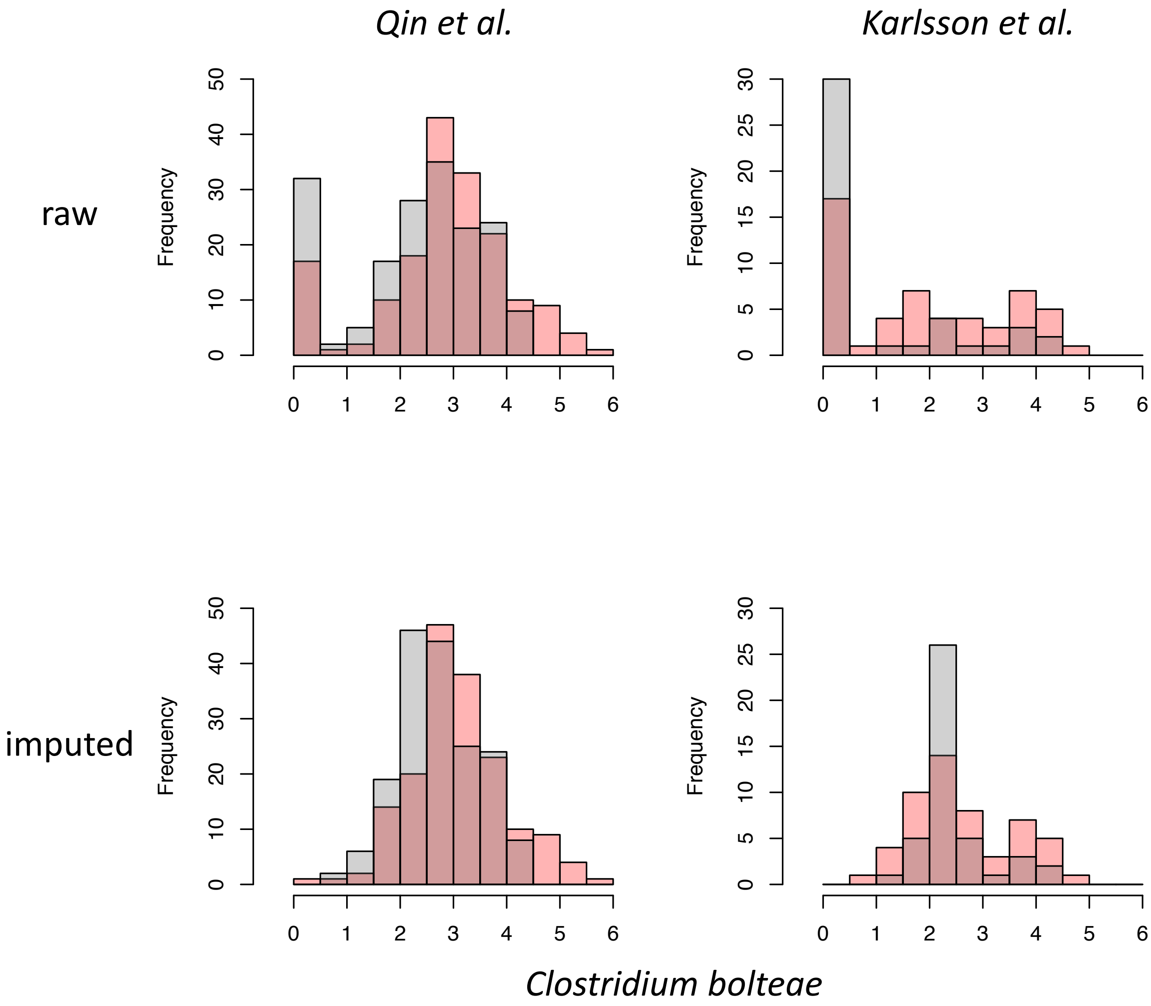

Figure S11: Distributions of *Clostridium bolteae* abundances before and after mbImpute is applied. Two T2D datasets, Qin *et al.* and Karlsson *et al.*, are included in this figure.

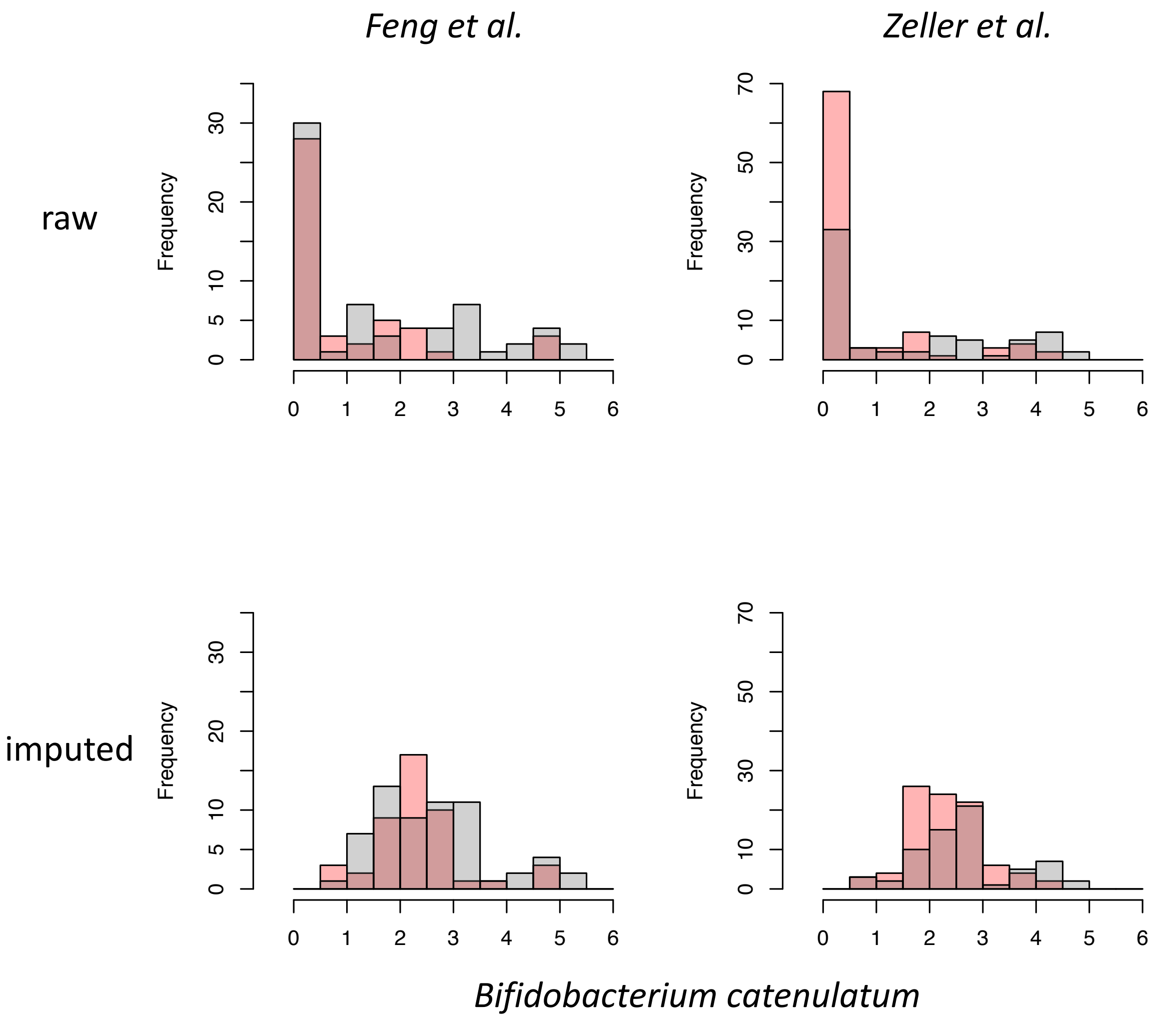

Figure S12: Distributions of *Bifidobacterium catenulatum* abundances before and after mbImpute is applied. Two CRC datasets, Feng *et al.* and Zeller *et al.*, are included in this figure.

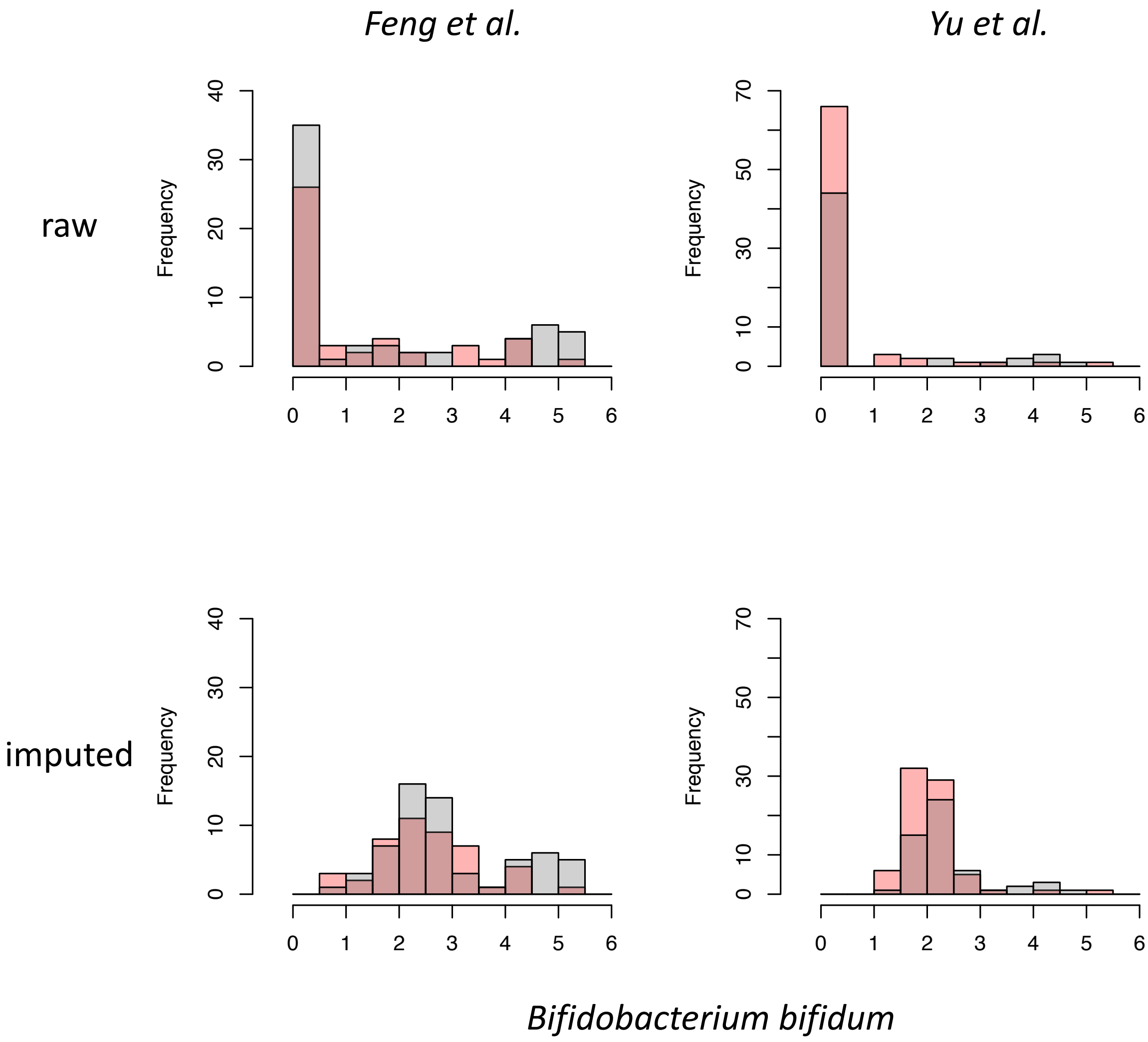

Figure S13: Distributions of *Bifidobacterium bifidum* abundances before and after mbImpute is applied. Two CRC datasets, Feng *et al.* and Yu *et al.*, are included in this figure.

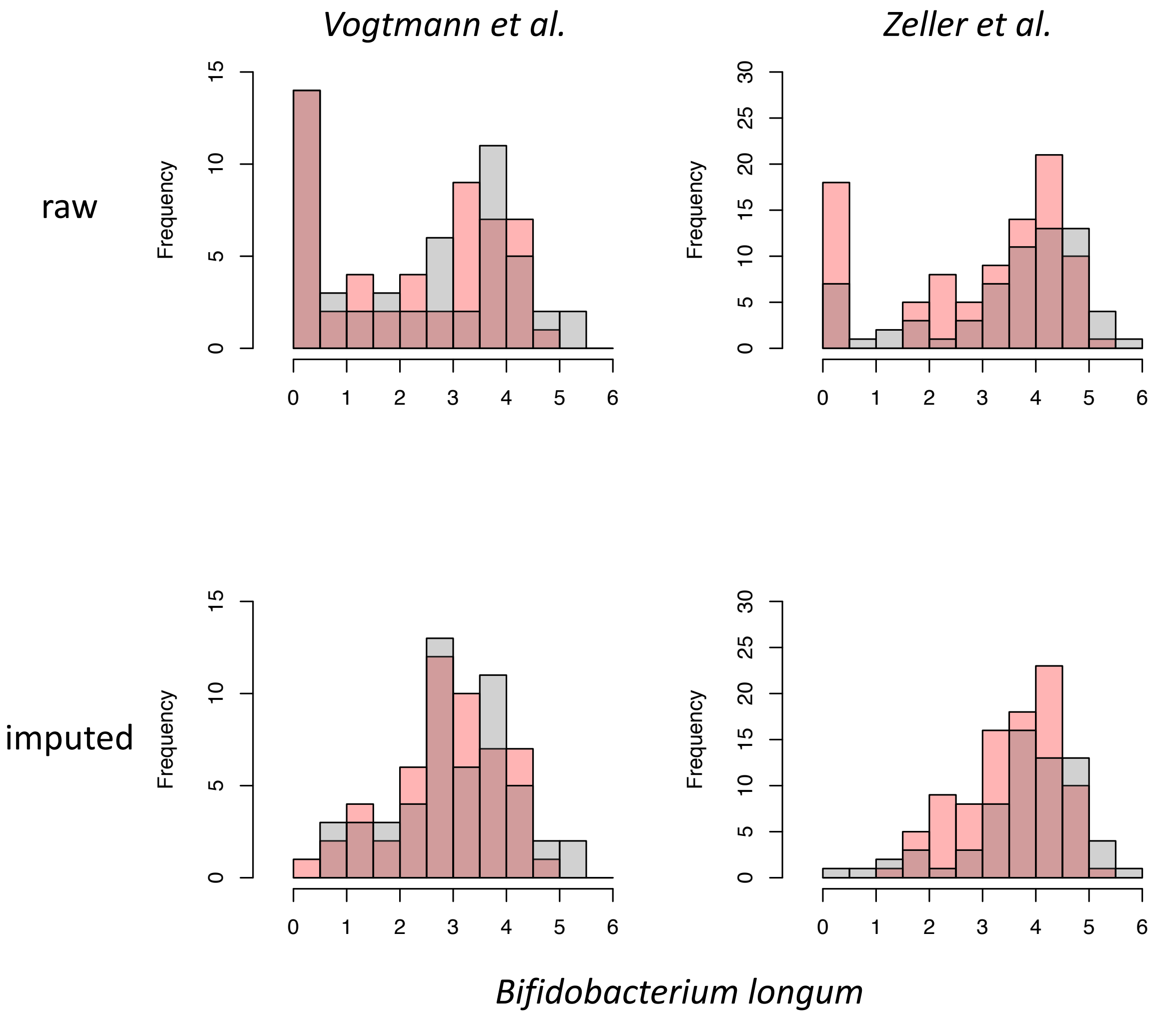

Figure S14: Distributions of *Bifidobacterium longum* abundances before and after mbImpute is applied. Two CRC datasets, Vogtmann *et al.* and Zeller *et al*., are included in this figure.

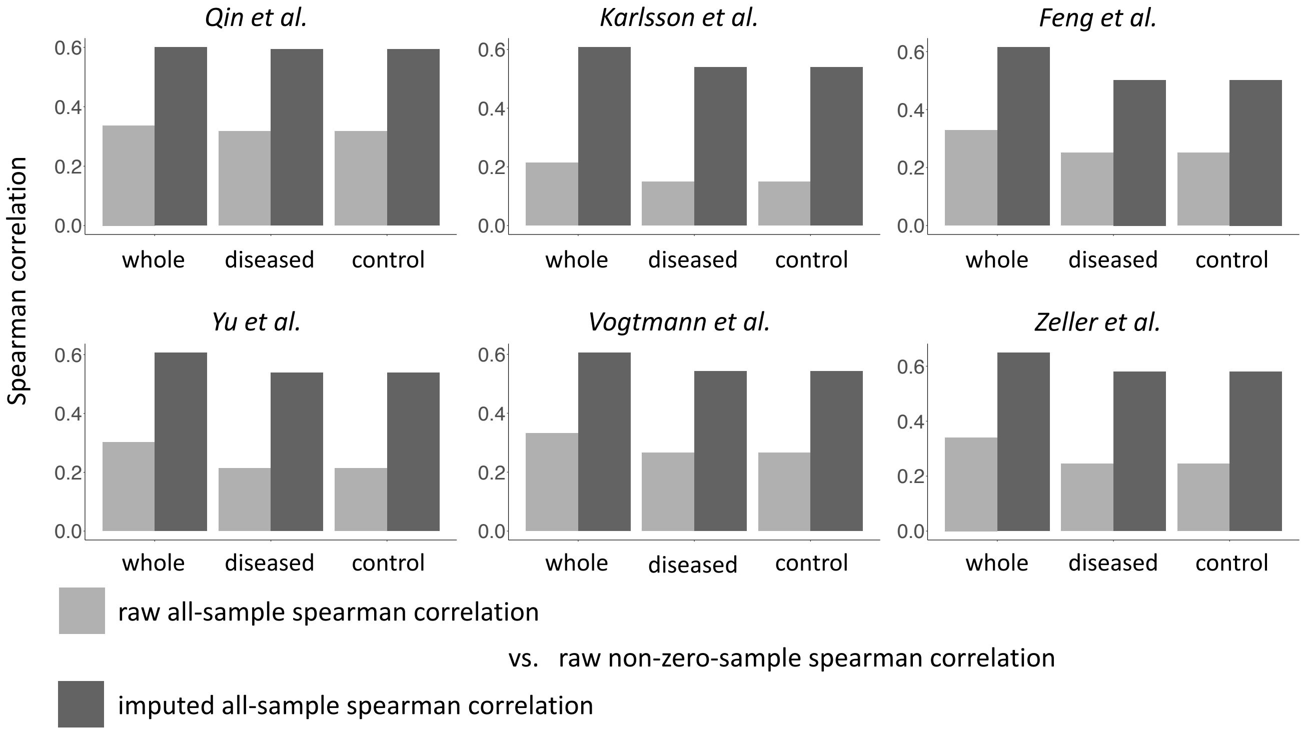

Figure S15: Each bar shows the Spearman correlation between taxon-taxon correlations in raw data (light gray) or imputed data (dark gray) using all samples and taxon-taxon correlations in raw data using non-zero samples only. The two correlations are calculated for two T2D datasets and four CRC datasets using diseased samples, control samples, and whole data.

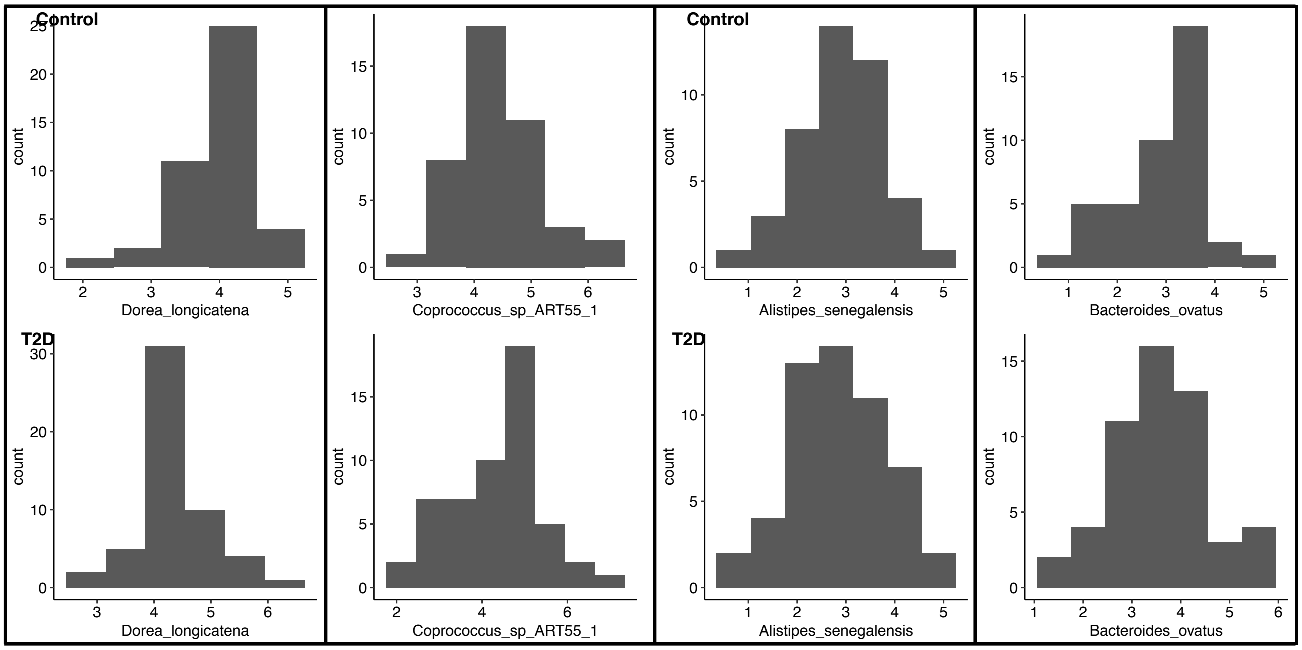

Figure S16: Distributions of four randomly chosen taxa from Karlsson *et al.* after mbImpute is applied. The imputed abundances are approximately normal.

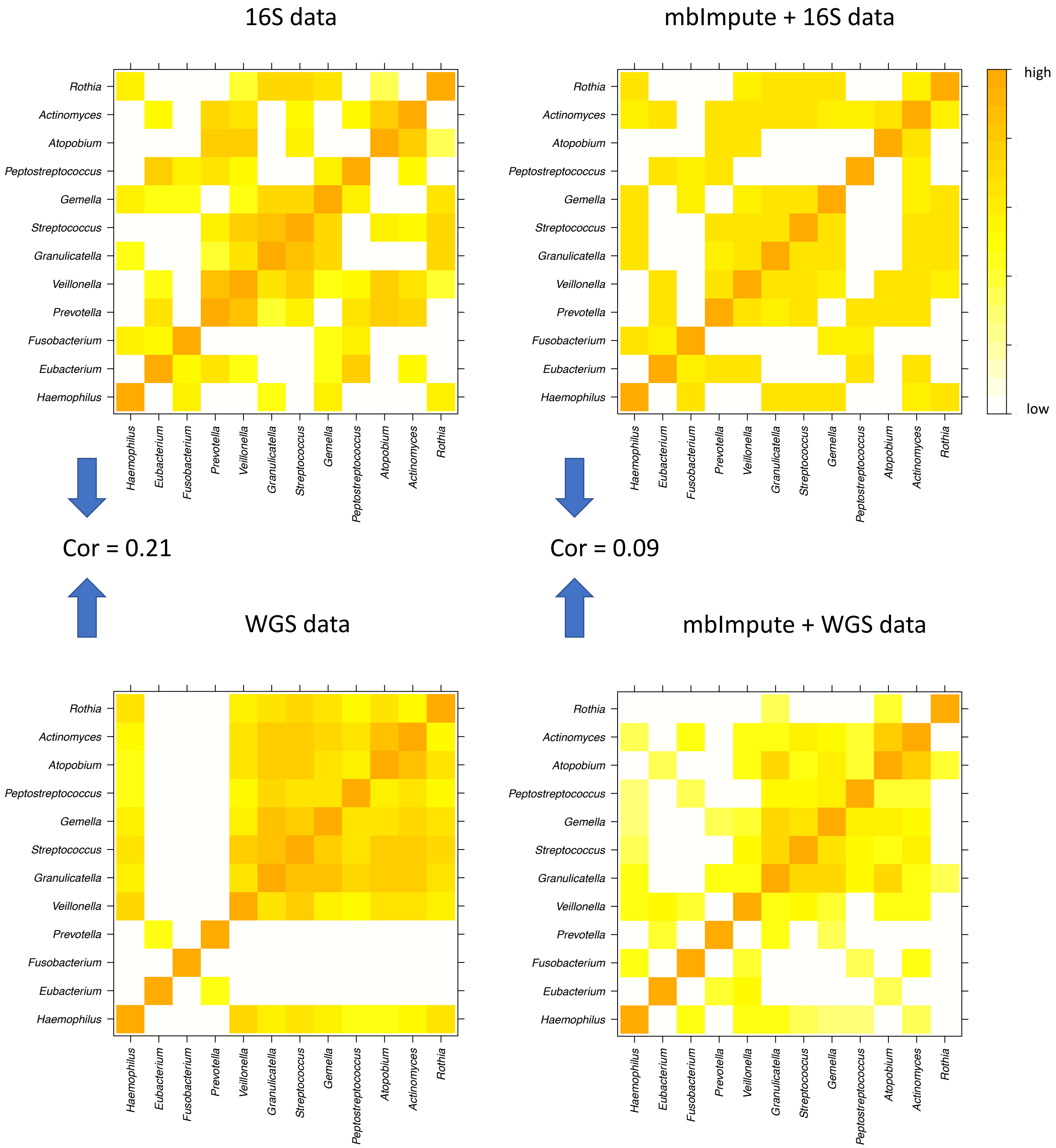

Figure S17: Four Pearson correlation matrices are calculated based on a common set of genus-level taxa's abundances in 16S healthy human oral samples and WGS healthy human stool samples, with or without using mbImpute as a preceding step. Before imputation, the Pearson correlation between the two correlation matrices is 0.21, and this correlation decreases to 0.09 after imputation. For illustration purposes, each heatmap shows square roots of Pearson correlations, with the bottom 40% of values truncated to 0.

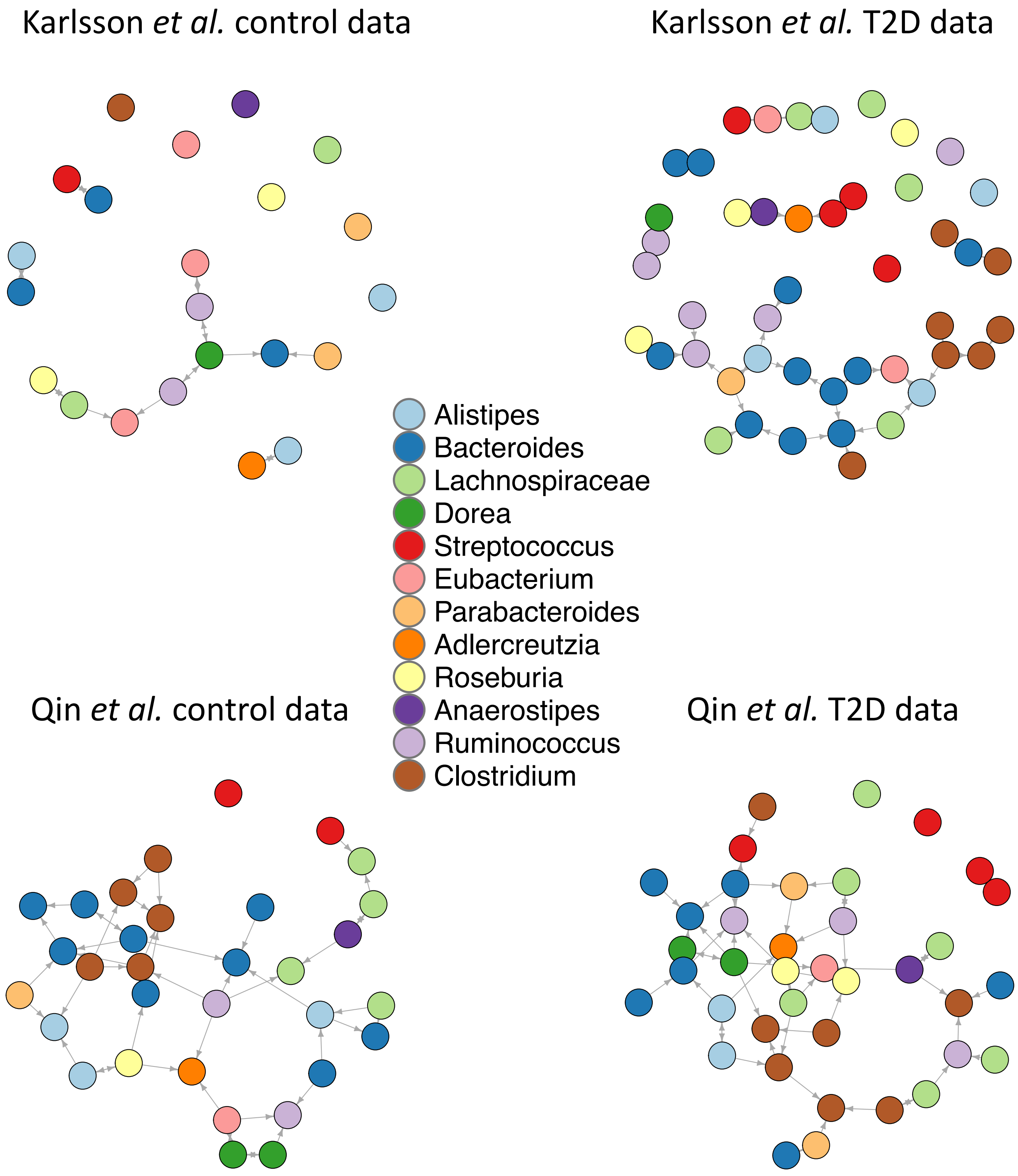

Figure S18: Visualization of genus-level taxon interaction networks constructed from two T2D datasets after mbImpute is applied. Four genus-level interaction networks constructed by the PC algorithm (Kalisch *et al.*, 2007) on the Karlsson *et al.* and Qin *et al.*'s control samples and T2D samples after mbImpute is applied.

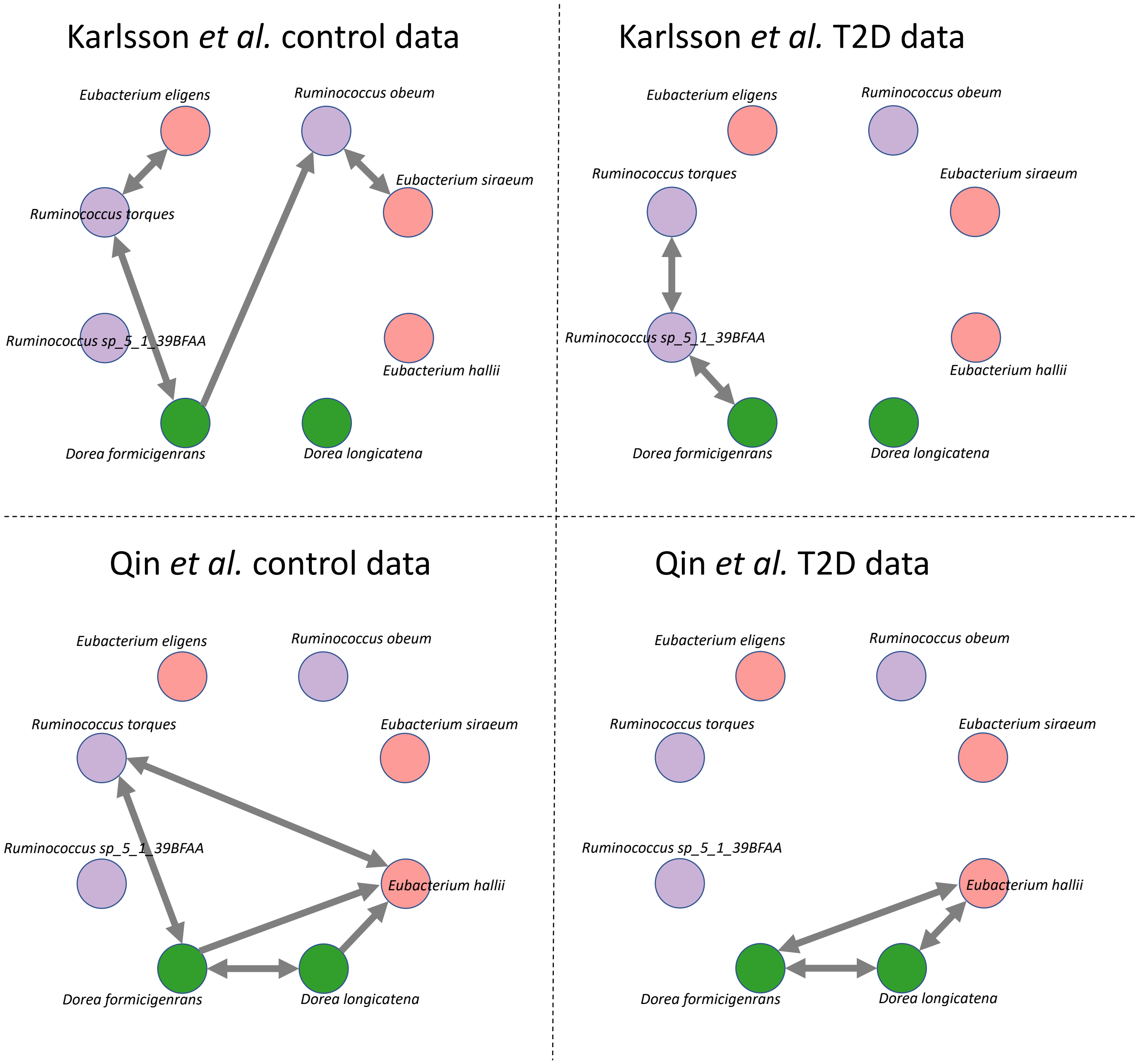

Figure S19: Visualization of species-level taxon interaction networks of three genera (*Eubacterium*, *Ruminococcus*, and *Dorea*) from two T2D datasets after mbImpute is applied. Four strain-level interaction networks constructed by the PC algorithm on the Karlsson *et al.* and Qin *et al.*'s control samples and T2D samples after mbImpute is applied.

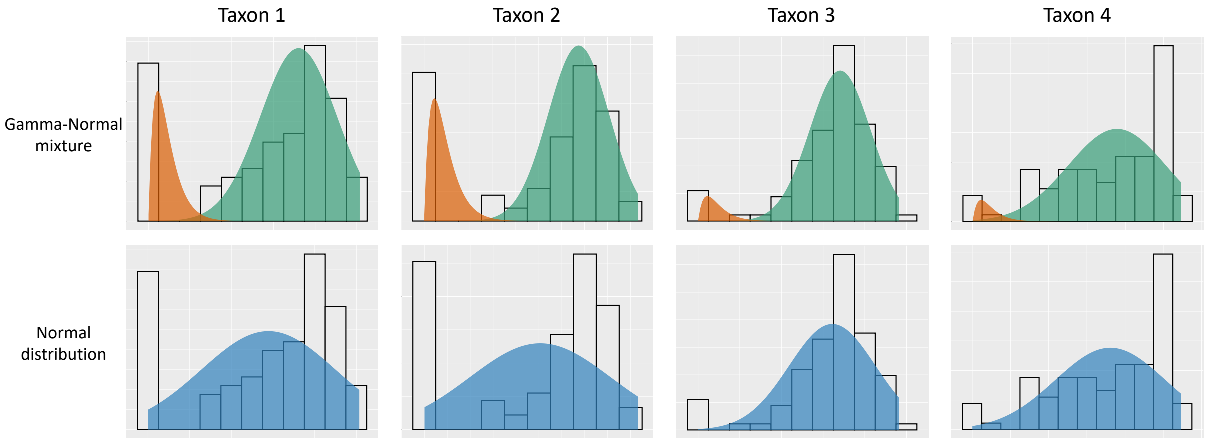

Figure S20: Abundance distributions of four taxa in the data from (Zeller *et al.*, 2014). The same histogram is displayed in each column to show each taxon's abundance distribution. Top panel: fitted Gamma-normal mixture model, where the red and green areas represent the Gamma and normal components, respectively. Bottom panel: fitted normal distribution represented by the blue area. For details, see Methods in the main text.

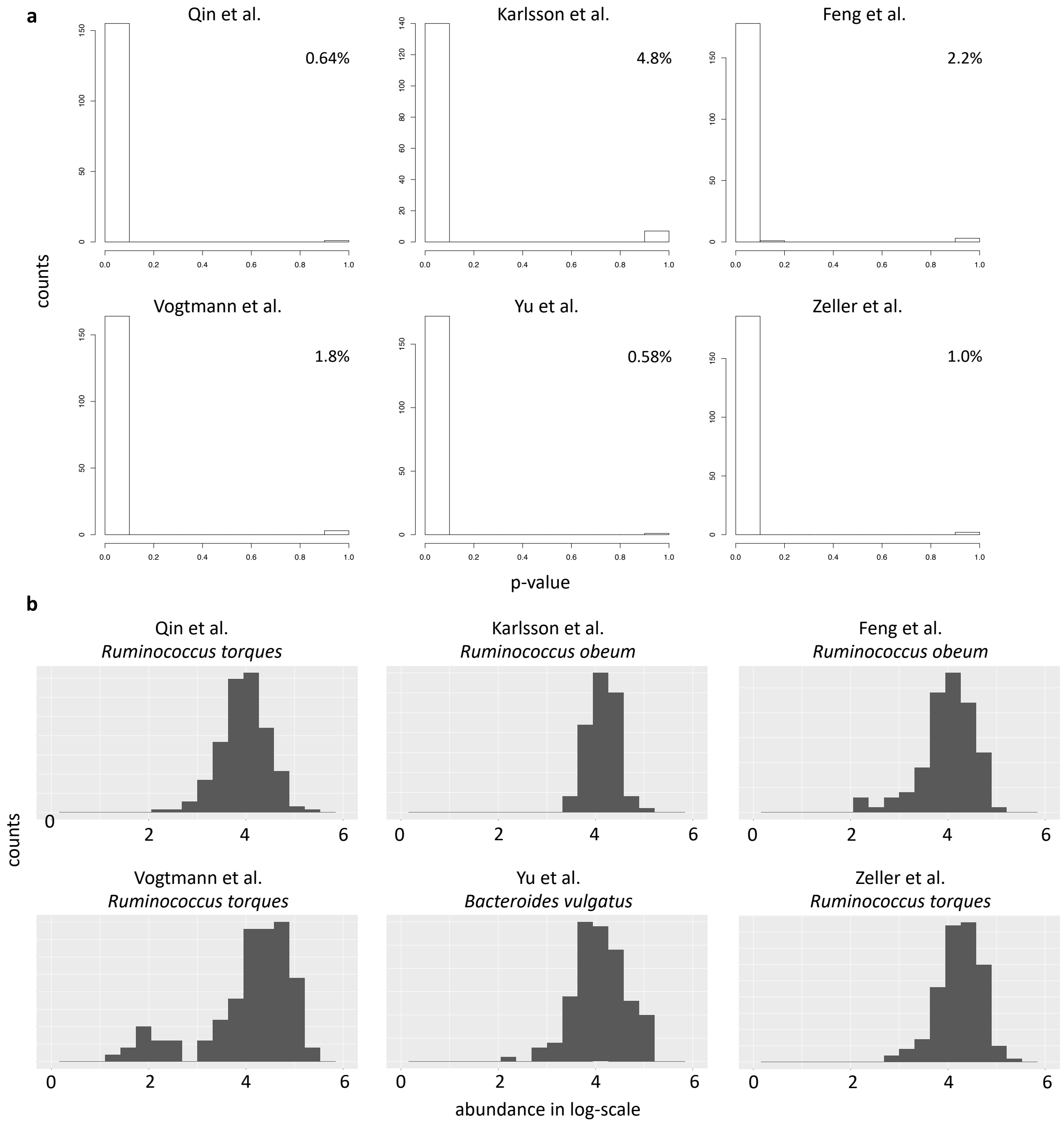

Figure S21: Outputs of step 1 of mbImpute on six real datasets. (a) Distributions of the LRT p-values in step 1 of mbImpute. Most p-values are close to 0. For each dataset, the percentage of taxa with > 0.05 p-values are shown at the top right. **(b)** Distributions of six example taxa, each of which has the LRT p-value > 0.05 in each dataset. For these taxa, mbImpute does not perform imputation.

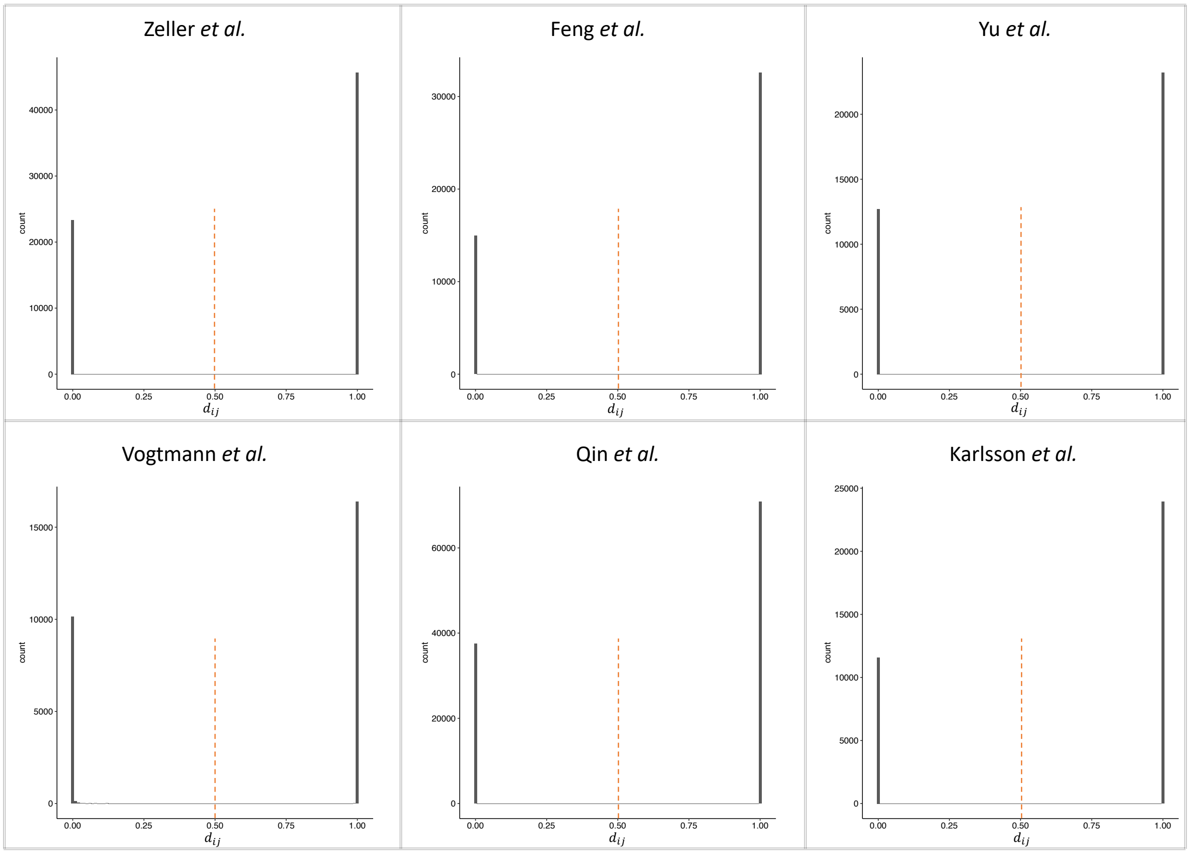

Figure S22: The distributions of d_{ij}'s calculated in step 1 of mbImpute applied to six real datasets. The 0.5 threshold is indicated by the red dotted line. For details, see Methods in the main text.

References

Allen-Vercoe, E., and Jobin, C. (2014). Fusobacterium and Enterobacteriaceae: important players for CRC?. *Immunology letters*, **162(2)**, 54-61.

Amato, K. R. (2017). An introduction to microbiome analysis for human biology applications. *American Journal of Human Biology*, **29(1)**.

Besser, J., Carleton, H. A., Gerner-Smidt, P., Lindsey, R. L., and Trees, E. (2018). Next-generation sequencing technologies and their application to the study and control of bacterial infections. *Clinical microbiology and infection*, **24(4)**, 335-341.

Casarin, R. C. V., Barbagallo, A., Meulman, T., Santos, V. R., Sallum, E. A., Nociti, F. H., ... and Gonçalves, R. B. (2013). Subgingival biodiversity in subjects with uncontrolled type-2 diabetes and chronic periodontitis. *Journal of periodontal research*, **48(1)**, 30-36.

Chen, J., King, E., Deek, R., Wei, Z., Yu, Y., Grill, D., and Ballman, K. (2017). An omnibus test for differential distribution analysis of microbiome sequencing data. *Bioinformatics*, **34(4)**, 643-651.

Dandona, P., Aljada, A., and Bandyopadhyay, A. (2004). Inflammation: the link between insulin resistance, *obesity and diabetes. Trends in immunology*, **25(1)**, 4-7.

Dulal, S., and Keku, T. O. (2014). Gut microbiome and colorectal Adenomas. *Cancer journal (Sudbury, Mass.)*, **20(3)**, 225.

Eraslan, G., Simon, L. M., Mircea, M., Mueller, N. S., and Theis, F. J. (2019). Single-cell RNA-seq denoising using a deep count autoencoder. *Nature communications*, **10(1)**, 390.

Feng, Q., *et al*. (2015). Gut microbiome development along the colorectal Adenoma-carcinoma sequence. *Nature communications*, **6**, 6528.

Friedman, J., Hastie, T., and Tibshirani, R. (2009). glmnet: Lasso and elastic-net regularized generalized linear models. *R package version*, **1(4)**.

Gong, W., Kwak, I. Y., Pota, P., Koyano-Nakagawa, N., & Garry, D. J. (2018). DrImpute: imputing dropout events in single cell RNA sequencing data. *BMC bioinformatics*, **19(1)**, 220.

Hastie, T., Mazumder, R., and Hastie, M. T. (2013). softImpute: Matrix Completion via Iterative Soft-Thresholded SVD. *R package version*, **1.4**

Holmes, E., Li, J. V., Athanasiou, T., Ashrafian, H., and Nicholson, J. K. (2011). Understanding the role of gut microbiome-host metabolic signal disruption in health and disease. *Trends in microbiology*, **19(7)**, 349-359.

Huang, M., Wang, J., Torre, E., Dueck, H., Shaffer, S., Bonasio, R., ... and Zhang, N. R. (2018). SAVER: gene expression recovery for single-cell RNA sequencing. *Nature methods*, **15(7)**, 539.

Jackman, S, (2017). pscl: Classes and Methods for R Developed in the Political Science Computational Laboratory. *R package version*, **1.5.2**

Jovel, J., *et al* (2016). Characterization of the gut microbiome using 16S or shotgun metagenomics. *Frontiers in microbiology*, **7**, 459.

Kalisch, M., and Bühlmann, P. (2007). Estimating high-dimensional directed acyclic graphs with the PC-algorithm. *Journal of Machine Learning Research*, **8(Mar)**, 613-636.

Karlsson, F. H., Tremaroli, V., Nookaew, I., Bergström, G., Behre, C. J., Fagerberg, B., ... and Bäckhed, F. (2013). Gut metagenome in European women with normal, impaired and diabetic glucose control. *Nature*, **498(7452)**, 99.

Kaul, A., Mandal, S., Davidov, O., and Peddada, S. D. (2017). Analysis of Microbiome Data in the Presence of Excess Zeros. *Frontiers in microbiology*, **8**, 2114. doi:10.3389/fmicb.2017.02114

Krijthe, J. H. (2015). Rtsne: T-distributed stochastic neighbor embedding using Barnes-Hut implementation. *R package version 0.13*, **URL** https://github. com/jkrijthe/Rtsne.

Larsen, N., Vogensen, F. K., Van Den Berg, F. W., Nielsen, D. S., Andreasen, A. S., Pedersen, B. K., ... and Jakobsen, M. (2010). Gut microbiota in human adults with type 2 diabetes differs from non-diabetic adults. *PloS one*, **5(2)**, e9085.

Ley, R. E., Bäckhed, F., Turnbaugh, P., Lozupone, C. A., Knight, R. D., and Gordon, J. I. (2005). Obesity alters gut microbial ecology. *Proceedings of the National Academy of Sciences*, **102(31)**, 11070-11075.

Li, H. (2015). Microbiome, metagenomics, and high-dimensional compositional data analysis. *Annual Review of Statistics and Its Application*, **2**, 73-94.

Li, W. V., and Li, J. J. (2018). An accurate and robust imputation method scImpute for single-cell RNA-seq data. *Nature communications*, **9(1)**, 997.

Li, W. V., and Li, J. J. (2019). A statistical simulator scDesign for rational scRNA-seq experimental design, *Bioinformatics*, **35(14)**, i41–i50,

Mandal, S., Van Treuren, W., White, R. A., Eggesbo, M., Knight, R., and Peddada, S. D. (2015). Analysis of composition of microbiomes: a novel method for studying microbial composition. *Microbial ecology in health and disease*, **26(1)**, 27663.

Martínez, I., Lattimer, J. M., Hubach, K. L., Case, J. A., Yang, J., Weber, C. G., ... and Haub, M. D. (2013). Gut microbiome composition is linked to whole grain-induced immunological improvements. The ISME journal, **7(2)**, 269.

Nakatsu, G., *et al* (2015). Gut mucosal microbiome across stages of colorectal carcinogenesis. *Nature communications*, **6**, 8727.

Nguyen, N. T., Nguyen, X. M. T., Lane, J., and Wang, P. (2011). Relationship between obesity and diabetes in a US adult population: findings from the National Health and Nutrition Examination Survey, 1999–2006. *Obesity surgery*, **21(3)**, 351-355.

van Nimwegen, K. J., van Soest, R. A., Veltman, J. A., Nelen, M. R., van der Wilt, G. J., Vissers, L. E., and Grutters, J. P. (2016). Is the $1000 genome as near as we think? A cost analysis of next-generation sequencing. *Clinical chemistry*, **clinchem-2016**.

Noble, D., Mathur, R., Dent, T., Meads, C., and Greenhalgh, T. (2011). Risk models and scores for type 2 diabetes: systematic review. *Bmj*, **343**, d7163.

Pasolli, E., *et al.*, (2017). Accessible, curated metagenomic data through ExperimentHub. *Nature methods*, **14(11)**, 1023

Qin, J., Li, Y., Cai, Z., Li, S., Zhu, J., Zhang, F., ... and Peng, Y. (2012). A metagenome-wide association study of gut microbiota in type 2 diabetes. *Nature*, **490(7418)**, 55.

Remely, M., Dworzak, S., Hippe, B., Zwielehner, J., Aumüller, E., Brath, H., and Haslberger, A. (2013). Abundance and diversity of microbiota in type 2 diabetes and obesity. *J Diabetes Metab*, **4(253)**, 2.

Ren, B., Schwager, E., Tickle, T. L., and Huttenhower, C. SparseDOSSA: Sparse data observations for simulating synthetic abundance. 2016.

Samuel, B. S., and Gordon, J. I. (2006). A humanized gnotobiotic mouse model of host-archaeal-bacterial mutualism. *Proceedings of the National Academy of Sciences*, **103(26)**, 10011-10016.

Sanapareddy, N., *et al.*, (2012). Increased rectal microbial richness is associated with the presence of colorectal Adenomas in humans. *The ISME journal*, **6(10)**, 1858.

Savage, D. C. (1977). Microbial ecology of the gastrointestinal tract. *Annual Reviews in Microbiology*, **31(1)**, 107-133.

Semova, I., Carten, J. D., Stombaugh, J., Mackey, L. C., Knight, R., Farber, S. A., and Rawls, J. F. (2012). Microbiota regulate intestinal absorption and metabolism of fatty acids in the zebrafish. *Cell host & microbe*, **12(3)**, 277-288.

Shen, X. J., *et al.*, (2010). Molecular characterization of mucosal adherent bacteria and associations with colorectal Adenomas. *Gut microbes*, **1(3)**, 138-147.

Sobhani, I., *et al.*, (2011). Microbial dysbiosis in Colorectal Cancer (CRC) patients. *PloS one*, **6(1)**, e16393.

Turnbaugh, P. J., Ley, R. E., Mahowald, M. A., Magrini, V., Mardis, E. R., and Gordon, J. I. (2006). An obesity-associated gut microbiome with increased capacity for energy harvest. *nature*, **444(7122)**, 1027.

Vogtmann, E., Hua, X., Zeller, G., Sunagawa, S., Voigt, A. Y., Hercog, R., ... and Sinha, R. (2016). Colorectal cancer and the human gut microbiome: reproducibility with whole-genome shotgun sequencing. *PloS one*, **11(5)**, e0155362.

Waese, J., Provart, N. J., and Guttman, D. S. (2017). Topo-phylogeny: Visualizing evolutionary relationships on a topographic landscape. *PloS one*, **12(5)**, e0175895.

Wang, T., Cai, G., Qiu, Y., Fei, N., Zhang, M., Pang, X., ... and Zhao, L. (2012). Structural segregation of gut microbiota between colorectal cancer patients and healthy volunteers. *The ISME journal*, **6(2)**, 320.

Weir, T. L., Manter, D. K., Sheflin, A. M., Barnett, B. A., Heuberger, A. L., and Ryan, E. P. (2013). Stool microbiome and metabolome differences between Colorectal Cancer patients and healthy adults. *PloS one*, **8(8)**, e70803.

Wu, N., Yang, X., Zhang, R., Li, J., Xiao, X., Hu, Y., ... and Luan, C. (2013). Dysbiosis signature of fecal microbiota in colorectal cancer patients. *Microbial ecology*, **66(2)**, 462-470.

Wu, S., Sun, C., Li, Y., Wang, T., Jia, L., Lai, S., ... & Chen, W. H. (2020). GMrepo: a database of curated and consistently annotated human gut metagenomes. *Nucleic acids research*, **48(D1)**, D545-D553.

Xia, F., Chen, J., Fung, W. K., and Li, H. (2013). A logistic normal multinomial regression model for microbiome compositional data analysis. *Biometrics*, **69(4)**, 1053-1063.

Xiao, J., Chen, L., Johnson, S., Zhang, X., and Chen, J. C. (2018). Predictive modeling of microbiome data using a phylogeny-regularized generalized linear mixed model. *Frontiers in microbiology*, **9**, 1391.

Xu, L., Paterson, A. D., Turpin, W., and Xu, W. (2015). Assessment and selection of competing models for zero-inflated microbiome data. *PloS one*, **10(7)**, e0129606.

Yang, Y., Cai, Q., Zheng, W., Steinwandel, M., Blot, W. J., Shu, X. O., and Long, J. (2019). Oral microbiome and obesity in a large study of low-income and African-American populations. *Journal of oral microbiology*, **11(1)**, 1650597.

Yu, J., Feng, Q., Wong, S. H., Zhang, D., yi Liang, Q., Qin, Y., ... and Wang, X. (2017). Metagenomic analysis of faecal microbiome as a tool towards targeted non-invasive biomarkers for colorectal cancer. *Gut*, **66(1)**, 70-78.

Zeller, G., *et al.*, (2014). Potential of fecal microbiota for early-stage detection of Colorectal Cancer. *Molecular systems biology*, **10(11)**, 766.

Zhang, X., Mallick, H., and Yi, N. (2016). Zero-inflated negative binomial regression for differential abundance testing in microbiome studies. *Journal of Bioinformatics and Genomics*, **(2 (2))**.
