# Supplementary material for: mbImpute: an accurate and robust imputation method for microbiome data
Source: Genome Biol. 2021 Jun 28;22:192. doi: 10.1186/s13059-021-02400-4 (PMC8240317; doi:10.1186/s13059-021-02400-4)
Supplement: Supplementary file 8 — Additional file 8 Review history. [file 13059_2021_2400_MOESM8_ESM.docx]

**1^st^ round**

**Reviewer 1**

This manuscript proposed mbImpute, a novel imputation method to selectively recover observed zero counts in 16S and metagenomic sequence data. It also promotes the DESeq2-phyloseq as the subsequent differential abundance analysis method. This work is important and timely. It can be applied by a wide range of microbiome projects. I can follow the manuscript well. My comments and questions are as follows.

1. Are the imputation results impacted by the sequence depth? The manuscript has not discussed this. Based on the default normalization, the depth information is lost, which means the imputed abundances are not affected by the sequence depths. Is this behavior expected? Please comment.

2. Can you give the definition of Wasserstein distance used in the main texts? I am confused about the main texts "… between the two-dimensional distribution" but the supplementary material mentioned a one-dimensional distribution "\bar{Y_{.1}}^{imp} / sd_{.1}^{imp}.

3. "… only DESeq2-phyloseq identifies DA taxa in all datasets." I observed that DESeq2-phyloseq detected >50 DA taxa in Vogtmann CRC study, while other methods detect none. How many DA taxa can be detected by mbImpute-empowered DESeq2 method? To confirm that DESeq2-phyloseq is performing well, authors can include the top findings from DESeq2-phyloseq and mbImpute-empowered DESeq2-phyloseq.

4. Page 6, line 52, TEDDY is for Type-1 diabetes. The authors seem to treat it like T2D.

5. The biorxiv version of mbImpute used ZINB-GLM (or NB-GLM) to detect taxa. Can authors comment on why it is not benchmarked in this manuscript?

6. The authors used the number of branches connecting two taxa in the phylogenetic tree as a distance. Have the authors considered directly using distances based on the tree branch lengths?

**Reviewer 2**

The authors proposed an imputation method for microbiome data which have extensive zeros. They used a Gamma-normal mixture model to fit the data and then calculate the probability of missing values which they termed as non-biological zeros. Finally they used linear model to predict the zero values by borrowing information from similar samples, taxa, and covariates. Through comprehensive simulation studies and real data analyses, the new method, mbimpute, shows better performance than available imputation methods that were designated for other types of data, e.g., single-cell RNA-seq data. The proposed method would be useful in the relevant research area.

Major concerns:

1. Distinguishing non-biological zeros from biological zeros in metagenomics studies is challenging. If the authors can elaborate that the new method can handle this issue it would be great.

2. Several DA methods have been developed for zero-inflated microbiome data. Please include the Omnibus test (Chen et al. 2018) for comparison.

3. It seems that for some dataset fewer taxa are detected after applying mbimpute than without imputation, while for some cases more taxa are found for imputed data. Please explain.

4. In the section of "mbImpute empowers DESeq2-phyloseq in DA taxon analysis" they mentioned that there were only 17 taxa in overlap (page 7, line 7). Please display the two sets of taxa in a table, and examine whether there is some taxa (detected by DESeq2-phyloseq but missed by mbImpute-empowered way) that has biologically meaning with the CRC. In other word, due to the imputation some true signal might be missed/distorted.

5. In the section of "mbImpute preserves distributional characteristics of taxa's non-zero abundances and recovers downsampling zeros" they used Pearson correlation. A non-parametric correlation might be more appropriate when there are extreme values in microbial data, which is quite often.

6. In the section of "mbImpute increases the similarity of microbial community structure between 16S rRNA and WGS data" they used two stool samples, from these two types of technologies. What would happen for two quite different samples, e.g., one from natural environment using 16S rRNA and one from human gut based on WGS? Would the similarity between these two samples increased after imputation? If so, the increased similarity due to the imputation does not reflect the true relationship between the samples. They need to focus on the similarity between the samples (different or alike) under the same sequence technology and examine the impact of imputation.

7. They claimed that mbImpute is robust to the existence of outlier samples that do not resemble any other sample. Please conduct a simulation study with outlying samples to demonstrate.

8. They used a plot of abundance distribution for four taxa (Fig S13) to illustrate the rationale of using Gamma-normal model fitting. For each of six real datasets please give the summary of p-values (LRT for Gamma-normal mixture fitting) for all taxa.

9. When the pvalue for LRT is greater than 0.05, they just let d_ij be 0 and keep the values for that taxon j. In other word, only the taxon with Gamma-normal pattern in abundance distribution can have a chance to be non-biological zero, which limits the entire research.

10. Why are most d_ij concentrated around 0 or 1? A histogram plot of all d_ij values in a study would be helpful.

11. For data pre-processing, normalization is required for mbImpute if the data is not on the same scale. However, for this study, the author mentioned that how to normalize the microbiome data is challenging but it doesn't specify which normalization method is better or worse for mbImpute. Will the difference in normalization affect the performance of the imputation method mbImpute for microbiome data?

Minor:

1. They used MSE for performance comparison. How about RMSE?

2. Why is the precision with imputation lower than the one without imputation (mbImpute+ DESeq2-phyloseq, Figure 2e)? What does it imply?

**Responses to reviewers’ comments on “mbImpute: an accurate and robust imputation method for microbiome data”**

We thank the two reviewers for their insightful comments and suggestions. Our revised text in the manuscript and Additional File 1 are highlighted in blue. Below please find our point-by-point responses.

***Reviewer #1****: This manuscript proposed mbImpute, a novel imputation method to selectively recover observed zero counts in 16S and metagenomic sequence data. It also promotes the DESeq2-phyloseq as the subsequent differential abundance analysis method. This work is important and timely. It can be applied by a wide range of microbiome projects. I can follow the manuscript well. My comments and questions are as follows.*

We thank the reviewer for this positive evaluation of our manuscript.

*1. Are the imputation results impacted by the sequence depth? The manuscript has not discussed this. Based on the default normalization, the depth information is lost, which means the imputed abundances are not affected by the sequence depths. Is this behavior expected?*

*Please comment.*

We thank the reviewer for pointing out this issue. In fact, we expect sequencing depth to have an impact on the imputation accuracy. The rationale is that a deeper sequencing depth would lead to fewer non-biological zeros that need imputation, so the prediction model in mbImpute can be better trained with more data points that do not need imputation. Further, we deem sequencing depth as an informative covariate for identifying similar samples. Hence, we recommend users to include sample sequencing depth as a sample covariate to be leveraged by mbImpute, as we have done in all our real data studies.

To confirm our expectation that deeper sequencing depth would lead to better imputation accuracy, we have added a simulation study to examine how imputation accuracy changes as sequencing depth varies. We have summarized the results on **Page 6** of the main text: “To evaluate the robustness of mbImpute to sequencing depth, we simulate 16S rRNA sequencing data for 300 taxa in 54 samples with four sequencing depths:1000, 2000, 5000, and 10,000 reads per sample (see Additional file 1). Additional file 1: Fig. S5a shows that mbImpute has better imputation accuracy as sequencing depth increases. This is an expected result because a larger sequencing depth leads to fewer missing data so that mbImpute can be better trained with more non-missing data.”

Figure S5a: Distributions of MSE before and after mbImpute is applied to simulated 16S rRNA sequencing data with four sequencing depths (number of reads per sample).

*2. Can you give the definition of Wasserstein distance used in the main texts? I am confused about the main texts "… between the two-dimensional distribution" but the supplementary material mentioned a one-dimensional distribution "\bar{Y_{.1}}^{imp} / sd_{.1}^{imp}.*

We apologize for this confusion. The supplementary statement was accurate. In the revised manuscript, we have changed the main text statement (**Page 5**) to “the Wasserstein distance between the distributions of taxa's abundance mean/(standard deviation) ratios in the imputed data and the complete data.”

*3. "… only DESeq2-phyloseq identifies DA taxa in all datasets." I observed that DESeq2-phyloseq detected >50 DA taxa in Vogtmann CRC study, while other methods detect none. How many DA taxa can be detected by mbImpute-empowered DESeq2 method? To confirm that DESeq2-phyloseq is performing well, authors can include the top findings from DESeq2-phyloseq and mbImpute-empowered DESeq2-phyloseq.*

We thank the reviewer for this suggestion. In our previous version, we included the number of taxa detected by DESeq2-phyloseq and mbImpute-empowered DESeq2-phyloseq from the Vogtmann CRC dataset (**Page 8)** “For the Vogtmann et al. CRC dataset, the 5-fold

cross-validated PR-AUC increases by almost 10% when the DA taxa identified after imputation, instead of those identified before imputation, are used as features. In fact, fewer taxa are identified as DA after imputation (Additional File 1: Fig. S5). At the q-value threshold 0.05, DESeq2-phyloseq identifies 53 DA taxa, while mbImpute-empowered DESeq2-phyloseq identifies 40 DA taxa, with only 17 taxa in overlap. This result suggests that the 23 DA taxa identified only after imputation contribute much to the distinction of control and CRC samples.”

Thanks to the reviewer’s suggestion, in Additional files 2-7 we have included the DA taxa identified by DESeq2-phyloseq and mbImpute-empowered DESeq2-phyloseq, and for each taxon we have included its annotated functional terms that are related to T2D or CRC in the GMrepo database [1]. Moreover, for each T2D- or CRC-related term, we have examined its enrichment in the DA taxa identified by DESeq2-phyloseq or mbImpute-empowered DESeq2-phyloseq (**Page 9**): “For all the DA taxa identified by DESeq2-phyloseq and mbImpute-empowered DESeq2-phyloseq in the two T2D and four CRC data datasets, we query the GMrepo database [1] and find two T2D- and one CRC-related functional terms. For each term, we perform the Fisher's exact test to check its enrichment in the DA taxa identified from the corresponding disease-related datasets. Our results show that all three terms are more enriched in the DA taxa identified after mbImpute is applied (Table 1; Additional Files 1--7), providing functional support to the efficacy of mbImpute in empowering DESeq2-phyloseq.”

Table 1: Fisher's exact test p-values about the enrichment of T2D- and CRC-related functional terms in the DA taxa found by DESeq2-phyloseq or mbImpute-empowered DESeq2-phyloseq. For each term, the DA taxa identified by each method from the corresponding datasets are pooled to do the test.

*T2D term 1: “The time period before the development of symptomatic diabetes. For example, certain risk factors can be observed in subjects who subsequently develop INSULIN RESISTANCE as in type 2 diabetes (DIABETES MELLITUS, TYPE 2).”

**T2D term 2: “A cluster of symptoms that are risk factors for CARDIOVASCULAR DISEASES and TYPE 2 DIABETES MELLITUS. The major components of metabolic syndrome include ABDOMINAL OBESITY; atherogenic DYSLIPIDEMIA; HYPERTENSION; HYPERGLYCEMIA; INSULIN RESISTANCE; a proinflammatory state; and a prothrombotic (THROMBOSIS) state.”

***CRC term: “Tumors or cancer of the COLON or the RECTUM or both. Risk factors for colorectal cancer include chronic ULCERATIVE COLITIS; FAMILIAL POLYPOSIS COLI; exposure to ASBESTOS; and irradiation of the CERVIX UTERI.”

To explain why some taxa were identified as DA before imputation but not DA after imputation, we have examined these taxa’s abundance distributions before and after imputation, and we find that these taxa only differ in zero proportions and have similar non-zero abundance distributions between control and CRC samples. Additional File 1: Fig. S9 shows three example taxa. We argue that such taxa are unlikely to be truly DA because it is questionable whether zero proportion differences are biologically meaningful given the prevalence of technical and sampling zeros.

Figure S9: Abundance distributions of three taxa in control and CRC samples inVogtmann et al.before and after mbImpute is applied.The three taxa, *Alistipes shahii*, *Clostridium citroniae*, and *Flavonifractor plautii*, are identified as DA by DESeq2-phyloseq before imputation but not as DA after imputation. Adjusted p-values are listed.

*4. Page 6, line 52, TEDDY is for Type-1 diabetes. The authors seem to treat it like T2D.*

We thank the reviewer for pointing out our mistake. We have removed the citation to TEDDY in our revision.

*5. The biorxiv version of mbImpute used ZINB-GLM (or NB-GLM) to detect taxa. Can authors comment on why it is not benchmarked in this manuscript?*

We thank the reviewer for reading our bioRxiv version. The reason why we removed the ZINB-GLM and mbImpute+NB-GLM from our submitted manuscript was that after we added the simulated 16S rRNA sequencing data generated by SparseDOSSA, we found that both ZINB-GLM and mbImpute+NB-GLM have almost perfect precision and recall rates on this simulated dataset.

*6. The authors used the number of branches connecting two taxa in the phylogenetic tree as a distance. Have the authors considered directly using distances based on the tree branch lengths?*

We thank the reviewer for this suggestion. In the mbImpute method, we include a parameter \psi to be chosen by cross-validation, and this parameter is designed to adjust the distance (defined as the number of branches connecting two taxa in the phylogenetic tree) by raising it to the power of \psi for better prediction. The reviewer offered an alternative definition of the distance; although we did not use it in our results, we have designed our mbImpute R package to take a user-input taxon distance matrix, so that users can have the flexibility to use the taxon-taxon distance of their choice. We have added the following text to the Discussion section (**Page 14**):

“In the application of mbImpute, users may specify the phylogenetic distances between taxa based on their domain knowledge. In our results, we define the phylogenetic distance between two taxa as the number of branches connecting them in a phylogenetic tree, but alternative choices exist, such as the total lengths of the branches. If users want to choose a distance metric, we recommend that they supply the phylogenetic distances defined by candidate metrics into mbImpute and choose the metric that leads to the smallest cross-validated MSE, i.e., the cross-validated imputation error of mbImpute on non-missing data.”

***Reviewer #2****:*

*The authors proposed an imputation method for microbiome data which have extensive zeros. They used a Gamma-normal mixture model to fit the data and then calculate the probability of missing values which they termed as non-biological zeros. Finally they used a linear model to predict the zero values by borrowing information from similar samples, taxa, and covariates.*

*Through comprehensive simulation studies and real data analyses, the new method, mbimpute, shows better performance than available imputation methods that were designated for other types of data, e.g., single-cell RNA-seq data. The proposed method would be useful in the relevant research area.*

We thank the reviewer for acknowledging the usefulness of our method in the field.

*Major concerns:*

*1. Distinguishing non-biological zeros from biological zeros in metagenomics studies is challenging. If the authors can elaborate that the new method can handle this issue it would be great.*

We agree with the reviewer that it is challenging to distinguish non-biological zeros from biological zeros using a computational approach. Our way of identifying likely non-biological zeros is based on our assumption that each taxon’s log-transformed counts should follow a unimodal distribution if there is no inflation of non-biological zeros. Hence, if a taxon has a bimodal distribution with one mode near zero, we consider the taxon’s zeros that cannot be explained by the non-zero component of the bimodal distribution as likely non-biological zeros.

Using multiple simulation studies, we demonstrate that mbImpute can successfully recover the artificially introduced zeros. In our real data analysis, we show that mbImpute removes zero inflation and restores taxa’s Pearson and Spearman correlations.

*2. Several DA methods have been developed for zero-inflated microbiome data. Please include the Omnibus test (Chen et al. 2018) for comparison.*

Thanks to the reviewer’s suggestion, we have added the Omnibus test into our analysis. We demonstrate that mbImpute empowers the Omnibus test in our 16S rRNA simulation study. The result is consistent across two FDR thresholds, 0.05 (Figure 2e) and 0.1 (see Additional file 1: Figure S4).

Figure 2e: Accuracy (Precision, recall, and F1 scores) of four DA methods (Wilcoxon rank-sum test, ANCOM, metagenomeSeq, DESeq2-phyloseq, andOmnibus test) with the FDR threshold 0.05 on raw data (light color) and imputed data by mbImpute (dark color) in the 16S data simulation.

Figure S4: Accuracy (Precision, recall, and F1 scores) of four DA methods (Wilcoxon rank-sum test, ANCOM, metagenomeSeq, DESeq2-phyloseq, and Omnibus test) with an FDR threshold 0.1 on raw data (light color) and imputed data by mbImpute (dark color) in 16S data simulation.

*3. It seems that for some dataset fewer taxa are detected after applying mbimpute than without imputation, while for some cases more taxa are found for imputed data. Please explain.*

The reviewer is correct that mbImpute does not necessarily increase the number of identified DA taxa. We want to point out that more DA taxa identified is not equivalent to power increase, as the identified DA taxa may include false positives. If a taxon is identified as DA before imputation due to its different zero proportions in two conditions, but its non-zero distributions in the two conditions do not exhibit a significance difference, then it is likely to be identified as non-DA after imputation. We argue that such a taxon is unlikely to be truly DA because the difference in zero proportion is questionably biologically meaningful due to the prevalence of technical and sampling zeros. In the results, we have examined the DA taxa that were identified as DA before imputation but not DA after imputation from the Vogtmann *et al*. CRC dataset. We find that these taxa only differ in zero proportions and have similar non-zero abundance distributions between control and CRC samples, supporting our argument above. Additional File 1: Fig. S9 shows three example taxa.

Figure S9: Abundance distributions of three taxa in control and CRC samples inVogtmann et al.before and after mbImpute is applied.The three taxa, *Alistipes shahii*, *Clostridium citroniae*, and *Flavonifractor plautii*, are identified as DA by DESeq2-phyloseq before imputation but not as DA after imputation. Adjusted p-values are listed.

*4. In the section of "mbImpute empowers DESeq2-phyloseq in DA taxon analysis" they mentioned that there were only 17 taxa in overlap (page 7, line 7). Please display the two sets of taxa in a table, and examine whether there is some taxa (detected by DESeq2-phyloseq but missed by mbImpute-empowered way) that has biologically meaning with the CRC. In other word, due to the imputation some true signal might be missed/distorted.*

Thanks to the reviewer’s suggestion, in Additional files 2-7 we have included the DA taxa identified by DESeq2-phyloseq and mbImpute-empowered DESeq2-phyloseq, and for each taxon we have included its annotated functional terms that are related to T2D or CRC in the GMrepo database [1]. Moreover, for each T2D- or CRC-related term, we have examined its enrichment in the DA taxa identified by DESeq2-phyloseq or mbImpute-empowered DESeq2-phyloseq (**Page 9**): “For all the DA taxa identified by DESeq2-phyloseq and mbImpute-empowered DESeq2-phyloseq in the two T2D and four CRC data datasets, we query the GMrepo database [1] and find two T2D- and one CRC-related functional terms. For each term, we perform the Fisher's exact test to check its enrichment in the DA taxa identified from the corresponding disease-related datasets. Our results show that all three terms are more enriched in the DA taxa identified after mbImpute is applied (Table 1; Additional Files 1-7), providing functional support to the efficacy of mbImpute in empowering DESeq2-phyloseq.”

Table 1: Fisher's exact test p-values about the enrichment of T2D- and CRC-related functional terms in the DA taxa found by DESeq2-phyloseq or mbImpute-empowered DESeq2-phyloseq.

For each term, the DA taxa identified by each method from the corresponding datasets are pooled to do the test.

*T2D term 1: “The time period before the development of symptomatic diabetes. For example, certain risk factors can be observed in subjects who subsequently develop INSULIN RESISTANCE as in type 2 diabetes (DIABETES MELLITUS, TYPE 2).”

**T2D term 2: “A cluster of symptoms that are risk factors for CARDIOVASCULAR DISEASES and TYPE 2 DIABETES MELLITUS. The major components of metabolic syndrome include ABDOMINAL OBESITY; atherogenic DYSLIPIDEMIA; HYPERTENSION; HYPERGLYCEMIA; INSULIN RESISTANCE; a proinflammatory state; and a prothrombotic (THROMBOSIS) state.”

***CRC term: “Tumors or cancer of the COLON or the RECTUM or both. Risk factors for colorectal cancer include chronic ULCERATIVE COLITIS; FAMILIAL POLYPOSIS COLI; exposure to ASBESTOS; and irradiation of the CERVIX UTERI.”

*5. In the section of "mbImpute preserves distributional characteristics of taxa's non-zero abundances and recovers downsampling zeros" they used Pearson correlation. A non-parametric correlation might be more appropriate when there are extreme values in microbial data, which is quite often.*

We thank the reviewer for this comment, and we have added the results based on Spearman correlations to this section. The results are consistent with our previous results based on Pearson correlations. We have added the following sentences (**Page 12**): “Note that the resemblance is defined based on the Pearson correlation of two sets of correlations. Additional File 1: Fig. S14 shows that the same conclusion holds when the resemblance is defined based on the Spearman correlation.” For the reviewer’s convenience, below we have also pasted Fig. 4c about the Pearson correlations for comparison.

Figure S14: Each bar shows the Spearman correlation between taxon-taxon correlations in raw data (light gray) or imputed data (dark gray) using all samples and taxon-taxon correlations in raw data using non-zero samples only. The two correlations are calculated for two T2D datasets and four CRC datasets using diseased samples, control samples, and whole data.

Figure 4c: Each bar shows the Pearson correlation between taxon-taxon correlations in raw data (light gray) or imputed data (dark gray) using all samples and taxon-taxon correlations in raw data using non-zero samples only. The two correlations are calculated for two T2D datasets and four CRC datasets using whole data, diseased samples, and control samples.

*6. In the section of "mbImpute increases the similarity of microbial community structure between 16S rRNA and WGS data" they used two stool samples, from these two types of technologies. What would happen for two quite different samples, e.g., one from a natural environment using 16S rRNA and one from a human gut based on WGS? Would the similarity between these two samples increase after imputation? If so, the increased similarity due to the imputation does not reflect the true relationship between the samples. They need to focus on the similarity between the samples (different or alike) under the same sequence technology and examine the impact of imputation.*

We thank the reviewer for this nice suggestion and we have added a negative control study based on the oral 16S samples and stool WGS samples (**Pages 13-14**): “We perform a negative control study to confirm that the increased similarity between 16S rRNA and WGS data is not an artifact introduced by mbImpute. We use a 16S rRNA dataset of human oral samples and a WGS dataset of human stool samples, which are expected to have different genus-level taxon-taxon correlations. Same as in the previous study, we compare the genus-level taxon-taxon correlations between the two datasets before and after applying mbImpute.

Additional File 1: Fig. S16 shows that mbImpute decreases the similarity between the taxon correlation matrices of the two datasets. Before imputation, the Pearson correlation between the two correlation matrices is 0.21; mbImpute decreases the correlation to 0.09.”

Figure S16: Four Pearson correlation matrices are calculated based on a common set of genus-level taxa’s abundances in 16S healthy human oral samples and WGS healthy human stool samples, with or without using mbImpute as a preceding step. Before imputation, the Pearson correlation between the two correlation matrices is 0.21, and this correlation decreases to 0.09 after imputation. For illustration purposes, each heatmap shows square roots of Pearson correlations, with the bottom 40% of values truncated to 0.

*7. They claimed that mbImpute is robust to the existence of outlier samples that do not resemble any other sample. Please conduct a simulation study with outlying samples to demonstrate.*

We have conducted a simulation study to verify the robustness of mbImpute to outlier samples

(**Page 6**): “We also check the robustness of mbImpute to outlier samples. Taking the sample with the 2000-read per-sample sequencing depth, we generate one or two outlier samples by assigning large abundance values to 62 lowly abundant taxa in the existing 54 samples and setting other taxa's abundance to zero (see Additional File 1). Additional File 1: Fig. S5b shows that the imputation accuracy of mbImpute is robust to the introduction of outlier samples.

Additional File 1: Fig. S6 shows the abundance distributions of four example taxa with outlier values before and after imputation. We observe that the existence of outliers does not distort the post-imputation distribution of non-outlier samples.”

Figure S5b: Distributions of MSE before and after mbImpute with the 2,000-read per-sample sequencing depth and 0, 1 or 2 outlier samples added.

Figure S6: Abundance distributions of four taxa with one or two outlier samples before and after mbImpute isapplied.For each taxon, the top row (”raw”) displays the abundance distributions with 0, 1, or 2 outlier samples before imputation, and the bottom row (”imp”) shows the distributions after mbImpute is applied. The abundance values in the outlier samples are marked by red circles.

*8. They used a plot of abundance distribution for four taxa (Fig S13) to illustrate the rationale of using Gamma-normal model fitting. For each of six real datasets please give the summary of p-values (LRT for Gamma-normal mixture fitting) for all taxa.*

We have added the following summary of LRT p-values (**Page 18-19**): “We summarize the LRT p-values calculated on six real WGS datasets and observe that few taxa have p-values greater than 0.05 (see Additional File 1: Fig.~S20a). Additional File 1: Fig.~S20b shows the distribution of one randomly picked taxon with LRT p-value greater than 0.05 in each dataset; these taxa's log-transformed counts do not have a mode close to zero.”

Figure S20: Outputs of step 1 of mbImpute on six real datasets. (a)Distributions of the LRT p-values in step 1 of mbImpute. Most p-values are close to 0. For each dataset, the percentage of taxa with >0.05 p-values are shown at the top right. (b) Distributions of six example taxa, each of which has the LRT p-value >0.05 in each dataset. For these taxa, mbImpute does not perform imputation.

*9. When the pvalue for LRT is greater than 0.05, they just let d_ij be 0 and keep the values for that taxon j. In other words, only the taxon with Gamma-normal pattern in abundance distribution can have a chance to be non-biological zero, which limits the entire research.*

As we have shown in Additional file 1: Figure S20, the percentage of taxa that have p-values greater than 0.05 is small in each dataset. Hence, only a small percentage of taxa would be omitted by mbImpute, thus not limiting the effectiveness of mbImpute.

*10. Why are most d_ij concentrated around 0 or 1? A histogram plot of all d_ij values in a study would be helpful.*

Additional file 1: Figure S21 shows the histogram of d_ij values for each of the 6 datasets. The reason for this phenomenon is that most taxa exhibit clear bimodal distributions (i.e., the Gamma and Normal density functions have small overlap area) for their log-transformed abundances. Hence, for such a taxon j, its abundance in sample i can be assigned to either component (Gamma or Normal) with a high confidence, leading to a d_ij value close to 1 or 0.

Figure S21: The distributions of d_ij's calculated in step 1 of mbImpute applied to six real datasets. The 0.5 threshold is indicated by the red dotted line.

*11. For data pre-processing, normalization is required for mbImpute if the data is not on the same scale. However, for this study, the author mentioned that how to normalize the microbiome data is challenging but it doesn't specify which normalization method is better or worse for mbImpute. Will the difference in normalization affect the performance of the imputation method mbImpute for microbiome data?*

The goal of normalization is to make taxon counts comparable across samples, a necessary assumption of mbImpute. In our results, we think our way of normalization is sufficient to meet this assumption. However, the appropriate normalization method for mbImpute is case by case in future applications, depending on whether confounders such as batch effects are observable; hence, users' judgement is indispensable. We recognize that benchmarking normalization methods for microbiome data is a separate project. Hence, we refer users to benchmark papers [2,3] to guide their choice of benchmark methods. We have added this discussion to our main text.

*Minor:*

*1. They used MSE for performance comparison. How about RMSE?*

Since RMSE is the square root of MSE, using RMSE will not change the conclusions of our comparison results in the paper. Hence, we have decided not to add the RMSE comparison. *2. Why is the precision with imputation lower than the one without imputation (mbImpute+ DESeq2-phyloseq, Figure 2e)? What does it imply?*

There is often a trade-off between precision and recall. By definition, precision is the proportion of true DA taxa among the identified DA taxa, while recall (i.e., power) is the proportion of true DA taxa that have been identified. When the number of identified DA taxa increases, the recall always increases, but the precision may decrease if the identified DA taxa include many false positives. Because of this trade-off, the F1 score is used to represent the overall accuracy. Figure 2e shows that mbImpute consistently increases the recall; although mbImpute does not always increase the precision, the F1 score shows that mbImpute consistently improves the overall accuracy.

**References**

1. Wu, S., Sun, C., Li, Y., Wang, T., Jia, L., Lai, S., ... & Chen, W. H. (2020). GMrepo: a database of

curated and consistently annotated human gut metagenomes. *Nucleic acids research*, *48*(D1),

D545-D553.

2. Calgaro, M., Romualdi, C., Waldron, L., Risso, D., & Vitulo, N. (2020). Assessment of statistical

methods from single cell, bulk RNA-seq, and metagenomics applied to microbiome data. *Genome*

*biology*, *21*(1), 1-31.

3. McKnight, D. T., Huerlimann, R., Bower, D. S., Schwarzkopf, L., Alford, R. A., & Zenger, K. R.

(2019). Methods for normalizing microbiome data: an ecological perspective. *Methods in Ecology*

*and Evolution*, *10*(3), 389-400.

**2^nd^ round**

**Reviewer 2**

1. Please include other imputations methods for comparison in Figure S5a: MSE plot for before and after imputation.

2. You mentioned in the response that “we find that these taxa only differ in zero proportions and have similar non-zero abundance distributions between control and CRC samples … We argue that such taxa are unlikely to be truly DA because it is questionable whether zero proportion differences are biologically meaningful given the prevalence of technical and sampling zeros.”

The proportion of zeros matters, e.g, abundance of a species in 10 control samples is (0, 0, 0, 0, 0, 0, 0, 0, 0, 10) while in 10 case samples is (0, 10, 10, 10, 10, 10, 10, 10, 10, 10). The different zero proportions (10% vs 90%) in two conditions contribute to the (biological) difference. You may find some literatures in DA for microbial studies regarding this issue. That is also why the omnibus test was proposed by Chen et al. for DA analysis.

3. As the distance based on the tree branch lengths can provide more and accurate information than the number of branches connecting two taxa in the phylogentic tree, it would be very beneficial to the users later if the authors can provide this option in their R package, rather than just let the users to calculate the phylogenetic distances by themselves.

4. You used MSE for performance comparison. How about RRMSE (relative root MSE)?

**Authors’ response**

**Reviewer 2:**

*1. Please include other imputations methods for comparison in Figure S5a: MSE plot for before and after imputation.*

We thank the reviewer for this suggestion, and we have included the five other imputation methods (softImpute and four scRNA-seq imputation methods) in this comparison. The results are shown in Additional file 1: Fig. S6 (in which the results of the baseline and mbImpute are the same as those in Fig. S5a), and we have summarized the results on **Page 6 of the main text**:

“We further evaluate the performance of the five non-microbiome imputation methods along with mbImpute. Additional File 1: Fig. S6 shows that softImpute and ALRA, the two low-rank matrix factorization methods, also have better imputation accuracy as sequencing depth increases, yet their accuracies are worse than those of mbImpute at all sequencing depths. Unexpectedly, the four other imputation methods developed for single-cell RNA-seq data—SAVER, scImpute, MAGIC, and ALRA—show no improvement over the baseline, “no imputation”. One possible reason is that the sequencing depths used in this simulation (~10^3) are much lower than those of typical scRNA-seq data (~10^6). These results again suggest that scRNA-seq imputation methods are unsuitable for microbiome 16S rRNA sequencing data.”

*2. You mentioned in the response that “we find that these taxa only differ in zero proportions and have similar non-zero abundance distributions between control and CRC samples … We argue that such taxa are unlikely to be truly DA because it is questionable whether zero proportion differences are biologically meaningful given the prevalence of technical and sampling zeros.”*

*The proportion of zeros matters, e.g, abundance of a species in 10 control samples is (0, 0, 0, 0, 0, 0, 0, 0, 0, 10) while in 10 case samples is (0, 10, 10, 10, 10, 10, 10, 10, 10, 10). The different zero proportions (10% vs 90%) in two conditions contribute to the (biological) difference. You may find some literatures in DA for microbial studies regarding this issue. That is also why the omnibus test was proposed by Chen et al. for DA analysis.*

We thank the reviewer for pointing this out. In our previous manuscript, we had summarized the different perspectives about microbiome DA taxa identification in the Additional file 1: Statistical definitions of DA taxa (please see screenshots below). To avoid readers’ confusion about this issue, we have added comments to **Discussion in the main text**: “Regarding the mbImpute-empowered DA analysis, we note that it offers a new perspective of identifying DA taxa from microbiome 16S and WGS data after imputation. We have summarized three statistical definitions of DA taxa in microbiome data in Additional file 1: Statistical definitions of DA taxa. Note that mbImpute-empowered DA analysis is advantageous in that it alleviates the existence of non-biological zeros, and it uses all available samples for DA testing. A controversial question is, if a taxon has few zeros in condition 1 but few non-zeros in condition 2, and the non-zero values have similar magnitudes in the two conditions, whether or not should this taxon be called DA. We note that mbImpute is unlikely to impute the predominant zeros in condition 2 because it would treat these zeros as biological zeros. Hence, mbImpute-empowered DA analysis is likely to call such a taxon as DA.”

*3. As the distance based on the tree branch lengths can provide more and accurate information than the number of branches connecting two taxa in the phylogenetic tree, it would be very beneficial to the users later if the authors can provide this option in their R package, rather than just let the users to calculate the phylogenetic distances by themselves.*

We thank the reviewer for this nice suggestion, and we want to point out that our R package takes any user-specified distance matrix, whose entries are pairwise taxon-taxon phylogenetic distances. We have added the following sentence to **Methods in the main text**: “(in our R package implementation, the mbImpute function can take any distance matrix D as input that reflects the relationship among taxa specified by the user.)”. In our current R package, we have implemented a function for converting a phylogenetic tree’s edge set (edges without length information) into a distance matrix. We will consider updating this function by incorporating edge lengths in a later version of our package.

*4. You used MSE for performance comparison. How about RRMSE (relative root MSE)?*

We did not add RRMSE because it would have the same trends as those of MSE. Hence, adding RRMSE or not would not change the conclusions in our manuscript.
